# Remodeling of extracellular matrix collagen IV by MIG-6/papilin regulates neuronal architecture

**DOI:** 10.21203/rs.3.rs-5962240/v1

**Published:** 2025-02-14

**Authors:** Malika NADOUR, Robert I. VALETTE REVENO LEATIS, Marie BIARD, Noémie FRÉBAULT, Lise RIVOLLET, Philippe ST-LOUIS, Cassandra R. BLANCHETTE, Andrea THACKERAY, Paola PERRAT, Carlo BEVILACQUA, Robert PREVEDEL, Laurent CAPPADOCIA, Georgia RAPTI, Maria DOITSIDOU, Claire Y. BÉNARD

**Affiliations:** 1 Université du Québec à Montréal, Department of Biological Sciences, Montreal, QC, Canada; 2 Centre d’Excellence en Recherche sur les Maladies Orphelines – Fondation Courtois (CERMO-FC Research Center), Université du Québec à Montréal, Montreal, QC, Canada; 3 University of Massachusetts Chan Medical School, Department of Neurobiology, MA, USA; 4 Cell Biology and Biophysics Unit, European Molecular Biology Laboratory, Heidelberg, Germany; 5 Developmental Biology Unit, European Molecular Biology Laboratory, Heidelberg, Germany; 6 Epigenetics and Neurobiology Unit, European Molecular Biology Laboratory, Rome, Italy; 7 Interdisciplinary Center of Neurosciences, Heidelberg University, Heidelberg, Germany; 8 Université du Québec à Montréal, Department of Chemistry, Montreal, QC, Canada; 9 Quebec Network for Research on Protein Function, Engineering and Applications (PROTEO), QC, Canada; 10 FENS-KAVLI Network of Excellence, Brussels, Belgium; 11 University of Edinburgh, Centre for Discovery Brain Sciences, Edinburgh, Scotland

**Keywords:** Papilin, MIG-6, collagen IV, extracellular matrix, neuronal maintenance

## Abstract

Neuronal architecture established embryonically must persist lifelong to ensure normal brain function. However, little is understood about the mechanisms behind the long-term maintenance of neuronal organization. To uncover maintenance mechanisms, we performed a suppressor screen in *sax-7*/*L1CAM* mutants, which exhibit progressive disorganization with age. We identified the conserved extracellular matrix protein MIG-6/papilin as a key regulator of neuronal maintenance. Combining incisive molecular genetics, structural predictions, *in vivo* quantitative imaging, and cutting-edge Brillouin microscopy, we show that MIG-6/papilin remodels extracellular matrix collagen IV, working in concert with the secreted enzymes MIG-17/ADAMTS and PXN-2/peroxidasin. This remodeling impacts tissue biomechanics and ensures neuronal stability, even under increased mechanical stress. Our findings highlight an extracellular mechanism by which MIG-6/papilin supports the integrity of neuronal architecture throughout life. This work provides critical insights into the molecular basis of sustaining neuronal architecture and offers a foundation for understanding age-related and neurodegenerative disorders.

## INTRODUCTION

Proper nervous system function depends on both developing and maintaining the intricate architecture of its neural circuits. The dynamic processes of development and maturation of the nervous system begin *in utero* and extend well into adulthood ^1^. Key neuronal features, established earlier in development, must be preserved throughout life to ensure continuity in neuronal architecture and function ^2^. The structural organization of the nervous system faces numerous challenges, including physical stresses induced by the organism’s postnatal growth, anatomical remodeling, and the integration of new neurons, body movements, and injury ^3^. Failure to stably maintain neuronal architecture over the long-term can impair and compromise neuronal function and contribute to the manifestation of neurological conditions ^4,5^. Notably, a defining feature of many neurodegenerative diseases is the destabilization of axons and dendrites, and accompanying loss of synapses ^6–8^. Gaining insights into the mechanisms that preserve nervous system architecture could inform the development of therapeutics aimed at preventing or reversing such neurological conditions. Despite their importance, the mechanisms that sustain neuronal organization throughout life remain poorly understood.

A key contribution to the preservation of the structural integrity of multicellular assemblies, including those in the nervous system, comes the extracellular matrix (ECM) ^9^. Cells interact with the ECM and neighboring cells via cell adhesion molecules, and the coordinated actions of these molecules and the ECM are key for cellular behavior, enabling, for instance, neuronal structures to withstand physical stresses ^5^. However, how cellular regulation implicating ECM interactions evolve through time, or when subjected to physical challenges, remains elusive.

The ECM is of crucial importance in the nervous system, playing critical roles during development ^10^, but also in the adult mammalian brain, where the ECM constitutes approximately 20% of its volume. Diffuse ECM is found near and between synapses across the brain, and condensed ECM is organized as basement membranes associated with blood vessels, or as lattice-like structures called perineuronal nets, surrounding the soma and dendrites of several neuron types in multiple brain regions ^11^. These organized forms of ECM influence neuronal biology in the adult brain, including dendritic spine stability, synapse plasticity, and axon regeneration ^11,12^. Also, changes in ECM composition and structure have been linked to various neurological diseases (e.g., Alzeihmer’s disease and schizophrenia), as well as to brain injury and aging ^13,14^. This underscores the critical role of the neurons’ extracellular environment in maintaining normal neuronal physiology and its involvement in pathological conditions ^5^. However, our understanding of the long-term regulation of the ECM in the mature nervous system is limited and remains a dauting problem for neuropathology ^15^. For instance, how the intricate interactions between ECM proteases and their substrates, regulate ECM dynamics for sustaining neuronal structure and function is poorly understood.

The nematode *C. elegans* provides a powerful *in vivo* genetic model to study the lifelong maintenance of nervous system architecture, and particularly the role of the ECM in this process. A significant number of *C. elegans* neurons are organized into ganglia, with most neuronal processes running along major fascicles, such as the neuropil and the ventral nerve cord ^16^. The multicellular assemblies of ganglia and nerve cords are ensheathed by a specialized ECM, namely basement membranes ^17^. After hatching into a larva, *C. elegans* undergoes a nearly 100-fold increase in size until it reaches adulthood ^18^. Yet, the overall architecture of its nervous system is established during embryogenesis and remains largely intact throughout post-embryonic growth ^17,19^. Indeed, serial-section electron microscopy and connectome reconstruction of multiple *C. elegans* brains at successive stages of development revealed that the shape and positioning of most neurons and neurites established at birth remains consistent through adulthood, including approximately 70% of adult brain synapses being part of stable connections that are proportionally maintained from birth to adulthood ^19^. Individual neurons and ECM components can be readily visualized in living animals throughout their lifespan, using fluorescent reporters thanks to *C. elegans* transparency and small size ^20,21^. Combined with its genetic tractability, including using cell-specific promoters and conditional knockdowns, these features enable the investigation of the mechanisms sustaining nervous system architecture across its lifetime.

Thus, *in vivo* genetic studies using *C. elegans* has yielded critical insights into the mechanisms of the long-term maintenance of neuronal assemblies. These investigations have revealed post-natal molecular mechanisms that actively preserve neuronal organization, particularly within ganglia and nerve cords. Notably, several immunoglobulin superfamily molecules play crucial roles in maintaining the architecture of these multi-neuronal assemblies over time. These include the cell adhesion molecule SAX-7/L1CAM ^22–27^, the large ECM protein DIG-1 ^28,29^, the secreted two-immunoglobulin domain containing proteins ZIG-3 and ZIG-4 ^30,31^, and the ectodomain of the FGF receptor EGL-15 ^32^. Mutations in the genes encoding these molecules lead to neuronal defects that arise later in development, well after the normal initial establishment of neuronal morphology of the affected neurons. Strikingly, neuronal maintenance defects are suppressed by paralysis, highlighting that neuronal structures experience internal mechanical stress generated by body or organ movements, and that the identified neuronal maintenance molecules counteract these stresses, which otherwise would lead to neuronal disorganization ^28,31,33^. Orthologues of some of these molecules have been found to sustain neural circuits in other systems as well; for instance, in mice, knockout of L1CAM specifically in the adult brain results in behavioral deficits and synaptic transmission changes ^34^, and L1CAM maintains neocortical axo-axonic innervation into adulthood ^35^. While we have evidence that cell adhesion molecule SAX-7/L1CAM and ECM molecule DIG-1 play important roles in maintaining nervous system architecture, how the underlying ECM landscape and molecular interactions contribute to sustaining nervous system architecture throughout life is not understood. Given the extensive evolutionary conservation of ECM and neuronal cell surface molecules from worms to mammals, the maintenance mechanisms unravelled in *C. elegans* will provide insights on general principles by which the nervous system architecture is preserved lifelong.

To expand our understanding of the molecular mechanisms governing the preservation of neuronal architecture throughout life, we conducted a forward genetic suppressor screen in the *sax-7* mutant background, and identified *mig-6*, which encodes papilin, an extracellular matrix protein with structural similarities with ADAMTS metalloproteases ^36^. Papilin is conserved across metazoans, but its function remains elusive. Our findings reveal that MIG-6S/papilin is required post-developmentally for neuronal maintenance, by modulating the major extracellular matrix component collagen IV. Using Brillouin microscopy, we find that loss of *mig-6* changes the biomechanical properties of tissues in the region comprising the neurons. We demonstrate that MIG-6S/papilin cooperates with the ECM remodeling metalloproteinase MIG-17/ADAMTS in regulating the collagen IV network, and that both collagen IV levels and crosslinking are critical to sustain neuronal organization lifelong. Our study underscores the critical role of ECM regulation by the extracellular matrix protein papilin in preserving neuronal architecture throughout an organism’s lifetime, including under conditions of significant mechanical stress. We propose a model in which a balance between flexibility and adhesion, mediated by ECM remodeling and cell adhesion, ensures the structural stability of the embryonically established nervous system over time and improve its ability to withstand mechanical stress.

## MATERIALS AND METHODS

Please see Supplementary Information.

### Data Availability Statement:

All data is available in the main text or the supplementary materials.

## RESULTS

### Loss of function of *mig-6*, which encodes the conserved extracellular matrix protein papilin, suppresses neuronal maintenance defects of *sax-7* mutants

**Figure 1.**
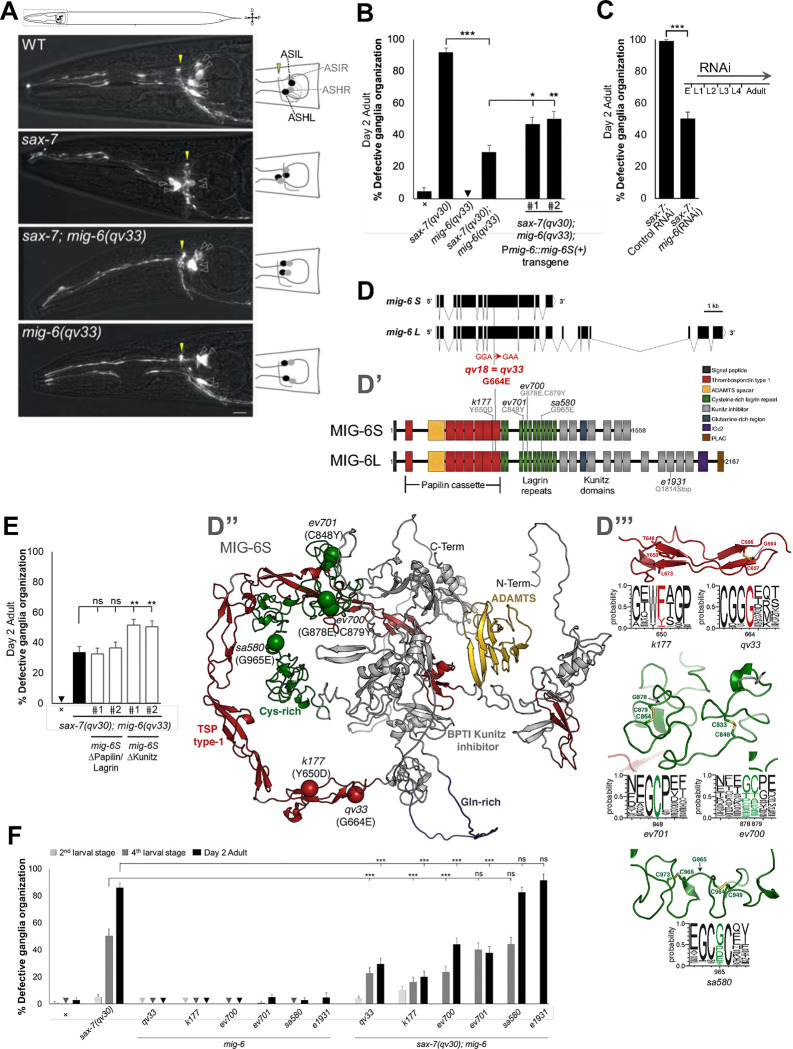
Loss of function of *mig-6*, which encodes the conserved ECM protein papilin, suppresses neuronal maintenance defects of *sax-7* mutants. (**A**) *qv33* is a newly identified allele of *mig-6* that suppresses the neuronal defects of *sax-7* mutants. Fluorescence images of the head region of 2-day-old adults (as indicated on worm schematics); chemosensory neurons ASH and ASI were visualized using reporter P*sra-6::DsRed2* (soma indicated by empty arrow heads; schematized on the right). In the wild type, the soma of the four neurons (each ASH and ASI pair has a soma in the left ganglion and in the right ganglion), are positioned posterior to the nerve ring (indicated by the yellow arrowhead) throughout life. In *sax-7* mutants, ASH and ASI soma are initially positioned normally but by the 4th larval stage or older they become progressively mispositioned relative to the nerve ring. Mutation *mig-6(qv33)* suppresses the neuronal disorganization of *sax-7(qv30)* mutants. Scale bar, 10 μm. (**B**) Quantification of the neuronal disorganization of ASH and ASI neurons (depicted in **A**), including in double mutants *sax-7; mig-6*, expressing a transgene of *mig-6S(+)* under the gene’s endogenous promoter which rescues the *mig-6*-mediated suppression of *sax-7-*neuronal disorganization defects. (**C**) Post-developmental depletion of *mig-6* by RNAi (from the 1st larval stage onwards) suppresses *sax-7* neuronal maintenance defects. (**D**) Schematic structure of the two isoforms of the gene *mig-6*, which encode a short and a long isoform of MIG-6/papilin (**D’**). The new allele *mig-6(qv33)* is a G664E amino acid substitution. Previously identified *mig-6* alleles are also indicated. (**D”**) Overall structure of the MIG-6S protein predicted using AlphaFold version 2.3.1 implemented in ColabFold 1.5.2, with protein domains are colored as in **D’**. *qv33* and other mutations used are indicated. (**D’”**) Structural environment of residues Y650 (top left), G664 (top right), C848 (middle left), G878 and C879 (middle right), and G965 (bottom) in the structure of the MIG-6S protein predicted using ColabFold 1.5.2. These residues, and selected neighboring residues, are in stick representation. Residue conservation at each position is indicated through sequence logos generated using Weblogo 3.7.12 and an alignment of 250 protein sequences from Ecdysozoa species, including nematodes and arthropods, obtained using MMSeqs2 14-7e284. (**E**) Quantification of rescue assays with transgenes encoding recombinant versions of *mig-6S* (Δ indicates deleted domain). (**F**) Quantification of head ganglia organization phenotype in different *mig-6* alleles alone or in the *sax-7(qv30)* mutant background at the 2nd and 4th larval stages and 2-day-old adults. Age-progressive neuronal disorganization of *sax-7(qv30)* mutants is suppressed by some, but not all, *mig-6* alleles. Error bars are the standard error of the proportion. Comparisons made with z-tests; *P*-values were corrected by multiplying by the number of comparisons, Bonferroni correction. **Note**: Throughout all figures of this work, asterisks denote significant difference (*P ≤ 0.05, **P ≤ 0.01, ***P ≤ 0.001); n.s., not significant; appropriate *post hoc* tests were performed for multiple comparisons (see Materials and Methods); “+” indicates wild-type strain. Sample sizes and source data in Supplementary Information.

To identify novel genes involved in the long-term maintenance of neuronal organization, we conducted a forward genetic screen. We refrained from searching directly for mutants with neuronal maintenance defects, as previous efforts using this approach invariably yielded numerous alleles of the large neuronal maintenance gene *dig-1* (^28^; C.Y.B., unpub. results). Rather, we reasoned that screening for suppressors of the defects of previously known neuronal maintenance mutants (Fig. S1), *sax-7*, would identify genes that directly or indirectly counteract defective long-term maintenance of neuronal architecture, providing insights into the basis of this process. In wild-type animals, the soma of chemosensory neurons ASH and ASI are located posterior to the nerve ring, where their axons project (neurons visualized with reporter P*sra-6::*DsRed2, Fig. 1A)^27^. This stereotypical positioning acquired during embryogenesis is preserved throughout life, making it a reliable indicator of neuronal organization. In *sax-7* mutants, although the soma and axons of ASH/ASI initially exhibit normal positioning during earlier development, they later become displaced from the 4th larval stage onward (Fig. 1A,B,F) ^22,25,27^, with the ASH/ASI soma ending up anteriorly displaced, and the nerve ring shifting posteriorly, resulting in the soma aligning with or even anterior to the nerve ring (Fig. 1A). In our F2 clonal genetic screen for suppressors of *sax-7* neuronal maintenance defects, we mutagenized *sax-7* mutants with ethyl methanesulfonate and screened F3 broods by fluorescence microscopy to find suppressors of the *sax-7* mutants ASH/ASI position defects. We isolated mutation *qv18*, which significantly suppressed the neuronal position defect in adult *sax-7(qv24)* animals, thus reducing the incidence of animals with mispositioned neurons (Fig. S1).

Through whole genome sequencing and bioinformatic analyses ^37–39^, we identified as a candidate mutation a G-to-A transition at transcript nucleotide 1991 of the gene *mig-6*, which causes a glycine to glutamic acid substitution at residue 664 (Fig. 1D’). To determine if this mutation was the causal suppressor, we used CRISPR-Cas9 technology to reintroduce the candidate *qv18* molecular lesion in the *sax-7(qv30)* null mutant background ^27^. The resulting allele of *mig-6*, *qv33*, profoundly suppresses the *sax-7(qv30)* defects, reducing the percentage of affected animals from 90% to 30% (Fig. 1 A,B), indicating that the suppressor mutation is indeed an allele of the gene *mig-6*. Except for gonad defects (similarly found in previously reported *mig-6* mutations) ^40^, single mutant *mig-6(qv33)* animals are fully viable and fertile, displaying a wild-type phenotype including for neurons ASH and ASI, overall nervous system morphology, body wall musculature, and pharynx (Figs. 1A,B, S2, S6). In contrast, null alleles of *mig-6* are sterile and embryonic and larval lethal ^40^. Knockdown of *mig-6* by RNA interference (RNAi) mimicked the effect of *mig-6(qv33)*, significantly suppressing *sax-7* neuronal defects (Fig. 1C). This result further confirms our molecular identification of the suppressor, and indicates that *qv33* is a loss-of-function mutation.

### The short isoform MIG-6S is key in neuronal maintenance, acting through its papilin cassette and lagrin repeats

The gene *mig-6* encodes a short isoform, *mig-6S*, and a long isoform, *mig-6L* (Fig. 1D) ^40^. We used five other *mig-6* alleles previously studied in the context of distal tip cell migration ^40^ to analyze their effect on *sax-7-*mediated neuronal maintenance (Fig. 1D’, F). An allele that specifically affects *mig-6L*, *e1931*, failed to suppress the neuronal maintenance defects in *sax-7(qv30); mig-6(e1931)* (Fig. 1F), indicating that *mig-6L* is not implicated in this context. In contrast, other *mig-6* alleles that like *qv33* affect both the short and the long isoforms (*k177, ev700* and *ev701*) profoundly suppressed the ASH/ASI neuronal maintenance defects in *sax-7(qv30); mig-6* double mutant animals (Fig. 1F). We further validated that *mig-6S* is the functional isoform in neuronal maintenance by performing rescue assays using a *mig-6S* transgene expressed under its endogenous promoter (minigene pZH125 ^40^). Since the loss of *mig-6* suppresses *sax-7* neuronal maintenance defects, restoration of *mig-6* function in double mutant animals *sax-7; mig-6* is expected to result in the reappearance of *sax-7* defects. Transgenic *sax-7(qv30); mig-6(qv33)* animals carrying wild-type transgenic copies of *mig-6S(+)* showed partial but significant rescue (Fig. 1B). The semi-dominant behavior of *mig-6* mutations described in other contexts ^40^ and in our analyses (below, Figs. 3,5) could explain the partial rescue. Collectively, these results firmly establish that *mig-6S* is central in neuronal maintenance.

*mig-6* encodes the conserved extracellular matrix MIG-6/papilin, which is orthologous to *Drosophila* and vertebrate papilin ^36,40–43^. Papilin is important for proper organogenesis in flies ^36^ and for gonad and pharynx development in worms ^21,40,44^ but its role in the nervous system remains largely unknown. Recently *papilin* was isolated in a screen for brain morphogenesis mutants, but its function awaits study ^45^, and in *C. elegans*, loss of *mig-6* was shown to affect PVD neuron 1o dendrite development ^46^. Little is known about the molecular mechanism of papilin function. MIG-6/papilin is a multidomain glycoprotein harboring thrombospondin type 1 (TSP1) repeats, cysteine rich lagrin repeats, and Kunitz protease inhibitor domains, among other domains (Fig. 1D’ ^36,43^, and belongs to the ADAMTSL family of proteins (A Disintegrin and Metalloproteinase with Thrombospondin motifs-Like). Papilins, as other ADAMTSL proteins, are structurally related to ADAMTS metalloproteinases but lack the catalytic domain characteristic of ADAMTS, and no catalytic activity has been reported ^47,48^. Importantly, ADAMTSL proteins are characterized by the “papilin cassette" (Fig. 1D’), a region containing TSP1 domains and an ADAMTS spacer (homologous to non-catalytic domains of ADAMTS metalloproteinases) ^36,43^. Yet, the papilin cassette present in *Drosophila* papilin has been shown, *in vitro*, to bind and inhibit the activity of a procollagen N-proteinase, a vertebrate ADAMTS ^36^. In the *C. elegans* gonad, *mig-6* function influences the localization and levels of ADAMTS proteins MIG-17 and GON-1 ^21,40^. This raises the possibility that MIG-6/papilin may play important roles in the extracellular matrix via its papilin cassette.

As a first step to identify the critical domains of MIG-6S in neuronal maintenance, we modeled the molecular consequences of these tested *mig-6* alleles on MIG-6S. The *mig-6(qv33)* mutation results in a substitution of a glycine to a glutamic acid at amino acid 664 (G664E), located in a TSP1 repeat in the papilin cassette. A sequence alignment as well as the predicted structure of MIG-6S, modeled using ColabFold (Fig. 1D”, S3), shows that G664 is a highly conserved residue located at the entrance of a beta-strand (Fig. 1D”’). Replacement of a glycine with a larger residue in the *qv33* mutant is likely to cause a steric clash with the disulfide bond formed by residues C657 and C696 (Fig. 1D’”) and could alter a potential binding of MIG-6S to the ECM, or other interactions.

Similarly, other *mig-6* alleles that suppress *sax-7* defects (Fig. 1F) affect residues located in the papilin cassette or in the adjacent lagrin repeats (Fig. 1D’, D”). Indeed, *mig-6(k177)* (Y650D) missense allele impacts a residue in a TSP1 domain of the papilin cassette. In the ColabFold predicted structure of MIG-6S, the aromatic portion of Y650 interacts with both L673 and T648 (Fig. 1D’”). Consistently, homologs of MIG-6S typically possess either a tyrosine or a phenylalanine at position Y650 (Fig. 1D’”). Missense allele *mig-6(ev701)* (C848) affects a residue in a lagrin repeat of MIG-6S, where it forms a disulfide bridge with C833 (Fig. 1D’”), perhaps explaining the strong conservation of a cysteine residue at this position. Finally, missense allele *ev700* (harboring substitutions G878E and C879Y) affects two residues located within the lagrin repeats cysteine-rich region of MIG-6S that are also well conserved, with C879 forming a disulfide bridge with C864 (Fig. 1D’”). Thus, mutation at G878 and C879 could perturb the pattern of disulfide bridges within this region. Finally, missense allele *mig-6(sa580)* (G965E) affects a glycine residue located in a lagrin domain, which could disrupt the adjacent cysteine residues involved in interactions with C949 and C973 (Fig. 1D’”). However, *mig-6(sa580)* does not supress *sax-7* neuronal defects (Fig. 1F), highlighting the specific effects of distinct *mig-6* mutations. In sum, these analyses support the idea that the papilin cassette and neighboring lagrin repeats are crucial for the function of MIG-6S in neuronal maintenance.

To experimentally validate which are the most critical regions of the MIG-6S protein in the context of neuronal maintenance, we carried out rescue assays using recombinant versions (Fig. 1E, S4). A recombinant *mig-6S* transgene lacking the sequence that encodes the C-terminal region of the protein, which contains the Kunitz domains, retained rescuing activity in *sax-7(qv30); mig-6(qv33)* double mutant animals with reappearance of neuronal disorganization, similar to the full length *mig-6S(+)* (compare to Fig. 1B). This supports that the Kunitz domains are not essential for MIG-6S function in neuronal maintenance. In contrast, a recombinant *mig-6S* transgene lacking the sequence that encodes the N-terminal region containing the papilin cassette and lagrin repeats failed to rescue the function of *mig-6* in *sax-7(qv30); mig-6(qv33)* double mutants. These results, together with our analyses of a series of mutant alleles (molecular impact in Fig. 1D and functional consequences in Fig. 1F), demonstrate that the papilin cassette and the nearby lagrin repeats are the key domains for MIG-6S functionality in neuronal maintenance.

### *mig-6/papilin* modulates neuronal maintenance post-embryonically and in specific neuronal contexts

Since several *mig-6* mutations suppress the progressive head ganglia disorganization of *sax-7* mutants, we asked whether losing *mig-6* function after the development of ASH/ASI neurons would be sufficient to suppress *sax-7* neuronal maintenance defects. As ASH/ASI neurons complete their development in embryogenesis, we depleted *mig-6* function post-embryonically by feeding *sax-7(qv30)* animals with *mig-6*(RNAi) bacteria starting from the mid/late-L1 larval stage onward. Our results showed that the post-embryonic depletion of *mig-6* function strongly suppressed *sax-7* ASH/ASI neuronal maintenance defects (Fig. 1C), highlighting its post-developmental in maintaining ASH/ASI neuronal architecture.

**Figure 2.**
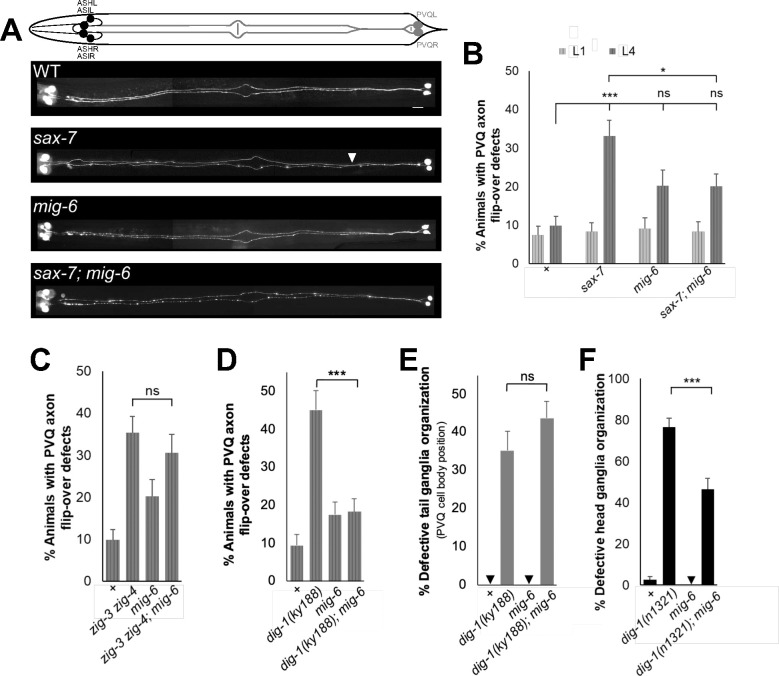
*mig-6/papilin* impacts the maintenance of axon and soma position in specific neuroanatomical and genetic contexts. (**A**) Fluorescence images of 4th larval stage (L4) animals; PVQ neurons visualized using P*sra-6::DsRed2*. In the wild type, the axon of each of two PVQ neurons (L and R) extends ipsilaterally along the ventral nerve cord during embryogenesis and remains on the ipsilateral side throughout life. In sax-7 mutants, whereas PVQ axons develop normally and are positioned like the wild type in early 1st larval stage (L1) animals (not shown), they later become displaced to the opposite side of the ventral nerve cord (L4 shown). Scale bar, 20 μm. (**B,C,D**) Quantification of PVQ axons (L4 animals) shows that *mig-6*(*qv33*) suppresses the axonal defects of *sax-7*(*qv30*) and *dig-1*(*ky188*) mutants, but not of *zig-3*(*tm924*) *zig-4*(*gk34*). (**E, F**) *mig-6*(*qv33*) does not supress PVQ soma displacement in the lumbar ganglia in the tail of *dig-1*(*ky188*) L4 animals, but partially supresses the ASH and ASI soma position relative to the nerve ring in head ganglia of *dig-1*(*n1321*) examined as 2-day-old adult age animals. Error bars are the standard error of the proportion; z-tests were performed.

We next explored if *mig-6*’s impact on neuronal maintenance is context-specific or generalized across the nervous system. *sax-7* mutants are known to exhibit defects in maintaining axon positioning along the ventral nerve cord: axons of bilateral neurons PVQL/R initially develop normally, projecting ipsilaterally during embryogenesis, but later get displaced to the opposite fascicle of the ventral nerve cord in *sax-7* animals ^25^, coinciding with remodeling of the underlying tissue during late first larval stage ^30,49^. We found that *mig-6(qv33)* partially suppresses these *sax-7* axon “flip-over” defects (Fig. 2A, B), indicating that *mig-6* participates in the maintenance of nerve cord organization as well.

The small secreted two-immunoglobulin proteins ZIG-3 and ZIG-4 also function to maintain axon position in the *C. elegans* ventral nerve cord ^30,31,33^, as does the giant basement membrane protein DIG-1, which is also required for ganglia maintenance ^28,29^. To determine if disrupted *mig-6* function could also suppress the defective maintenance of axon position in these other known neuronal maintenance mutants, we generated mutant combinations between *mig-6* and either *zig-3* and *zig-4*, or *dig-1*. We found that *mig-6(qv33)* did not supress the axon flip-over defects in the double mutant of small secreted two-immunoglobulin proteins *zig-3(tm924) zig-4(gk34)* (Fig. 2C), highlighting the specificity of *mig-6* effects. However, *mig-6(qv33)* did suppress axon flip-over in *dig-1(ky188)* mutants (*ky188* is the *dig-1* allele with the most penetrant axonal defects; Fig. 2D). As *dig-1* mutants also exhibit defects in the maintenance of both tail and head neuronal ganglia organization, we examined the impact of *mig-6(qv33)* on *dig-1* ganglia organization maintenance. We found that loss of *mig-6* did not supress defective soma positioning of PVQ neurons in the tail ganglia of *dig-1(ky188)* mutants (*ky188* display progressive and penetrant PVQ soma displacement; Fig. 2E), but did partially suppress head ganglia organization in *dig-1(n1321)* mutants (*n1321* is the most severe *dig-1* allele for head ganglia; Fig. 2F). These results indicate that the role of *mig-6* in neuronal maintenance is specific and context-dependent, varying with the type of neuronal maintenance molecule affected and neuronal structure.

### *mig-6/papilin* functions non-autonomously, together with *mig-17/ADAMTS* to impact neuronal maintenance

**Figure 3.**
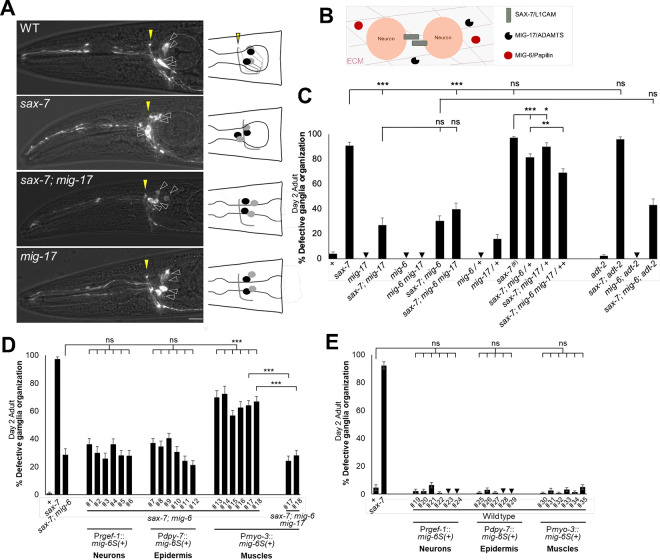
*mig-6*/*papilin* and *mig-17*/ADAMTS function together in neuronal maintenance. (**A**) Fluorescence images of the head region of 2-day-old adults (schematized on the right), with soma and axons of neurons ASH and ASI visualized using P*sra-6::DsRed2* (as in Fig. 1). Scale bar, 10 μm. (**B**) A model proposing functional cooperation between MIG-17/ADAMTS and MIG-6/papilin in the ECM to mediate neuronal maintenance. (**C**) Quantification of ASH and ASI displacement in 2-day adults of wild type, *sax-7(qv30)*, *mig-6(qv33)*, *mig-17(k174)*, and *adt-2(wk156)* single mutants, and their combinations, as homozygous or heterozygous (written as *“mig-6”* or “*mig-6/+*”, respectively). ‘#’ indicates an additional control for *sax-7*, with *dpy-11* in the background, as *dpy-11* was used to distinguish heterozygous animals (see Supplementary Information for full genotypes). Similar to the effect of some *mig-6* mutations, loss of *mig-17* also suppressed *sax-7* neuronal maintenance defects (but not loss of *adt-2*). Simultaneous loss of *mig-6* and *mig-17* did not enhance the suppression of *sax-7* defects. (**D**) Quantification of assays to rescue the function of *mig-6* with tissue-specific expression of *mig-6S(+)*. (**E**) Quantification of control assays showing that *mig-6S(+)* transgenic expression in wild-type animals, including from body wall muscles, does not affect neuronal organization. Error bars are the standard error of the proportion; z-tests.

Given that disrupting the function of the extracellular matrix protein MIG-6/papilin counteracts the progressive neuronal disorganization observed in head ganglia and the nerve cord of *sax-7* and *dig-1* mutants, we hypothesized that the absence of functional MIG-6/papilin protein would affect the ECM surrounding these neuronal structures. In the developing gonad, *mig-6* genetically interacts with *mig-17/ADAMTS*, which encodes a secreted metalloprotease of the ADAMTS family ^40^, thought to remodel the gonadal basement membrane ^21,50–53^. We therefore sought to investigate the functional relationship between *mig-6/papilin* and *mig-17* in neuronal maintenance. We first examined *mig-17(k174)* putative null mutant animals ^54^ (null allele used throughout this study) and found that ASH and ASI neurons are normally positioned with respect to the nerve ring (Fig. 3A,C). *mig-17* mutants exhibit an elongated pharynx ^55^ (Fig. S5), but this did not affect neuronal position with respect to body length. Indeed our data show that there is no difference in the relative positions of the nerve ring, or of ASH and ASI soma, between mutants with normal pharynx length (such as *mig-6(qv33)*) and *mig-17* mutants (Fig. S5), indicating that neuronal positioning is independent from pharynx length. To test if loss of the ECM remodeling molecule *mig-17/ADAMTS* would impact the *sax-7* head ganglia disorganization, we looked at double mutants lacking both *sax-7* and *mig-17*. In double mutants *sax-7; mig-17,* only 27% of animals display head ganglia disorganization, compared to ~90% in *sax-7* single mutants (Fig. 3A,C). This result shows that, similar to loss of *mig-6*, loss of *mig-17* significantly supresses the neuronal maintenance defects in *sax-7* mutants. In contrast, loss of another secreted ADAMTS metalloprotease, ADT-2/ADAMTS, involved in *C. elegans* body size regulation and cuticle structure ^56^, did not supress *sax-7* defects nor enhance their suppression by *mig-6* mutation (Fig. 3C). This highlights the specific role of *mig-17/ADAMTS* in neuronal maintenance.

To then determine whether *mig-6* and *mig-17* function in the same genetic pathway in this context, we constructed a triple *sax-7; mig-6(qv33) mig-17(k174)* mutant strain. We found that the simultaneous loss of *mig-6* and *mig-17* did not further enhance the suppression of neuronal maintenance defects in *sax-7* mutants (Fig. 3C), suggesting that *mig-6* and *mig-17* may function in the same pathway to maintain neuronal architecture. To further probe the notion that *mig-6* and *mig-17* function collaboratively to impact neuronal maintenance, we analyzed the effect of partially losing the function of both genes using double heterozygous animals *mig-6(qv33) mig-17(k174) / mig-6(+) mig-17(+),* abbreviated as *mig-6 mig-17/++*. Single heterozygous animals *mig-17(k174)*/+ only slightly supressed *sax-7* neuronal defects (Fig. 3C). Meanwhile, single heterozygous *mig-6(qv33)/+* mildly suppressed *sax-7* defects; Fig. 3C), consistent with observations that *mig-6* alleles (*ev700*, *ev701* and *k177*) behave in a semi-dominantly due to haploinsufficiency during gonad and PVD neuron development ^40,46^. Notably, in double heterozygous animals *sax-7; mig-6 mig-17/++*, the suppression of *sax-7* defects was significantly greater, with only 69% of animals showing defects, compared to 81% in *sax-7; mig-6/+* animals (Fig. 3C). This result indicates that the two extracellular matrix genes *mig-6/papilin* and *mig-17/ADAMTS* act within the same pathway to influence the long-term maintenance of neuronal organization.

Given that the neurons’ environment controls their maintenance, *mig-6/papilin* may be expected not to function from the neurons themselves. Indeed, extracellular matrix components, including MIG-6, are produced by mesodermal cells, particularly body wall muscles, and the epidermis ^21,40,44,57,58^. We thus generated transgenic *sax-7; mig-6* double mutants animals expressing wild-type *mig-6S* under different tissue-specific promoters: P*rgef-1* for neurons, P*dpy-7* for epidermis (hyp7), and P*myo-3* for mesodermal cells (body wall muscles). Since the loss of *mig-6* suppresses *sax-7* neuronal maintenance defects, restoring *mig-6* function is expected to lead to the reappearance of neuronal disorganization in *sax-7; mig-6* double mutants. We found that expression of *mig-6S(+)* in the neurons or the epidermis did not rescue *mig-6* function. In contrast, expression of *mig-6S(+)* in the body wall muscles in *sax-7; mig-6* double mutant animals robustly rescued the neuronal maintenance defects, with an increase of neuronal defects from 29% up to 72%, depending on the transgenic line (Fig. 3D). This indicates that *mig-6* functions cell non-autonomously from muscles to impact neuronal maintenance. As a control, we expressed these transgenes in wild-type animals and observed no neuronal defects (Fig. 3E), ruling out the possibility that *mig-6S(+)* overexpression by muscles induces artefactual neuronal disorganization. Overall, this confirms that the neuronal defects observed in transgenic animals expressing *mig-6S(+)* in body wall muscles represent *bona fide* rescue of *mig-6* function.

We then investigated whether *mig-17* is necessary for *mig-6*’s function in neuronal maintenance. To test whether the loss of *mig-17* would affect the ability of *mig-6S(+)* transgene to rescue, we generated *sax-7; mig-6 mig-17* triple mutants carrying transgenic lines expressing wild-type *mig-6S(+)* from body wall muscles (lines #17 and 18 used above, Fig. 3D). Unlike the successful rescue of *mig-6* function observed in *sax-7; mig-6* transgenic animals, the loss of *mig-17* prevented the *mig-6S(+)* transgene from rescuing neuronal maintenance defects, as the transgenic triple mutant *sax-7; mig-6 mig-17* animals did not exhibit a reappearance of neuronal defects (Fig. 3D). This may indicate that the normal function of MIG-6/papilin depends on MIG-17/ADAMTS, or alternatively, an excess of MIG-6S cannot compensate for the loss of MIG-17. In conclusion, we find that *mig-6/papilin* acts non-autonomously from muscles to suppress *sax-7* neuronal maintenance defects in a manner that requires the function of *mig-17/ADAMTS*.

### Loss of *mig-6S/papilin* results in increased EMB-9/collagen IV levels that accumulates as extracellular fibrotic-like structures

**Figure 4.**
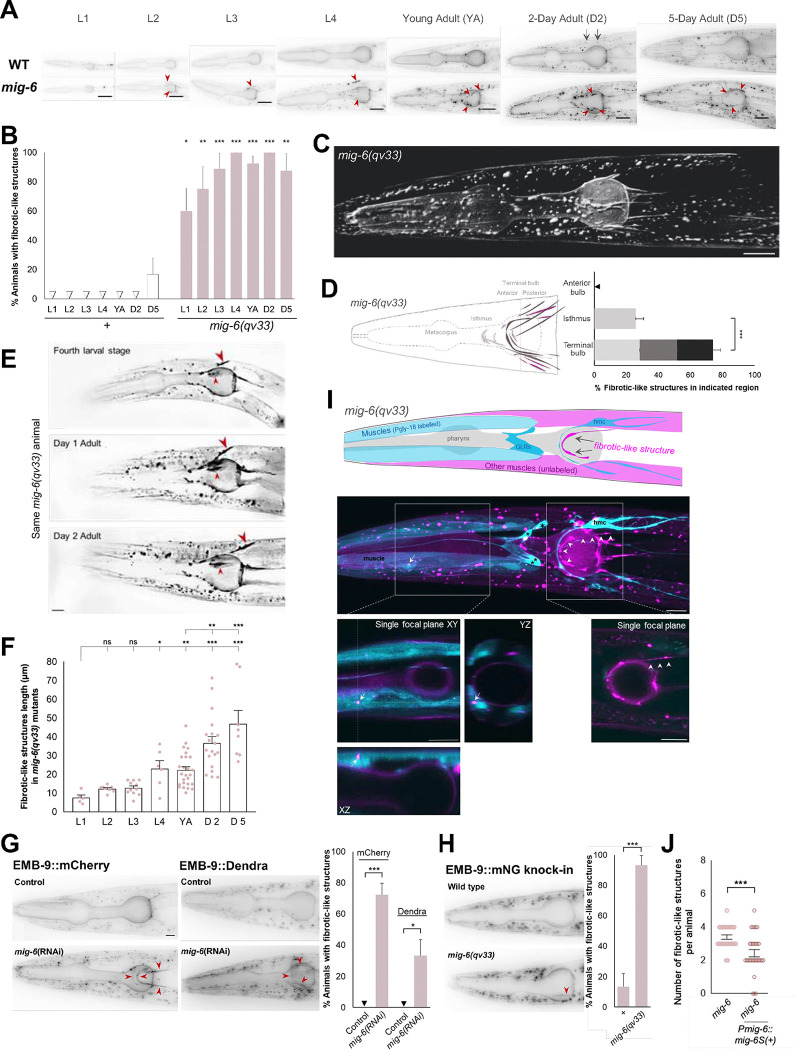
Loss of *mig-6/papilin* function disrupts proper extracellular collagen IV remodeling and causes fibrotic structures. (**A**) Fluorescence images of animals expressing collagen IV reporter P*emb-9*::EMB-9::mCherry at larval stages L1, L2, L3, and L4, and adult ages in control and *mig-6(qv33)* mutants. Thin lines of collagen IV located in the basement membrane of muscle cells are observed in wild-type and *mig-6* animals (indicated by two black arrows), and *mig-6* fibrotic-like structures are indicated by arrowheads. Scale bar, 20 μm. (**B**) Percentage of animals displaying collagen IV fibrotic-like structures at different ages in the wild type and *mig-6(qv33)* mutants expressing EMB-9::mCherry. (**C**) 3D-isosurface renderings (made with Imaris) of collagen IV/EMB-9::mCherry in the head region of a 2-day-old adult *mig-6(qv33)* mutant animal. Scale bar, 20 μm. (**D**) *mig-6* mutants’ collagen IV fibrotic-like structures are typically located in the posterior region of the head, in the general area near the terminal bulb and the posterior of the isthmus of the pharynx. Schematic compilation of fibrotic-like structures’ positions in *mig-6(qv33)* mutants (n>100), and percentage in indicated antero-posterior head regions. These “pharyngeal regions” serve as antero-posterior landmarks only, as these fibrotic-like structures localize in the extracellular space outside the pharynx; also, fibrotic-like structures only occasionally contact the basement membrane of the pharynx (see videos of fluorescence images in Supplementary Information). (**E**) Longitudinal analysis of a *mig-6(qv33)* mutant animal expressing EMB-9::mCherry repeatedly examined as a L4, 1-, and 2-day-old adult shows that the collagen IV fibrotic-like structures (indicated by arrowheads) persist over time. Scale bar, 20 μm. (**F**) Average length of the fibrotic-like structures per *mig-6(qv33)* mutant animal expressing EMB-9::mCherry at different ages. (**G**) Fluorescence images and quantification of animals displaying collagen IV fibrotic-like structures visualized by EMB-9::mCherry or EMB-9::Dendra in 2-day-old adult animals of wild-type animals treated with *mig-6*(RNAi) or empty vector control. Scale bar, 10 μm. (**H**) Collagen IV fibrotic-like structures are also detected in 2-day-old-adult *mig-6* mutants using the endogenous collagen IV reporter *qy24* EMB-9::mNG knock-in. Scale bar, 10 μm. (**I**) Fluorescence image of a young adult *mig-6* mutant, expressing EMB-9::mCherry (magenta), along with *dnIs13* P*gly-18::gfp* (cyan) that labels a subset of head wall muscles, the head mesodermal cell (hmc), and glial cells (GLR, indicated by asterisks); a schematic rendering is shown above. ColIagen IV (EMB-9::mCherry reporter) is observed as intracellular dots or accumulations inside muscle cells (indicated by the arrow on single focal planes on XY, XZ and YZ); and long extracellular fibrotic-like structures (one such structure is pointed to by white arrowheads on main image and on the focal plane on the right panel, which does not coincide with the cytoplasmic extensions of the hmc, nor with other mesodermal cells such as muscles or GLR cells). Magenta circle on single planes is signal from the basement membrane of the pharynx, which also contains collagen IV. (**J**) Rescue of the collagen IV fibrotic-like structures phenotype of *mig-6(qv33)* mutants expressing EMB-9::mCherry. Expression of the *mig-6S(+)* minigene under its own promoter decreases the number of fibrotic-like structures. Error bars are the standard error of the proportion (B, D, G, H) or of the mean (F, J). z-tests (in B, D, G, H), ANOVA in F), or non-parametric Wilcoxon test (in J).

Given that MIG-6/papilin is an extracellular ADAMTS-like protein which interacts functionally with MIG-17/ADAMTS, we hypothesized that the *mig-6* mutation may suppress *sax-7* neuronal maintenance defects by modulating the extracellular environment, including nearby neurons. To directly test whether loss of *mig-6* leads to changes in the extracellular matrix, we analyzed the distribution of a key ECM component, EMB-9/collagen IV α1 (hereafter referred to as ‘EMB-9/collagen IV’), known to genetically interact with *mig-6* during gonadal development ^40^ or to be altered in the gonadal basement membrane in *mig-6*(RNAi)-treated animals ^21^. In *C. elegans*, collagen IV, similar to its vertebrate counterparts, is a heterotrimeric molecule consisting of two EMB-9 (α1-like) chains and one LET-2 (α2-like) chain, previously shown to colocalize ^59–62^. We therefore used a P*emb-9*::EMB-9::mCherry fluorescent reporter ^63^ (gift from David Sherwood) to examine the distribution of collagen IV, focusing particularly on the head region (Fig. 4). In *mig-6(qv33)* mutants, like in the wild type, we observed collagen IV signal along the contour of the pharynx and the surface of body wall muscles (Fig. 4A, Fig. S6), corresponding to the basement membranes of these structures ^21^, as well as in spherical accumulations within muscle cells where collagen IV is produced. However, in *mig-6* mutants, the overall abundance of EMB-9/collagen IV is higher compared to the wild type (Fig. 4A; see Fig. S7 for quantification of collagen IV in entire head region including muscle cells), including inside muscle cells. Moreover, *mig-6* mutants display notable enrichments of collagen IV, often elongated, which we have termed “fibrotic-like structures” (Fig. 4A,B; Fig. S6). These fibrotic-like structures are extremely rare in the wild type and seen only in aging adults (5-day old adults), but are detected as early as the 1st larval stage in *mig-6(qv33)* mutants, and by the 4th larval stage and adulthood, 100% of the *mig-6* mutants present fibrotic collagen IV (Fig. 4A,B). These fibrotic-like structures in *mig-6* mutants are typically located in the posterior region of the head (Fig. 4C,D). Interestingly, our repeated observations of the same *mig-6(qv33)* animals in a longitudinal analysis over several days (Fig. 4E, n=8) show that the *mig-6* mutants’ collagen IV fibrotic-like structures stably persist over time. Measuring the size of these structures at different developmental stages further confirmed that they lengthen in an age-progressive manner (Fig. 4F).

We next studied the localization of the collagen IV fibrotic-like structures that occur in *mig-6* mutants. In *C. elegans*, collagen IV is produced by body wall muscles and other mesodermal cells, including the head mesodermal cell (hmc) and GLR glia ^64,65^. We thus generated a strain of *mig-6(qv33)* mutants carrying both the EMB-9::mCherry reporter and *dnIs13 gly-18p::gfp* ^66^ to simultaneously label collagen IV and anterior head wall muscles, the hmc, and GLR glia, respectively. Confocal microscopy revealed that collagen IV fibrotic-like structures in *mig-6* mutants do not overlap with muscle cells, nor with hmc, nor the GLR glia, and are located extracellularly (Fig. 4I). In addition, spherical collagen IV deposits seen in body wall muscles, appear to be more intense in *mig-6* mutants than in wild type (Fig. 4A; Fig. S6), and these are indeed intracellular (Fig. 4I).

To further support our findings, given that mCherry protein fusions can form aggregates ^67^, we employed an alternative multicopy reporter, EMB-9::Dendra2 ^63^ (gift of David Sherwood). With this reporter we also observed that EMB-9 accumulates as fibrotic-like structures in 33% of animals following *mig-6*(RNAi) knockdown (Fig. 4G). Importantly, using an endogenous CRISPR knock-in reporter for collagen IV ^21^, EMB-9::mNG, 90% of the *mig-6(qv33)* mutants show fibrotic-like structures (Fig. 4H).

Since collagen IV accumulates in *mig-6* mutants, we assessed the stability of EMB-9::mCherry by fluorescence recovery after photobleaching (FRAP), which we performed on muscle and pharyngeal basement membranes that contain collagen IV, present in both the wild type and *mig-6(qv33)* mutants (Fig. S8A). Our FRAP measurements revealed no significant difference between *mig-6* mutants and the wild type, with very limited collagen IV recovery across different time points (Fig. S8B), supporting the notion that loss of *mig-6* does not affect collagen IV short-term dynamics *per se*, which is consistent with its described stable association with the gonadal basement membrane ^21^.

We further strengthened our findings that loss of *mig-6* alters collagen IV levels and distribution by looking at collagen IV in other *mig-6* loss-of-function backgrounds. We generated animals with the *k177* allele of *mig-6* (Y650D mutation in the same domain as *qv33* G664E) carrying EMB-9::mCherry. Similar to *qv33* animals, *k177* mutants exhibit a significant accumulation of EMB-9/collagen IV, with approximately 80% of adult animals displaying fibrotic-like structures (Fig. 6A,B; Fig. S6). Similarly, RNAi-mediated knockdown of *mig-6* resulted in 72% of adult animals exhibiting EMB-9::mCherry fibrotic-like structures (Fig. 4G). In contrast, specifical loss of *mig-6L*, using allele *e1931*, did not alter the collagen IV pattern nor led to fibrotic-like structures (Fig. 6A,B; Fig. S6). These results suggest that *mig-6S*, but not *mig-6L*, is essential for proper extracellular collagen IV organization in the head region. Overall, consistent with the findings of papilin affecting collagen IV in the gonadal basement membrane ^21^, we found that the formation of extracellular collagen IV fibrotic-like structures in the head region is a robust phenotype linked to the loss of *mig-6S/papilin* function.

### *mig-6*/papilin affects the biomechanical properties of the animal’s tissues

**Figure 5.**
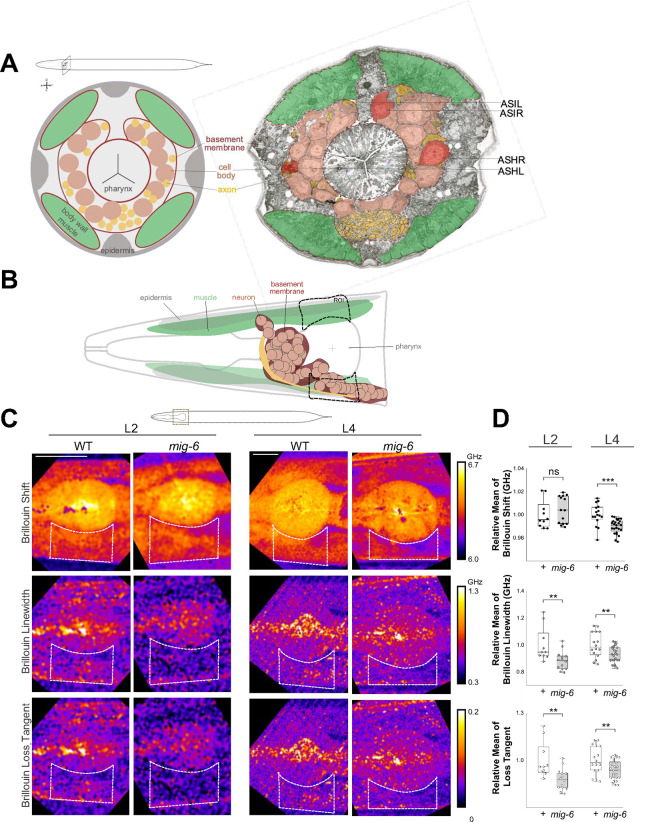
Loss of *mig-6/papilin* alters tissue biomechanical properties. (**A**) Colored electron micrograph of a cross section of an adult wild-type animal in the head region near the location of the ASHL/R and ASIL/R somas (White, Southgate et al. 1986); modified from WormAtlas.org). A schematic of the cross section is provided on the left. Neuronal cell bodies are pseudo-colored in peach, with ASHL/R and ASIR in darker peach; axons in yellow; muscles in green; the location of the basement membranes surrounding body wall muscles, pharynx, and head ganglia have been drawn in to provide context (dark red dashed lines). (**B**) Schematics of the *C. elegans* head region, highlighting prominent cell types in the region imaged by Brillouin microscopy, including neurons of head ganglia, which are surrounded by basement membrane, as well as the ROIs. (**C**) Brillouin microscopy shift, linewidth, and loss tangent images of the head region of L2 and L4 larval stages of wild-type and *mig-6(qv33)* animals. ROIs were drawn flanking the terminal pharyngeal bulb and include several tissues and cell types (muscle, epidermis, neurons, and glia), as well as basement membranes. Scale bar, 20 μm. The color bars indicate absolute values of the shift, linewidth, and loss tangent. (**D**) Quantification of ROIs shows a decrease in elasticity (Brillouin shift), viscosity (Brillouin linewidth), and loss tangent (viscoelasticity) in *mig-6(qv33)* mutants compared to wild type. Mean values of the ROIs were normalized to the wild type (for absolute values, see Fig. S9 and Supplementary Information). Error bars are the standard error of the mean; t-tests were performed.

Our findings demonstrate that disruption of MIG-6/papilin results in a dramatic collagen IV fibrotic phenotype (Fig. 4) and that the progressive neuronal disorganization in head ganglia and the nerve cord of *sax-7* and *dig-1* mutants is counteracted in *mig-6* mutants (Fig. 1 and 2). We therefore hypothesized that the environment surrounding these neuronal structures may be modified in *mig-6(qv33)* mutants in such a way that results in enhanced maintenance of neuronal architecture. To start addressing this possibility, we characterized the biomechanical state of tissues in *mig-6(qv33)* mutants by measuring their viscoelasticity properties using Brillouin microscopy. This label-free imaging technique allows for the assessment of the viscoelastic properties of biological samples through photon–phonon scattering interactions ^68^. The key parameters measured are the Brillouin scattering induced frequency shift and linewidth, which provide information on the high-frequency longitudinal modulus and therefore the elastic and viscous properties of the sample, respectively ^69–71^. We first ensured that the refractive indexes of the head region were similar between *mig-6* and wild-type animals at the examined ages (Fig. S9A), which is an important prerequisite to render the Brillouin microscopy results comparable to one another. Next, using a confocal Brillouin microscope ^72^, we imaged a head region at the level of the studied neuronal cell bodies, which includes other cells such as other neurons, muscles, epidermis, glia, as well as the associated basement membranes/ECM (Fig. 5A,B). Our findings show a significant decrease in Brillouin shift in *mig-6* mutants compared to the wild type at the L4 larval stage (Fig. 5C,D; Fig. S9B), indicating a reduction in tissue elasticity. This change was not detected at the earlier L2 stage, suggesting that the loss of *mig-6* affects tissue elasticity more prominently at later developmental stages. The decreased elasticity was most pronounced in the posterior zone of the region of interest (ROI), which exhibited lower Brillouin elastic contrast (Fig. S9C). Furthermore, Brillouin linewidth measurements revealed that tissue viscosity was also reduced in *mig-6* mutants at both L2 and L4 stages (Fig. 5C,D; Fig. S9B). To assess tissue viscoelasticity in wild-type and *mig-6* mutants, we measured the Brillouin loss tangent parameter ^73^, which showed decreased tissue viscoelasticity in *mig-6* mutants compared to controls at both the L2 and L4 stages (Fig. 5C,D; Fig. S9B). These results demonstrate that MIG-6/papilin is essential for maintaining proper tissue mechanical properties *in vivo* during age-progression. Furthermore, as the altered biomechanical properties of the tissues in the head of *mig-6* mutants are detected earlier than the appearance of ASH/ASI neuronal disorganization in *sax-7* mutants, this suggests that properties of the underlying head ganglia environment play an important role in maintaining neuronal architecture over time.

### *mig-6/papilin* and *mig-17/ADAMTS* function together to regulate extracellular collagen IV

**Figure 6.**
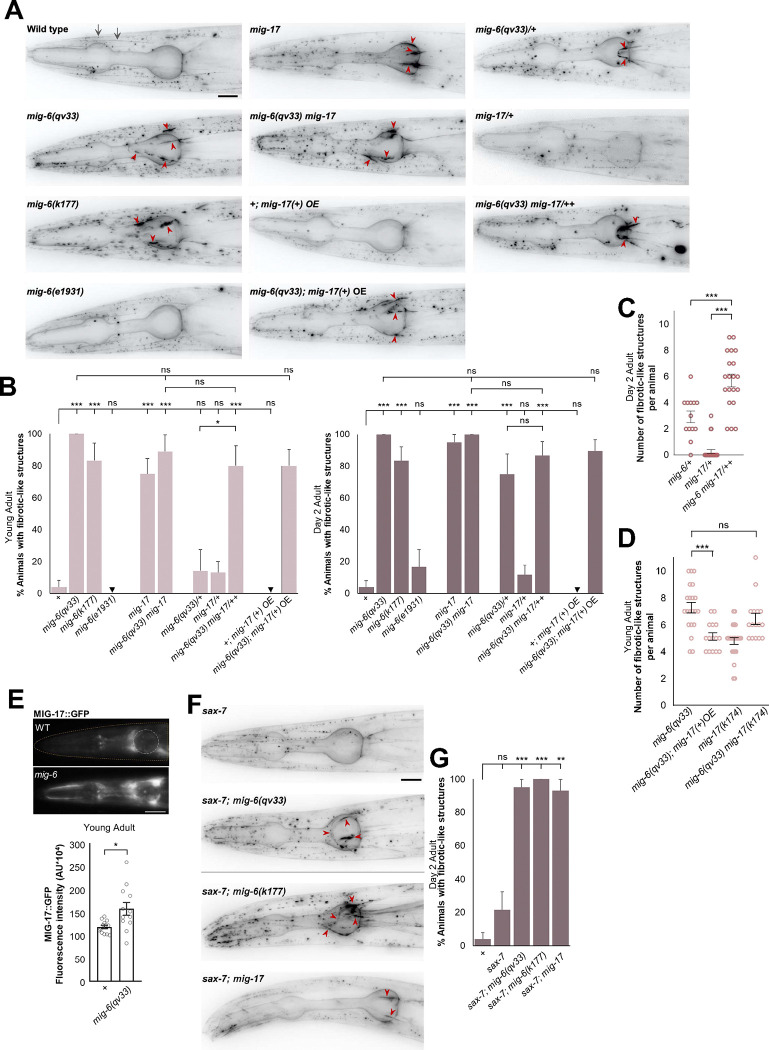
*mig-6/papilin* and *mig-17/ADAMTS* both regulate extracellular collagen IV remodeling. (**A**) Fluorescence images of the head region of 2-day-old adult animals using EMB-9::mCherry. Thin lines of collagen IV located in the basement membrane of muscle cells are observed in all genotypes (indicated by two black arrows); red arrowheads indicate fibrotic-like structures in mutants. Mutant animals for *mig-6*(*qv33* or *k177*) display collagen IV fibrotic-like structures and prominent intracellular accumulations (dots). *mig-17(k174)* mutants also exhibit fibrotic-like structures. In contrast, *mig-6L(e1931)* allele behaves like the wild type. Scale bar, 20 μm. (**B**) Quantification of the percentage of animals displaying collagen IV fibrotic-like structures in young adult and 2-day-old adult animals expressing EMB-9::mCherry. (**C**) Quantification of the number of collagen IV fibrotic-like structures observed per 2-day-old adult heterozygous animal expressing EMB-9::mCherry. (**D**) Quantification of the number of collagen IV fibrotic-like structures observed per young adult animal expressing EMB-9::mCherry. (**E**) Fluorescence images of MIG-17::GFP in the head region of young adults, quantified as fluorescence intensity in head region drawn in orange. Circle, terminal bulb of the pharynx for reference. Scale bar, 24 μm. (**F**) Fluorescence images of the head region of 2-day-old adults in *sax-7(qv30)* null mutant background, using reporter EMB-9::mCherry. Scale bar, 20 μm. (**G**) Quantification of the percentage of 2-day-old adult animals displaying collagen IV fibrotic-like structures. Error bars are the standard error of the proportion (in B, G) or of the mean (in C, D, E). z-tests in B and G, Wilcoxon Mann-Whitney test in C, ANOVA in D, and t-test in E. A.U., arbitrary units. OE, overexpression of wild-type copies. Mutant alleles are only indicated on images and graphs when several alleles of a given gene are used; thus, unless specified otherwise, “*mig-6*” is *mig-6(qv33)* and “*mig-17*” is *mig-17(k174)* throughout this work. Homozygous genotypes are written as “*mig-6*” and heterozygous as “*mig-6/+*”.

Building on the previous finding that *mig-6* genetically interacts with *mig-17/ADAMTS* to suppress *sax-7* neuronal defects (Fig. 3), we investigated whether *mig-6* and *mig-17* also functionally interact in regulating collagen IV distribution. *mig-17(k174)* single mutants exhibit fibrotic-like structures in 75% of young adults and 95% of 2-day old adults (Fig. 6A,B; Fig. S6), similar to the phenotype observed in *mig-6*(*qv33*) and *mig-6*(*k177*) mutants. The simultaneous loss of *mig-6* and *mig-17* in double homozygous *mig-6 mig-17* mutants did not enhance the collagen IV fibrotic-like structures compared to single mutants, neither in penetrance (Fig. 6B), nor in expressivity (Fig. 6D), consistent with the notion that the extracellular matrix genes *mig-6* and *mig-17* function within the same pathway to regulate collagen IV distribution.

To reinforce this conclusion, we assessed the effect of simultaneously losing a single functional copy of each gene, which can provide insights into genetic interactions (avoiding the ceiling effect, as heterozygous animals are much less penetrant for this phenotype). In *mig-6(qv33)/+* single heterozygotes, we observed collagen IV fibrotic like-structures in approximately 10% of young adults, increasing to 75% of 2-day-old adults (Fig. 6A,B; Fig. S6), indicating a semi-dominant effect of *mig-6* also for this phenotype. In *mig-17(k174)/+* single heterozygotes, only around 10% of animals displayed fibrotic like-structures at both ages. Remarkably, in young adult double heterozygotes *mig-6 mig-17/++*, as many as 80% exhibited collagen IV fibrotic-like structures (increasing to 85% in 2-day-old adults; Fig. 6A,B; Fig. S6). Also, the number of fibrotic-like structures per animal strikingly increases in the double heterozygous *mig-6 mig-17/++* animals compared to single heterozygous (Fig. 6C). Together, these results firmly establish that *mig-6* and *mig-17* functionally interact to regulate extracellular collagen IV.

MIG-17/ADAMTS has been hypothesized to function as a metalloprotease that degrades collagen IV ^74,75^. We thus asked whether overexpression of wild-type copies of *mig-17(+)* impacts the collagen IV phenotype of *mig-6* mutants. For this we used functional transgene P*mig-17::mig-17(+)::gfp* (Nishiwaki et al, 2000; Jafari et al, 2010) (Fig. 6A and Fig. S6), combining the integrated transgene (*evIs213*, gift of Joe Culotti) with *mig-6(qv33)*. We found that while the percentage of *mig-6* animals displaying fibrotic-like structures was unchanged (Fig. 6B), the number of fibrotic-like structures per young adult animal significantly decreases in *mig-6* mutants overexpressing the *mig-17(+)* transgene (Fig. 6D). This result indicates that the collagen IV defects of *mig-6* mutants can be partially offset by *mig-17/ADAMTS* overexpression.

As *mig-6* mutants display a fibrotic collagen IV phenotype, and the overexpression of *mig-17(+)* partially suppresses this defect (Fig. 6D), we wondered whether levels of MIG-17/ADAMTS may be lowered in *mig-6* mutants. To test this idea, we examined the distribution of MIG-17/ADAMTS in *mig-6(qv33)* mutants using P*mig-17::*MIG-17::GFP (*evIs213*). We found that instead of being decreased, MIG-17 is in fact upregulated in *mig-6* mutants (Fig. 6E), with new enrichments in different head regions compared to wild type. Given that increased *mig-17(+)* levels can at least partially suppress *mig-6* mutants (Fig. 6D), and that MIG-17 levels are elevated in *mig-6* mutants (Fig. 6E), together these results suggest that MIG-17/ADAMTS’s function requires functional MIG-6/papilin to robustly ensure normal collagen IV distribution.

### Suppression of *sax-7* neuronal maintenance defects upon loss of *mig-6/papilin* and *mig-17/ADAMTS* depends on collagen IV levels and cross-linking

Because *mig-6* and *mig-17* cooperate to regulate extracellular collagen IV (Fig. 6A–D) and maintain neuronal organization in *sax-7* mutants (Fig. 3A,B,C), we asked if the suppression of *sax-7* neuronal maintenance defects upon loss of *mig-6* or *mig-17* is linked to changes in collagen IV patterning in the ECM. We assessed the state of collagen IV distribution in the *sax-7* mutant background and found that *sax-7* single mutants behave like the wild type in this regard (Fig. 6F,G; S6). Also, double mutants *sax-7; mig-6(qv33)* and *sax-7; mig-6(k177)*, as well as *sax-7; mig-17(k174)* displayed a fibrotic-like structure phenotype like that of the respective *mig-6* or *mig-17* single mutants (Fig. 6F,G; S6). That *sax-7; mig-6* and *sax-7; mig-17* double mutants exhibit modified collagen IV pattern (Fig. 6F,G) and maintain organized head ganglia (Fig. 3C) is consistent with the notion that the state of the ECM plays a role in maintaining neuronal architecture. In line with this, *mig-6L*-specific allele *e1931* does not affect the pattern of collagen IV (normal levels and no fibrotic structures; Fig. 6A,B; S6) and does not suppress the neuronal maintenance defects of *sax-7* mutants (Fig. 1F).

**Figure 7.**
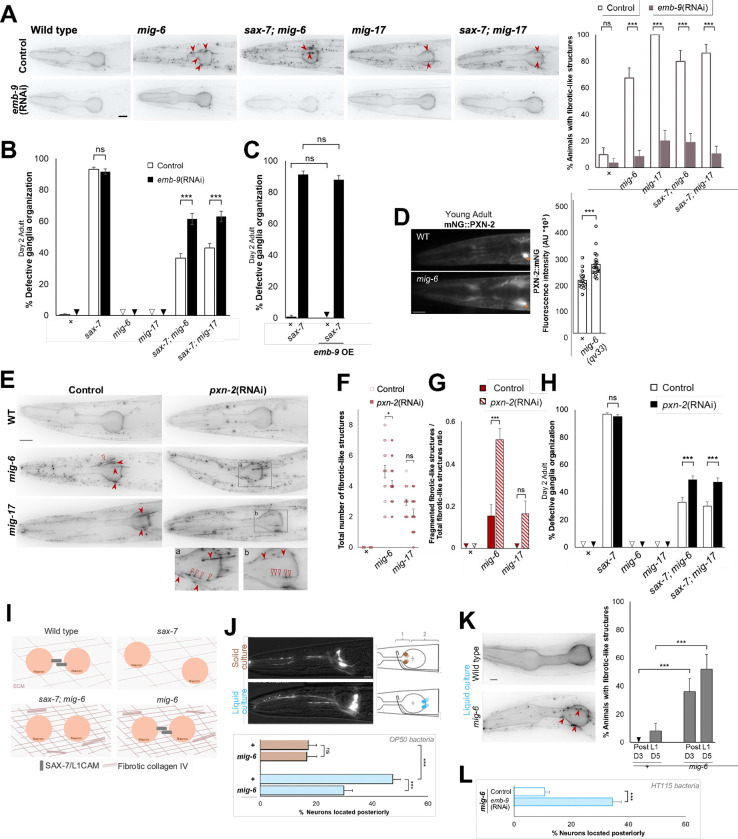
Extracellular collagen IV networks are key for *mig-6/papilin*’s role in neuronal maintenance, including under conditions of increased stress. (**A**) Fluorescence images of the head region of 2-day-old adults using alleles *mig-6(qv33), sax-7(qv30)* and *mig-17(k174)*, expressing EMB-9::mCherry, subjected to control (empty vector) or *emb-9*(RNAi) from the L1 stage; fibrotic-like structures indicated by red arrowheads. Quantification of the percentage of animals with fibrotic-like structures (see Fig. S10A for quantification of collagen IV levels). (**B**) Knockdown of collagen IV by *emb-9*(RNAi) reverses the suppression of *sax-7(qv30)* neuronal defects by loss of *mig-6(qv33)* or *mig-*17*(k174)*. Quantification of neurons ASH and ASI displacement (as in Fig. 1) in 2-day-old adults subjected to control (empty vector) or *emb-9*(RNAi) since the L1 stage. (**C**) Increase of *emb-9*/collagen IV levels alone (overexpression using *qyIs46* multicopy integrated transgene P*emb-9*::EMB-9::mCherry) is not sufficient to suppress *sax-7(qv30)* neuronal defects. (**D**) Fluorescence images of mNG::PXN-2 in wild-type and *mig-6(qv33)*, scale bar, 20 μm; and quantification (see Fig. S12 for ROI and additional images). A.U., arbitrary units. (**E**) Knockdown of collagen IV crosslinking enzyme PXN-2/peroxidasin by *pxn-2*(RNAi) reduces the number of fibrotic-like structures and alters their state. Fluorescence images of collagen IV (*qyIs46* EMB-9::mCherry) in 2-day-old adults of wild type and mutants *mig-6(qv33)* and *mig-17(k174)*, subjected to control (empty vector) or *pxn-2*(RNAi) from the L1 stage. Insets (a and b) show detail of fibrotic-like structures that appear continuous (arrowheads) or fragmented (empty arrowheads). Scale bars, 20 μm. (**F**) Quantification of the number of fibrotic-like structures per animal expressing EMB-9::mCherry in wild type, *mig-6(qv33)*, and *mig-17(k174)* mutant animals in control (empty vector) and *pxn-2*(RNAi) conditions. Both fragmented and non-fragmented fibrotic-like structures are included in this quantification of fibrotic like-structures number. (**G**) Quantification of the ratio of fragmented to total fibrotic-like structures per animal in wild type and mutants *mig-6(qv33)* or *mig-17(k174)* expressing EMB-9::mCherry in control (empty vector) and *pxn-2*(RNAi)-treated conditions. (**H**) The suppression of *sax-7(qv30)* neuronal defects by mutations *mig-6(qv33)* or *mig-17(k174)* is reversed by deficient collagen IV crosslinking in *pxn-2*(RNAi)-treated animals. Quantification of the displacement of neurons ASH and ASI in 2-day old adult animals subjected to control (empty vector) or *pxn-2*(RNAi). (**I**) Summary of the relationship between ECM remodeling and neuronal maintenance in the context of *sax-7* and *mig-6* mutants. Loss of *mig-6* compensates for loss of neural adhesion molecule SAX-7 through extracellular remodeling, ensuring the maintenance of neuronal organization. (**J**) Fluorescence images of the head region of wild-type adults fed regular *E. coli* OP50 and imaged in 2-day adults when grown on solid media, or at the equivalent age of 5 days-post L1 hatching when grown in liquid. Neurons ASH and ASI were visualized using reporter P*sra-6::DsRed2*; drawings illustrate their soma position in wild-type animals grown on solid (brown), or in liquid (blue, notice the posterior placement of neurons when worms swam in liquid conditions). Scale bar, 10 μm. Quantification of neuronal placement in wild-type and *mig-6(qv33)* adults, when grown in solid (2-day adults) or liquid conditions (5 days-post L1 hatching). In each animal, neurons were considered posteriorly displaced when at least one of the four soma was in area 2 (posterior to the pharyngeal grinder, indicated by the cross). (**K**) Fluorescence images of collagen IV (EMB-9::mCherry) in wild type and *mig-6(qv33)* adults at 5 days-post L1 hatching, grown in liquid since L1 hatching. Scale bar, 10 μm. Quantification of the percentage of animals displaying collagen IV fibrotic-like structures in wild-type and *mig-6(qv33)* adults grown in liquid conditions since hatching (examined at 5 days-post L1 hatching). (**L**) Animals were grown in liquid conditions since hatching while being subjected to RNAi treatment; animals were fed *E. coli* HT115 bacteria harboring the empty vector (control RNAi) or the *emb-9*(RNAi) vector to deplete collagen IV. Quantification of neuronal placement (as in C) in wild-type and *mig-6(qv33)* adults, examined at 5 days-post L1 hatching. Collagen IV depletion prevents the stabilizing effect of *mig-6* mutation. Error bars are the standard error of the proportion (z-tests in A-C, H, J-L) or of the mean (t-test in D, Wilcoxon Mann-Whitney test in F and G).

We then investigated whether collagen IV levels and distribution contribute to the maintained neuronal organization of double mutants *sax-7; mig-6* and *sax-7; mig-17*. We depleted *emb-9/collagen IV* by RNAi treatment of animals from the 1st larval stage and examined head ganglia organization in adults. This *emb-9*(RNAi) knockdown effectively depleted EMB-9/collagen IV levels (Fig. 7A, Fig. S10A), and importantly, did not affect neuronal organization in wild-type animals, nor in single mutants. In contrast, depleting collagen IV reversed the suppression of *sax-7* neuronal defects by *mig-6* or *mig-17* mutation. Indeed, double mutant animals *sax-7; mig-6* and *sax-7; mig-17* showed increased neuronal disorganization upon *emb-9*(RNAi) (Fig. 7B). This result indicates that collagen IV levels are key for the suppression of *sax-7* neuronal defects by the loss of *mig-6* or *mig-17*. We then tested whether a higher level of collagen IV could mimic the effect of *mig-6* loss of function in suppressing *sax-7* neuronal maintenance defects. We found that *sax-7* animals overexpressing transgene *emb-9(+)* (in *sax-7; qyIs46* animals carrying the multicopy transgene P*emb-9::*EMB-9::mCherry) did not show suppression of neuronal maintenance defects (Fig. 7C). Thus, while sustained levels of collagen IV are required to suppress *sax-7* neuronal defects, elevated collagen IV level *per se* is insufficient to ensure neuronal maintenance.

We therefore investigated whether collagen IV organization plays a role in neuronal maintenance as well. Collagen IV molecules form complex crosslinked networks involving dimerization through their NC1 domain ^65,76,77^. The extracellular enzyme peroxidasin catalyzes sulfilimine S=N bonds between collagen IV NC1 domains ^76,78^, which are essential for collagen IV networks and basement membrane integrity ^79–82^. The *C. elegans* genome encodes two peroxidasins, of which PXN-2/peroxidasin is known for its effects on the ECM and genetic interactions with collagen IV genes ^83^. We examined the pattern of PXN-2/peroxidasin using a knock-in fluorescent reporter mNeonGreen::PXN-2 (driven under the *pxn-2* promoter ^21^, and found that the expression of PXN-2/peroxidasin is upregulated in *mig-6(qv33)* mutants compared to wild type (Fig. 7D), suggesting that *mig-6* is implicated in the regulation of peroxidasin 2 levels in the ECM. Knockdown of *pxn-2/peroxidasin* by RNAi (from the 1st larval stage) did not significantly lower the penetrance of fibrotic-like structures (Fig. S10B), but significantly reduced the number of fibrotic-like structures (Fig. 7E,F), and led to a striking increase of fragmented fibrotic collagen IV (Fig. 7E,G). Importantly, *pxn-2*(RNAi) knockdown reverses the suppression of *sax-7* mutants’ neuronal defects by loss of *mig-6* or *mig-17* (Fig. 7H). Together, these results highlight that the function of MIG-6/papilin and MIG-17/ADAMTS in neuronal maintenance is dependent on collagen IV and its crosslinking by the peroxidasin enzyme. Further, they support the notion that the elevated levels of crosslinked collagen IV in the ECM of *mig-6* mutants contribute to stabilizing neuronal position and maintaining neuronal architecture in *sax-7* mutants (Fig. 7I).

### Loss of *mig-6*/papilin counteracts neuronal disorganization induced by increased mechanical stress

Since loss of *mig-6* function positively impacts the maintenance of neuronal organization in animals lacking the cell adhesion molecule SAX-7/L1CAM, we hypothesized that it may also support neuronal architecture in otherwise wild-type animals that experience increased internal mechanical stress. We used the distinct locomotion patterns of *C. elegans* to probe this question. In liquid media, *C. elegans* swims, whereas on solid media, it crawls. Swimming and crawling differ in neuromuscular activity and speed, with swimming being faster ^84,85^. The associated exerted forces are also different: when the worm crawls on solid media, the forces exerted on the worm’s cuticle are higher than when swimming in liquid ^86–88^. By contrast, swimming worms perform many more body bends, with more numerous body wall muscles contractions, which is expected to result in higher mechanical stress on internally located neurons (e.g., in head ganglia) compared to the slower movements of worms crawling on solid media. We therefore subjected worms to continuous swimming in liquid culture from the time of hatching to adulthood, and assessed the position of sensory neurons ASH and ASI. Compared to the neurons in animals grown on solid medium, neuronal position in wild-type animals changed significantly when grown in liquid medium for 5 days, exhibiting a significant posterior displacement (Fig. 7J). However, *mig-6(qv33)* mutant animals raised in liquid medium showed a significant decrease in neuronal displacement (Fig. 7J), indicating that loss of *mig-6/papilin* function results in enhanced maintenance of neuronal organization upon high internal mechanical stress.

Because collagen IV is required for the stabilizing effect of *sax-7* mutants neuronal organization by loss of *mig-6* function, we examined the state of collagen IV in swimming animals. We observed that *mig-6* mutants display an altered collagen IV pattern (similar to that of *mig-6* mutants grown on solid medium), with the presence of fibrotic-like structures, increasing in penetrance from day 3 to day 5 post L1 hatching (Fig. 7K). We then asked if collagen IV was required for the neuronal protective effect conferred by the *mig-6* mutation in otherwise wild-type animals when grown in liquid. Depleting collagen IV by *emb-9*(RNAi) of swimming animals significantly weakened the *mig-6*-mediated stabilizing effect of neuronal organization of ASH and ASI neurons (Fig. 7L). This result indicates that collagen IV is critical in the neuronal protective mechanism involving *mig-6/papilin* in conditions of increased mechanical stress.

## DISCUSSION

**Figure 8.**
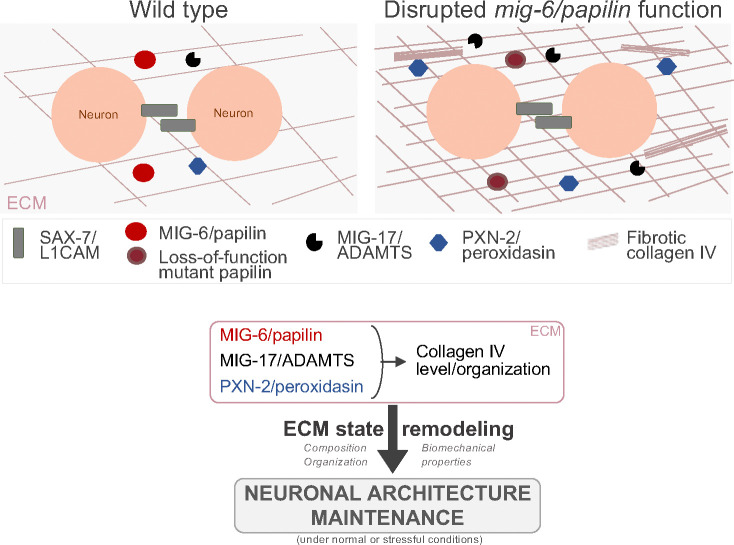
A model for the role of MIG-6S/papilin in collagen IV remodeling and neuronal maintenance. Summary of the cooperative functions of conserved ECM regulators MIG-6S/papilin, MIG-17/ADAMTS, and PXN-2/peroxidasin in the modulation of collagen IV levels and organization, impacting the long-term maintenance of neuronal architecture.

Neuronal architecture established embryonically must persist throughout life to ensure nervous system function. However, the mechanisms sustaining neuronal organization over the long term remains poorly understood. This work uncovers a novel mechanism where ECM dynamics plays a critical role in maintaining neuronal architecture. Through a multidisciplinary approach, integrating forward genetic screening, incisive molecular genetic analysis, structural molecular predictions, quantitative live imaging, and measurement of biomechanical properties by Brillouin microscopy, we have identified the evolutionarily conserved extracellular matrix protein MIG-6/papilin as a key regulator of the long-term maintenance of the neuronal architecture. We show that MIG-6/papilin impacts neuronal maintenance by modulating the animal’s tissues biomechanical properties and remodeling the extracellular network of collagen IV, which is a major component of the basement membranes, including those surrounding neuronal assemblies. We also find that ECM metalloproteinase MIG-17/ADAMTS is important for sustaining neuronal architecture, and functionally cooperates with MIG-6/papilin in ECM remodeling in order to enable the long-term stability of neuronal architecture. Both the abundance and the cross-linking of collagen IV networks are essential for the MIG-6/papilin-remodeled ECM state that enables the maintenance of neuronal structures. Thus, this work reveals a previously unknown mechanism by which ECM remodeling enables the preservation of neuronal architecture (Fig. 8), in the face of age-progressive stresses, to preserve continuous neural function.

MIG-6/papilin is expressed throughout life, from embryogenesis to adulthood ^21,36,40,57,58^, in dynamic patterns that may reflect its requirements at the different life stages. Papilin indeed plays key developmental roles: the lack of papilin results in embryonic lethality in null mutant worms and in RNAi-depleted flies ^36,40^. Papilin is also required for organogenesis in flies ^36^, distal tip cell migration of the developing *C. elegans* gonad ^40^, as well as for enlargement of the gonad and the pharynx during *C. elegans*’ growth ^21,44^. A role for papilin in the nervous system has remained largely unexplored. There is one study in *C. elegans* showing that papilin participates axon guidance of the neuron ALA, which impacts primary dendrite development of the neuron PVD ^46^. In *Drosophila*, a recent report on a screen for regulators of central nervous system morphology mentions a papilin mutant found to have a misshapen central nervous system ^45^, which awaits further analysis. In our study, we isolated the mutation *mig-6(qv33)* in a screen for animals that suppress the age-progressive disorganization of *sax-7*/L1CAM mutants. Similar to other *mig-6* mutations, *mig-6(qv33)* mutant animals have gonad abnormalities, but otherwise display normal body morphology (i.e., normal musculature, pharynx, epidermis, and overall neuronal architecture). We uncovered a post-developmental role of *mig-6* in maintaining the positioning of neurons ASH and ASI, since depletion of *mig-6* function by RNAi treatment initiated from the first larval stage onwards suppressed the ASH and ASI neurons’ defects that progressively accumulate in *sax-7* null mutants, after having normally developed during embryogenesis.

*mig-6/papilin* encodes two isoforms, and our allelic series analysis and rescue assays demonstrate that the short isoform, MIG-6S, is active in neuronal maintenance, while MIG-6L is dispensable in this context. Isoform-specific roles of *mig-6* have been previously described in the gonad development ^40^ and ALA-PVD neuronal patterning ^46^. MIG-6S belongs to the ADAMTS-like ECM protein family, and consists of several domains, including the papilin cassette with its TSP1 repeats and the ADAMTS spacer, followed by numerous cysteine-rich lagrin repeats, and Kunitz domains. Our genetic and protein-domain analyses, combined with molecular predictions, point to the papilin cassette and the closest lagrin repeats as being critical for MIG-6S function in neuronal maintenance. Indeed, disruption of *mig-6S* function by *mig-6(qv33)* or other mutations affecting the papilin cassette/adjacent lagrin repeats suppressed the age-progressive neuronal disorganization of *sax-7/L1CAM* mutants, but *mig-6(sa580)* that affects a different lagrin repeat did not impact the neuronal maintenance. Allele-specific effects of mutations affecting this region of the protein have also been described in the context of pharynx growth, where mutants *mig-6(sa580)* do display a twisted pharynx, but the mutations *mig-6(k177, ev700,* or *ev701)*, which like *mig-6(qv33)* affect residues in more N-terminally located TSP1 domains or lagrin repeats, do not affect pharynx development ^44^. These allele-specific defects likely reflects the complexity of interactions of this multidomain MIG-6/papilin. Interestingly, *mig-6(qv33)* is a semi-dominant allele in neuronal maintenance, indicating that a minimum level of MIG-6/Papilin is required for proper function in this context. The *mig-6* locus was similarly described as haploinsufficient in gonad and ALA-PVD neuron development ^40,46^.

In both *C. elegans* and *Drosophila*, papilin is expressed by cells in charge of producing the ECM/basement membrane components, such as body wall muscles and epidermis in the worm, and hemocytes in flies. Indeed, we found that expression of MIG-6S from body wall muscles rescued its function in neuronal maintenance. Interestingly, MIG-6S/papilin itself localizes to the basement membrane of several organs, including the gonad, the pharynx, the intestine ^21,40^, as well as nerve structures (e.g., nerve tracts ^46^) in *C. elegans* larvae and adults. Similarly, in *Drosophila*, papilin localizes to the basement membranes, including those enveloping the central and peripheral nervous system of both larvae and adults ^36^. Interestingly, publicly available data shows that papilin is also expressed in central nervous system of adult mice (Allen Atlas, https://portal.brain-map.org/), suggesting papilin may function in the adult mammalian brain as well.

How might the basement membrane protein MIG-6/papilin regulate neuronal maintenance? The extent to which ECM remodeling determines the long-term preservation of the neuronal architecture laid out earlier in development is only beginning to be probed. We report that the role of the extracellular papilin MIG-6 and the ADAMTS protease MIG-17 in maintaining neuronal organization in *C. elegans* is through their cooperative function in regulating collagen IV. Papilin is a component of basement membranes but appears to have essential roles in their assembly or maintenance, since all of the *mig-6* mutants analyzed and *mig-6*(RNAi) treated animals display continuous basement membranes surrounding the pharynx, body wall muscles and the gonad, similar to wild type (this study, ^21^). Rather, papilin appears to affect specifically the remodeling of basement membranes, as disruption of MIG-6/papilin results in a dramatic build-up of extracellular collagen IV, a major component of ECM/basement membranes, indicating that MIG-6/papilin regulates collagen IV removal and distribution in the ECM. In *mig-6S* mutants and *mig-6*(RNAi) depleted animals, collagen IV accumulation is visible during larval stages and increases with age (both in terms of penetrance and of the extent of collagen build-up per animal), including within given individual animals as shown by our longitudinal analysis. Moreover, we observed an increase in intracellular collagen IV levels in body wall muscles, which produce both ECM components and MIG-6/papilin, suggesting that MIG-6/papilin may also impact collagen IV synthesis or degradation. RNAi-depletion of *mig-6* also results in collagen IV accumulation in the gonadal basement membrane ^21^.

MIG-6/papilin is an ADAMTS-like protein, sharing structural similarities with ADAMTS secreted ECM metalloproteinases, but lacking a catalytic domain. Its biochemical function is unclear, but *Drosophila* papilin can bind a procollagen N-proteinase ADAMTS *in vitro*, inhibiting its activity non-competitively, without directly interfering with the enzyme’s catalytic site ^36^. The papilin cassette alone could also inhibit the procollagen N-proteinase. The ‘papilin cassette’ in the papilin ADAMTS-like proteins is important for binding to ECM ^89^, and papilin domains often interact with ADAMTS proteases also containing a papilin cassette ^36^, further supporting a regulatory role of this key region in ECM remodeling. MIG-17 is an atypical ADAMTS enzyme as it lacks TSP1 domains and thus a papilin cassette; yet MIG-17 is classified as belonging to the ADAMTS family based on the other significant structural similarities to ADAMTS proteins ^54,90^. Our genetic and molecular analyses revealed that ADAMTS-like protein MIG-6/papilin and ADAMTS metalloproteinase MIG-17 function within the same pathway to regulate ECM remodelling. Indeed, (i) the loss of *mig-17* mirrors the *mig-6S* loss-of-function phenotype of extracellular collagen IV build up; (ii) the simultaneous loss of both genes in double homozygous mutant animals does not enhance the collagen IV fibrotic phenotype, and (iii) loosing half of the function of both genes in double heterozygous animals strongly enhances the defects. Although they function together to promote removal of extracellular collagen IV, *mig-17* and *mig-6S* mutants do have phenotypic differences, notably relating to pharynx development; also, *mig-6S* mutants display a higher level of intracellular collagen IV in muscle cells, while *mig-17* mutants do not. As a note, another genetic lesion of *mig-17*, *ola226*, was reported to have extracellular collagen IV accumulation in the head region ^51^. In sum, our data support the notion that MIG-6/papilin and MIG-17/ADAMTS functionally cooperate to regulate collagen IV remodeling. MIG-17/ADAMTS has been hypothesized to possess proteolytic activity toward collagen IV, based on studies both in *C. elegans* and *Drosophila* ^74,75,91^. It is thus conceivable that the build-up of collagen IV in the ECM of *mig-6* mutants could result from MIG-17 being inhibited or less efficient, especially since we found that MIG-17 levels are increased in the head region of *mig-6* mutant animals. We directly tested this by overexpression of functional MIG-17/ADAMTS, which did not reverse the collagen IV fibrotic phenotype in *mig-6* mutants, suggesting that MIG-17/ADAMTS is in its active form in *mig-6S* mutants yet unable to degrade collagen IV, perhaps due to its high degree of crosslinking ^92–94^. In this scenario, MIG-6 regulates the level and activity of an ADAMTS through its impact on the ECM state.

Interestingly, we show that MIG-6/papilin influences the levels and distribution of MIG-17/ADAMTS and of the extracellular collagen IV crosslinking enzyme PXN-2/peroxidasin in the vicinity of the affected neuronal structures. Other studies have also documented that MIG-6/papilin affects the distribution of MIG-17 in the basement membrane of the developing gonad ^40^, and *mig-6* depletion by RNAi increased the levels of the ADAMTS proteinases MIG-17 and GON-1, and of PXN-2/peroxidasin-2 in the gonadal basement membrane ^21^. Whether this involves a physical interaction (direct or indirect) between MIG-6/papilin and these proteins is to be determined. Regardless, these observations together suggest that papilin might play a broad role in collagen IV/ECM remodeling. Importantly, we show that loss of *mig-6S* or loss of *mig-17* profoundly suppresses the neuronal maintenance defects that occur in *sax-7/L1CAM* mutants. Furthermore, losing the function of both MIG-6 and MIG-17 in homozygous double mutant animals did not enhance the suppression of the neuronal maintenance defects of *sax-7* mutants, and loss of one copy of each gene in double heterozygous animals significantly enhanced the suppression compared to each heterozygous single mutant. These observations are consistent with the notion that MIG-6 and MIG-17 function in the same pathway to impact the maintenance of neuronal architecture. Evidence for a functional relationship between MIG-6 and MIG-17 exists also in the context of the *C. elegans* developing gonad, where *mig-6* and *mig-17* genetically interact ^40^. Importantly, MIG-17 is not involved in ALA-PVD neurons patterning, indicating that MIG-6/Papilin operates through distinct mechanisms depending on the biological context, which is consistent with the specificity of defects displayed by different alleles affecting *mig-6S*, possibly interacting with distinct functional partners through distinct regions of this multidomain protein.

At the level of multicellular neuronal structures, such as ganglia or nerve cords, a delicate balance must exist between ECM stability, which preserves the architecture of the existing neuronal structures, and ECM remodeling, which accommodates growth of the neuronal structures during post-natal life, as well as adapting to shape changes that accompany the animal’s movements. The shared fibrotic collagen IV phenotype between *mig-6S* and *mig-17* mutants suggests that the altered state of collagen IV in these two mutants contributes to their ability to sustain neuronal architecture in *sax-7* mutants. An excess of crosslinked collagen IV may reinforce the integrity of the basement membrane, thereby supporting the maintenance of neuronal organization. We favor a model in which enhanced basement membrane integrity leads to maintained neuronal architecture for several reasons. First, the *mig-6* and *mig-17* mutations that do suppress *sax-7*-neuronal disorganization display a dramatic accumulation of extracellular collagen IV. Second, both collagen IV abundance and its crosslinking are required for neuronal maintenance, as reducing collagen IV by *emb-9*(RNAi) significantly reversed the stabilizing effect brought about loss of MIG-6S or of MIG-17, as does reducing the crosslinking of collagen IV by RNAi knockdown of PXN-2/peroxidasin ^95^. Collagen IV was also key in the role of MIG-6/papilin in modulating the response of neuronal architecture to heightened mechanical stress, as loss of *mig-6* was protective of head ganglia organization in animals subjected to swimming which leads to increased mechanical stress on the nervous system, due to the constant and rapid swimming muscle contractions was also dependent on collagen IV levels. Collectively, these findings underscore that extracellular collagen IV networks are key in neuronal maintenance. The fibrotic-like structures displayed by *mig-6* and *mig-17* mutants are unlikely directly involved in stabilizing neuronal architecture; rather, these fibrotic accumulations are the most obvious manifestations of dysregulated ECM remodelling, which also affects the basement membranes surrounding neuronal structures in *mig-6* and *mig-17* mutants.

Collagen IV, thanks to its unique ability to form intermolecular covalent bonds, provides the basement membrane with the capacity to withstand mechanical stress ^76,96^. Thus, we characterized the mechanical properties that result from the loss of functional MIG-6/papilin, more specifically, by analyzing the high-frequency longitudinal modulus of tissues, using Brillouin microscopy ^71^. We imaged an area neighboring the neurons under study, and compared mechanical properties of *mig-6* mutants and wild-type animals at two ages, earlier in larval life, and just before becoming adults. The area imaged, located in the posterior region of the animal’s head, comprises several cell types, including neurons, muscles, glia, pharynx, and their ECM. While the contribution of each individual adjacent cell and of the local ECMs to the measured mechanical proprieties cannot be reliably discriminated in intact animals with the current resolution of the Brillouin microscope, ECM including basement membranes, is known to exert a key influence on tissue biomechanics ^97–99^. Thus, tissue viscoelastic properties are significantly determined by the ECM ^97,99^. Our Brillouin spectral analysis revealed that loss of MIG-6/papilin results in altered biomechanical properties in the head region which houses the neuronal ganglia we primarily analyze. Collectively, the imaged tissues and associated ECMs in *mig-6* mutants have reduced viscosity and elasticity, indicating impaired viscoelastic properties. Importantly, cellular viscoelasticity is a regulator of cell behavior, associated with both physiological and pathological states across species ^97,99^. Thus, having been able to capture changes that inform on the viscoelastic properties of animals lacking MIG-6/papilin is a key finding, especially since few such *in vivo* measurements have been achieved to date ^88,100^.

The viscosity of a substrate is known to influence cell migration, with cells from normal tissue and tumor cells both exhibiting increased migration speed on highly viscous substrates or extracellular fluids ^101 {Bera, 2022 #723 102^. Also, both higher and lower cellular elasticity are linked to the motility of cancer cells ^103–105^. Conversely, cell adhesion can occur on the surface of low viscosity liquids ^106,107^. Thus, the decreased viscosity of *mig-6* mutants may somehow, possibly via distinct cell-ECM interactions, result in enhanced cell adhesion enabling neurons to maintain their normal architecture. In addition, *mig-6* mutants present a decrease in loss tangent that translates into decreased viscoelasticity, suggesting that their tissues exhibit more solid-like properties with reduced energy dissipation ^73^. Tissues and matrix mechanics are sensed by cells and converted into chemical signals through mechanotransduction ^97,108^. Thus, the decreased viscoelasticty in *mig-6* mutants, and the proposed associated reduction in energy dissipation, could modulate mechanosensing and regulate cellular responses ^109,110^, to better preserve tissue shape and maintain neuronal architecture. Indeed, this may be related to the altered collagen IV levels, organization, and remodeling that we uncovered in *mig-6* mutants, which could profoundly impact the overall ECM composition and organization. A parallel could be drawn with the excessive production of ECM components in tissue fibrosis, which results in decreased viscoelasticity ^108^. Such fibrotic states also lead to progressive matrix stiffening ^108^. The build-up of collagen IV occurring in *mig-6* mutants, and that depleting the crosslinking enzyme peroxidasin/PXN-2 attenuated their fibrotic state, suggests that collagen IV molecules in *mig-6* mutants have increased covalent sulfilimine cross-links, which could lead to increased ECM stiffness ^81,111^, and consequently, a likely decreased flexibility. Given the expected increased stiffness in *mig-6* mutants and the similar altered remodeling of ECM collagen IV in double *sax-7; mig-6* mutant animals, the mechanical proprieties arising from loss of *mig-6* could help maintain neuronal architecture through increased stiffness.

Overall, we propose that the animal’s biomechanical changes resulting from the loss of MIG-6/papilin are linked to their altered ECM state. The differences in biomechanical properties are likely to bring about changes in ECM-neuron interactions, and/or in the state of neurons, such that neuronal architecture is preserved, even in the absence of SAX-7/L1CAM, or in conditions of heightened physical stress from the incessant muscle contractions of continuous swimming. Future studies could further elucidate the underpinnings of this remarkable state resulting from changes in ECM remodeling by the conserved ECM regulator MIG-6/papilin, which safeguards neuronal architecture during post-natal life and into adulthood.

Interestingly, while MIG-6/papilin plays a crucial role in defining the state of the ECM, its effects are specific to the precise molecular landscapes in distinct neuronal structures of the animal. For instance, we found that whereas loss of *mig-6* suppresses the maintenance defects of axon position in the ventral nerve cord that are caused by the loss of adhesion molecule SAX-7/L1CAM, or by loss of basement membrane protein DIG-1, it fails to suppress similar maintenance defects of the same axons when caused by the loss of two-Ig domain proteins ZIG-3 and ZIG-4. Similarly, whereas loss of *mig-6* suppresses the head ganglia defects in both *sax-7/L1CAM* and *dig-1* mutants, it had no effect on tail ganglia maintenance defects displayed by *dig-1* mutants. These observations underscore the complexity of the molecular interactions involving distinct ECM networks that surround different neuronal assemblies. The specificity of MIG-6/papilin’s action is also evident in the different developmental consequences of *mig-6* mutations across different contexts, including the gonad, the pharynx, and the ALA-PVD neurons in the lateral nerve tract. This specificity is also reflected in its functional interaction with MIG-17/ADAMTS, which affects head ganglia maintenance and distal tip cell migration (this work,^40^), but not for ALA-PVD neuronal patterning ^46^. Future studies will help elucidate the interactions among other ECM components that may participate in the remodeling process orchestrated by papilin.

The extracellular matrix (ECM) has emerged as a key regulator of nervous system development and maintenance across diverse species ^112–114^. In *C. elegans*, the ECM modulates synaptic development ^115^, as well as synaptic maintenance, with collagen IV and metalloproteinase GON-1 being implicated in sustaining synapse morphology of neuromuscular junctions ^116 117^. In the mammalian central nervous system, the ECM is a large part of the neural tissue and serves various functions ranging from supporting cell migration, to regulating synaptic transmission and plasticity, to actively modulating the neural tissue after injury. In particular, the perineuronal nets (PNNs), a specialized form of ECM surrounding dendritic spines, have been shown to be dynamically regulated, impacting both structural and functional plasticity ^118^. The ECM composition of PNNs is regulated by the expression of proteases that target distinct PNN, enabling the transition from states of plasticity to stability ^119^. Disruptions in PNN composition is linked to neurodegenerative diseases ^13,14^. Collagen IV is a well-conserved component of basement membranes, including in the vertebrate central nervous system. It is conceivable that functional interactions between papilin, ADAMTS metalloproteinases and ECM molecules, similar to those described in this study, may also occur in mammals to maintain neuronal architecture throughout life.

Neuronal structures need to withstand deformations caused by the animal’s growth and body movements to prevent structural damage to neural circuits. How multicellular neuronal assemblies endure mechanical stress to sustain their architecture on the long term remains poorly understood. This work provides a mechanism by which the regulation of ECM remodeling enables and supports the maintenance of neuronal architecture postnatally and into adulthood. Other mechanisms previously described to critically impact the maintenance of neuronal architecture also rely on non-cell-autonomous biological functions. For instance, the secreted immunoglobulin proteins ZIG-3 and ZIG-4 are thought to stabilize axons positioning by modulating inter-axon adhesive properties ^30,31^. The cell adhesion molecule SAX-7/L1CAM mediates cell surface homophilic and heterophilic interactions between neurons and its neighboring cells (e.g, other neurons or epidermal) ^25,27,46^. The secreted basement membrane protein DIG-1 is proposed to bridge interactions between the basement membranes ensheathing neuronal structures and adjoining muscle cells ^28^. Collagen IV and ADAMTS/GON-1 ensures the maintenance of synaptic morphology at the neuromuscular junction ^116,117^. MIG-17/ADAMTS maintains synapse location and morphology during post-embryonic growth by modulating muscle basement membrane, which impacts interactions between epidermis, glia, and the associated synapses ^51,120^. The two-immunoglobulin domain protein ZIG-10 expressed on the epidermis underlying the nerve cord maintains synaptic density as the animal grows ^121^. More recently, the interplay between epithelial cells, their ECM, cell junctions and glial cells was shown to ensure the preservation of glia morphology in the face of environmental challenges, which in turn protects the associated neuron’s shape and function ^88,122^. Finally, cytoskeletal components too can act non-cell-autonomously from the underlying epidermis embedding the axon of a neuron to preserve its integrity ^123.^. Thus, the combined actions of both intrinsic and extrinsic mechanisms safeguard the intricate multicellular structures of the nervous system. Understanding general principles governing the long-term maintenance of the neuronal architectures underlying neural circuits is crucial for elucidating the bases of neurodegenerative conditions.

## Supplementary Material

Supplement 1

## References

[1] van Dyck L. I. & Morrow E. M. Genetic control of postnatal human brain growth. Current opinion in neurology 30, 114–124 (2017). 27898583 10.1097/WCO.0000000000000405 PMC5340196

[2] Heckman E. L. & Doe C. Q. Establishment and maintenance of neural circuit architecture. Journal of Neuroscience 41, 1119–1129 (2021). 33568445 10.1523/JNEUROSCI.1143-20.2020 PMC7888231

[3] Bénard C. & Hobert O. Looking beyond development: maintaining nervous system architecture. Current topics in developmental biology 87, 175–194 (2009). 19427520 10.1016/S0070-2153(09)01206-X

[4] Sultana O. F., Bandaru M., Islam M. A. & Reddy P. H. Unraveling the complexity of human brain: Structure, function in healthy and disease states. Ageing Research Reviews 100, 102414 (2024). 39002647 10.1016/j.arr.2024.102414 PMC11384519

[5] Hemphill M. A., Dauth S., Yu C. J., Dabiri B. E. & Parker K. K. Traumatic brain injury and the neuronal microenvironment: a potential role for neuropathological mechanotransduction. Neuron 85, 1177–1192 (2015). 25789754 10.1016/j.neuron.2015.02.041

[6] Lin Y.-C. & Koleske A. J. Mechanisms of synapse and dendrite maintenance and their disruption in psychiatric and neurodegenerative disorders. Annual review of neuroscience 33, 349–378 (2010). 10.1146/annurev-neuro-060909-153204 PMC3063389 20367247

[7] Mariano V., Domínguez-Iturza N., Neukomm L. J. & Bagni C. Maintenance mechanisms of circuit-integrated axons. Current opinion in neurobiology 53, 162–173 (2018). 30241058 10.1016/j.conb.2018.08.007

[8] Sauerbeck A. D. SEQUIN multiscale imaging of mammalian central synapses reveals loss of synaptic connectivity resulting from diffuse traumatic brain injury. Neuron 107, 257–273. e255 (2020). 32392471 10.1016/j.neuron.2020.04.012 PMC7381374

[9] Alberts B. Molecular Biology of the Cell: Seventh International Student Edition with Registration Card. (WW Norton & Company, 2022).

[10] Long K. R. & Huttner W. B. How the extracellular matrix shapes neural development. Royal Society Open Biology 9, 180216 (2019). 10.1098/rsob.180216 PMC6367132 30958121

[11] Benarroch E. E. Extracellular matrix in the CNS: dynamic structure and clinical correlations. Neurology 85, 1417–1427 (2015). 26400579 10.1212/WNL.0000000000002044

[12] Fawcett J. W., Oohashi T. & Pizzorusso T. The roles of perineuronal nets and the perinodal extracellular matrix in neuronal function. Nature Reviews Neuroscience 20, 451–465 (2019). 31263252 10.1038/s41583-019-0196-3

[13] Harkness J. H. Diurnal changes in perineuronal nets and parvalbumin neurons in the rat medial prefrontal cortex. Brain Structure and Function 226, 1135–1153 (2021). 33585984 10.1007/s00429-021-02229-4 PMC8086998

[14] Suttkus A., Holzer M., Morawski M. & Arendt T. The neuronal extracellular matrix restricts distribution and internalization of aggregated Tau-protein. Neuroscience 313, 225–235 (2016). 26621125 10.1016/j.neuroscience.2015.11.040

[15] Lu P., Takai K., Weaver V. M. & Werb Z. Extracellular matrix degradation and remodeling in development and disease. Cold Spring Harbor perspectives in biology 3, a005058 (2011). 21917992 10.1101/cshperspect.a005058 PMC3225943

[16] White J. G., Southgate E., Thomson J. N. & Brenner S. The structure of the nervous system of the nematode Caenorhabditis elegans. Philos Trans R Soc Lond B Biol Sci 314, 1–340 (1986). 22462104 10.1098/rstb.1986.0056

[17] Altun Z., Herndon L., Wolkow C., Crocker C., Lints R. & Hall D. (2024).

[18] Knight C. G., Patel M. N., Azevedo R. B. & Leroi A. M. A novel mode of ecdysozoan growth in Caenorhabditis elegans. Evolution & development 4, 16–27 (2002). 11871396 10.1046/j.1525-142x.2002.01058.x

[19] Witvliet D. Connectomes across development reveal principles of brain maturation. Nature 596, 257–261 (2021). 34349261 10.1038/s41586-021-03778-8 PMC8756380

[20] Boulin T., Etchberger J. F. & Hobert O. Reporter gene fusions. WormBook: The Online Review of C. elegans Biology [Internet] (2006). 10.1895/wormbook.1.106.1 PMC4781452 18050449

[21] Keeley D. P. Comprehensive endogenous tagging of basement membrane components reveals dynamic movement within the matrix scaffolding. Developmental cell 54, 60–74. e67 (2020). 32585132 10.1016/j.devcel.2020.05.022 PMC7394237

[22] Zallen J. A., Kirch S. A. & Bargmann C. I. Genes required for axon pathfinding and extension in the C. elegans nerve ring. Development 126, 3679–3692 (1999). 10409513 10.1242/dev.126.16.3679

[23] Sasakura H. Maintenance of neuronal positions in organized ganglia by SAX-7, a Caenorhabditis elegans homologue of L1. The EMBO journal 24, 1477–1488 (2005). 15775964 10.1038/sj.emboj.7600621 PMC1142545

[24] Wang X., Kweon J., Larson S. & Chen L. A role for the C. elegans L1CAM homologue lad-1/sax-7 in maintaining tissue attachment. Developmental biology 284, 273–291 (2005). 16023097 10.1016/j.ydbio.2005.05.020

[25] Pocock R., Bénard C. Y., Shapiro L. & Hobert O. Functional dissection of the C. elegans cell adhesion molecule SAX-7, a homologue of human L1. Molecular and Cellular Neuroscience 37, 56–68 (2008). 17933550 10.1016/j.mcn.2007.08.014

[26] Zhou S. & Chen L. Neural integrity is maintained by dystrophin in C. elegans. Journal of Cell Biology 192, 349–363 (2011). 21242290 10.1083/jcb.201006109 PMC3172177

[27] Desse V. E. Neuronal postdevelopmentally acting SAX-7S/L1CAM can function as cleaved fragments to maintain neuronal architecture in Caenorhabditis elegans. Genetics 218, iyab086 (2021). 34115111 10.1093/genetics/iyab086 PMC8883803

[28] Bénard C. Y., Boyanov A., Hall D. H. & Hobert O. DIG-1, a novel giant protein, non-autonomously mediates maintenance of nervous system architecture. (2006). 10.1242/dev.02507 16887823

[29] Johnson R. P. & Kramer J. M. Neural maintenance roles for the matrix receptor dystroglycan and the nuclear anchorage complex in Caenorhabditis elegans. Genetics 190, 1365–1377 (2012). 22298703 10.1534/genetics.111.136184 PMC3316649

[30] Aurelio O., Hall D. H. & Hobert O. Immunoglobulin-domain proteins required for maintenance of ventral nerve cord organization. Science 295, 686–690 (2002). 11809975 10.1126/science.1066642

[31] Bénard C., Tjoe N., Boulin T., Recio J. & Hobert O. The small, secreted immunoglobulin protein ZIG-3 maintains axon position in Caenorhabditis elegans. Genetics 183, 917–927 (2009). 19737747 10.1534/genetics.109.107441 PMC2778987

[32] Bülow H. E., Boulin T. & Hobert O. Differential functions of the C. elegans FGF receptor in axon outgrowth and maintenance of axon position. Neuron 42, 367–374 (2004). 15134634 10.1016/s0896-6273(04)00246-6

[33] Aurelio O., Boulin T. & Hobert O. Identification of spatial and temporal cues that regulate postembryonic expression of axon maintenance factors in the C. elegans ventral nerve cord. (2003). 10.1242/dev.00277 12490565

[34] Law J. W. Decreased anxiety, altered place learning, and increased CA1 basal excitatory synaptic transmission in mice with conditional ablation of the neural cell adhesion molecule L1. Journal of Neuroscience 23, 10419–10432 (2003). 14614101 10.1523/JNEUROSCI.23-32-10419.2003 PMC6741026

[35] Tai Y., Gallo N. B., Wang M., Yu J.-R. & Van Aelst L. Axo-axonic innervation of neocortical pyramidal neurons by GABAergic chandelier cells requires AnkyrinG-associated L1CAM. Neuron 102, 358–372. e359 (2019). 30846310 10.1016/j.neuron.2019.02.009 PMC6525570

[36] Kramerova I. A. Papilin in development; a pericellular protein with a homology to the ADAMTS metalloproteinases. Development 127, 5475–5485 (2000). 11076767 10.1242/dev.127.24.5475

[37] Doitsidou M., Poole R. J., Sarin S., Bigelow H. & Hobert O. C. elegans mutant identification with a one-step whole-genome-sequencing and SNP mapping strategy. PloS one 5, e15435 (2010). 21079745 10.1371/journal.pone.0015435 PMC2975709

[38] Minevich G., Park D. S., Blankenberg D., Poole R. J. & Hobert O. CloudMap: a cloud-based pipeline for analysis of mutant genome sequences. Genetics 192, 1249–1269 (2012). 23051646 10.1534/genetics.112.144204 PMC3512137

[39] Doitsidou M., Jarriault S. & Poole R. J. Next-generation sequencing-based approaches for mutation mapping and identification in Caenorhabditis elegans. Genetics 204, 451–474 (2016). 27729495 10.1534/genetics.115.186197 PMC5068839

[40] Kawano T. C. elegans mig-6 encodes papilin isoforms that affect distinct aspects of DTC migration, and interacts genetically with mig-17 and collagen IV. (2009). 10.1242/dev.028472 PMC2674254 19297413

[41] Campbell A., Fessler L., Salo T. & Fessler J. Papilin: a Drosophila proteoglycan-like sulfated glycoprotein from basement membranes. Journal of Biological Chemistry 262, 17605–17612 (1987). 3320045

[42] Kramerova I. A., Kramerov A. A. & Fessler J. H. Alternative splicing of papilin and the diversity of Drosophila extracellular matrix during embryonic morphogenesis. Developmental dynamics: an official publication of the American Association of Anatomists 226, 634–642 (2003). 12666201 10.1002/dvdy.10265

[43] Fessler J. H., Kramerova I., Kramerov A., Chen Y. & Fessler L. I. Papilin, a novel component of basement membranes, in relation to ADAMTS metalloproteases and ECM development. The international journal of biochemistry & cell biology 36, 1079–1084 (2004). 15094122 10.1016/j.biocel.2003.12.010

[44] Jafari G. Genetics of extracellular matrix remodeling during organ growth using the Caenorhabditis elegans pharynx model. Genetics 186, 969–982 (2010). 20805556 10.1534/genetics.110.120519 PMC2975278

[45] Lacin H., Zhu Y., DiPaola J. T., Wilson B. A., Zhu Y. & Skeath J. B. A genetic screen in Drosophila uncovers a role for senseless-2 in surface glia in the peripheral nervous system to regulate CNS morphology. G3: Genes, Genomes, Genetics 14, jkae152 (2024). 38996053 10.1093/g3journal/jkae152 PMC11373656

[46] Ramirez-Suarez N. J. Axon-dependent patterning and maintenance of somatosensory dendritic arbors. Developmental cell 48, 229–244. e224 (2019). 30661986 10.1016/j.devcel.2018.12.015 PMC6442679

[47] Kelwick R., Desanlis I., Wheeler G. N. & Edwards D. R. The ADAMTS (A Disintegrin and Metalloproteinase with Thrombospondin motifs) family. Genome biology 16, 1–16 (2015). 26025392 10.1186/s13059-015-0676-3 PMC4448532

[48] Apte S. S. A disintegrin-like and metalloprotease (reprolysin-type) with thrombospondin type 1 motif (ADAMTS) superfamily: functions and mechanisms. Journal of Biological Chemistry 284, 31493–31497 (2009). 19734141 10.1074/jbc.R109.052340 PMC2797218

[49] Hobert O. & Bülow H. Development and maintenance of neuronal architecture at the ventral midline of C. elegans. Current opinion in neurobiology 13, 70–78 (2003). 12593984 10.1016/s0959-4388(03)00002-3

[50] Shibata Y., Huang Y., Yoshida M. & Nishiwaki K. Mutations in fibulin-1 and collagen IV suppress the short healthspan of mig-17/ADAMTS mutants in Caenorhabditis elegans. Plos one 19, e0305396 (2024). 38980840 10.1371/journal.pone.0305396 PMC11232982

[51] Fan J. A muscle-epidermis-glia signaling axis sustains synaptic specificity during allometric growth in Caenorhabditis elegans. Elife 9, e55890 (2020). 32255430 10.7554/eLife.55890 PMC7164957

[52] Kubota Y., Ohkura K., Tamai K. K., Nagata K. & Nishiwaki K. MIG-17/ADAMTS controls cell migration by recruiting nidogen to the basement membrane in C. elegans. Proceedings of the National Academy of Sciences 105, 20804–20809 (2008). 10.1073/pnas.0804055106 PMC2634947 19104038

[53] Ihara S. & Nishiwaki K. Stage-specific activation of MIG-17/ADAMTS controls cell migration in Caenorhabditis elegans. The FEBS Journal 275, 4296–4305 (2008). 18637819 10.1111/j.1742-4658.2008.06573.x

[54] Nishiwaki K., Hisamoto N. & Matsumoto K. A metalloprotease disintegrin that controls cell migration in Caenorhabditis elegans. Science 288, 2205–2208 (2000). 10864868 10.1126/science.288.5474.2205

[55] Shibata Y. Organ length control by an ADAMTS extracellular protease in Caenorhabditis elegans. G3: Genes, Genomes, Genetics 6, 1449–1457 (2016). 26994289 10.1534/g3.116.028019 PMC4856095

[56] Fernando T. C. elegans ADAMTS ADT-2 regulates body size by modulating TGFβ signaling and cuticle collagen organization. Developmental biology 352, 92–103 (2011). 21256840 10.1016/j.ydbio.2011.01.016 PMC3049821

[57] Cao J. Comprehensive single-cell transcriptional profiling of a multicellular organism. Science 357, 661–667 (2017). 28818938 10.1126/science.aam8940 PMC5894354

[58] Roux A. E. Individual cell types in C. elegans age differently and activate distinct cell-protective responses. Cell Reports 42 (2023). 10.1016/j.celrep.2023.112902 37531250

[59] Guo X. & Kramer J. The two Caenorhabditis elegans basement membrane (type IV) collagen genes are located on separate chromosomes. Journal of Biological Chemistry 264, 17574–17582 (1989). 2793871

[60] Guo X., Johnson J. J. & Kramer J. M. Embryonic lethality caused by mutations in basement membrane collagen of C. elegans. Nature 349, 707–709 (1991). 1996137 10.1038/349707a0

[61] Sibley M. H., Johnson J. J., Mello C. C. & Kramer J. M. Genetic identification, sequence, and alternative splicing of the Caenorhabditis elegans alpha 2 (IV) collagen gene. The Journal of cell biology 123, 255–264 (1993). 7691828 10.1083/jcb.123.1.255 PMC2119826

[62] Kramer J. M. Basement membranes. WormBook: The Online Review of C. elegans Biology [Internet] (2005). 10.1895/wormbook.1.16.1 PMC4781274 18050423

[63] Ihara S. Basement membrane sliding and targeted adhesion remodels tissue boundaries during uterine–vulval attachment in Caenorhabditis elegans. Nature cell biology 13, 641–651 (2011). 21572423 10.1038/ncb2233 PMC3107347

[64] Graham P. L., Johnson J. J., Wang S., Sibley M. H., Gupta M. C. & Kramer J. M. Type IV collagen is detectable in most, but not all, basement membranes of Caenorhabditis elegans and assembles on tissues that do not express it. The Journal of cell biology 137, 1171–1183 (1997). 9166416 10.1083/jcb.137.5.1171 PMC2136211

[65] Gupta M. C., Graham P. L. & Kramer J. M. Characterization of α1 (IV) collagen mutations in Caenorhabditis elegans and the effects of α1 and α2 (IV) mutations on type IV collagen distribution. The Journal of cell biology 137, 1185–1196 (1997). 9166417 10.1083/jcb.137.5.1185 PMC2136222

[66] Warren C. E., Krizus A. & Dennis J. W. Complementary expression patterns of six nonessential Caenorhabditis elegans core 2/IN-acetylglucosaminyltransferase homologues. Glycobiology 11, 979–988 (2001). 11744632 10.1093/glycob/11.11.979

[67] Katayama H., Yamamoto A., Mizushima N., Yoshimori T. & Miyawaki A. GFP-like proteins stably accumulate in lysosomes. Cell structure and function 33, 1–12 (2008). 18256512 10.1247/csf.07011

[68] Scarcelli G. Noncontact three-dimensional mapping of intracellular hydromechanical properties by Brillouin microscopy. Nature methods 12, 1132–1134 (2015). 26436482 10.1038/nmeth.3616 PMC4666809

[69] Coker Z. N. Brillouin microscopy monitors rapid responses in subcellular compartments. PhotoniX 5, 9 (2024). 38618142 10.1186/s43074-024-00123-w PMC11006764

[70] Bevilacqua C. High-resolution line-scan Brillouin microscopy for live imaging of mechanical properties during embryo development. Nature Methods 20, 755–760 (2023). 36997817 10.1038/s41592-023-01822-1 PMC10172129

[71] Prevedel R., Diz-Muñoz A., Ruocco G. & Antonacci G. Brillouin microscopy: an emerging tool for mechanobiology. Nature methods 16, 969–977 (2019). 31548707 10.1038/s41592-019-0543-3

[72] Bevilacqua C., Sánchez-Iranzo H., Richter D., Diz-Muñoz A. & Prevedel R. Imaging mechanical properties of sub-micron ECM in live zebrafish using Brillouin microscopy. Biomedical Optics Express 10, 1420–1431 (2019). 30891356 10.1364/BOE.10.001420 PMC6420298

[73] Chan C. J., Bevilacqua C. & Prevedel R. Mechanical mapping of mammalian follicle development using Brillouin microscopy. Communications biology 4, 1133 (2021). 34580426 10.1038/s42003-021-02662-5 PMC8476509

[74] Imanishi A. Genetic interactions among ADAMTS metalloproteases and basement membrane molecules in cell migration in Caenorhabditis elegans. Plos one 15, e0240571 (2020). 33264296 10.1371/journal.pone.0240571 PMC7710118

[75] Kubota Y., Nishiwaki K., Ito M. & Sugimoto A. The role of tissue inhibitors of metalloproteinases in organ development and regulation of ADAMTS family metalloproteinases in Caenorhabditis elegans. Genetics 212, 523–535 (2019). 30992386 10.1534/genetics.119.301795 PMC6553827

[76] Vanacore R. A sulfilimine bond identified in collagen IV. Science 325, 1230–1234 (2009). 19729652 10.1126/science.1176811 PMC2876822

[77] Khoshnoodi J., Cartailler J.-P., Alvares K., Veis A. & Hudson B. G. Molecular recognition in the assembly of collagens: terminal noncollagenous domains are key recognition modules in the formation of triple helical protomers. Journal of Biological Chemistry 281, 38117–38121 (2006). 17082192 10.1074/jbc.R600025200

[78] Fidler A. L. A unique covalent bond in basement membrane is a primordial innovation for tissue evolution. Proceedings of the National Academy of Sciences 111, 331–336 (2014). 10.1073/pnas.1318499111 PMC3890831 24344311

[79] He C. Peroxidasin-mediated bromine enrichment of basement membranes. Proceedings of the National Academy of Sciences 117, 15827–15836 (2020). 10.1073/pnas.2007749117 PMC7354931 32571911

[80] McCall A. S., Cummings C. F., Bhave G., Vanacore R., Page-McCaw A. & Hudson B. G. Bromine is an essential trace element for assembly of collagen IV scaffolds in tissue development and architecture. Cell 157, 1380–1392 (2014). 24906154 10.1016/j.cell.2014.05.009 PMC4144415

[81] Bhave G., Colon S. & Ferrell N. The sulfilimine cross-link of collagen IV contributes to kidney tubular basement membrane stiffness. American Journal of Physiology-Renal Physiology 313, F596–F602 (2017). 28424209 10.1152/ajprenal.00096.2017 PMC5625101

[82] Bhave G. Peroxidasin forms sulfilimine chemical bonds using hypohalous acids in tissue genesis. Nature chemical biology 8, 784–790 (2012). 22842973 10.1038/nchembio.1038 PMC4128002

[83] Gotenstein J. R., Koo C. C., Ho T. W. & Chisholm A. D. Genetic suppression of basement membrane defects in Caenorhabditis elegans by gain of function in extracellular matrix and cell-matrix attachment genes. Genetics 208, 1499–1512 (2018). 29440357 10.1534/genetics.118.300731 PMC5887144

[84] Pierce-Shimomura J. T., Chen B. L., Mun J. J., Ho R., Sarkis R. & McIntire S. L. Genetic analysis of crawling and swimming locomotory patterns in C. elegans. Proceedings of the National Academy of Sciences 105, 20982–20987 (2008). 10.1073/pnas.0810359105 PMC2634943 19074276

[85] Berri S., Boyle J. H., Tassieri M., Hope I. A. & Cohen N. Forward locomotion of the nematode C. elegans is achieved through modulation of a single gait. HFSP journal 3, 186–193 (2009). 19639043 10.2976/1.3082260 PMC2714959

[86] Fang-Yen C. Biomechanical analysis of gait adaptation in the nematode Caenorhabditis elegans. Proceedings of the National Academy of Sciences 107, 20323–20328 (2010). 10.1073/pnas.1003016107 PMC2996704 21048086

[87] Sznitman J., Purohit P. K., Krajacic P., Lamitina T. & Arratia P. E. Material properties of Caenorhabditis elegans swimming at low Reynolds number. Biophysical journal 98, 617–626 (2010). 20159158 10.1016/j.bpj.2009.11.010 PMC2820645

[88] Coraggio F. Age-progressive interplay of HSP-proteostasis, ECM-cell junctions and biomechanics ensures C. elegans astroglial architecture. Nature Communications 15, 2861 (2024). 10.1038/s41467-024-46827-2 PMC10991496 38570505

[89] Kuno K. & Matsushima K. ADAMTS-1 protein anchors at the extracellular matrix through the thrombospondin type I motifs and its spacing region. Journal of Biological Chemistry 273, 13912–13917 (1998). 9593739 10.1074/jbc.273.22.13912

[90] Ihara S. & Nishiwaki K. Prodomain-dependent tissue targeting of an ADAMTS protease controls cell migration in Caenorhabditis elegans. The EMBO Journal 26, 2607–2620 (2007). 17491590 10.1038/sj.emboj.7601718 PMC1888677

[91] Pearson J. R. ECM-Regulator timp is required for stem cell niche organization and cyst production in the Drosophila ovary. PLoS genetics 12, e1005763 (2016). 26808525 10.1371/journal.pgen.1005763 PMC4725958

[92] Krasselt K., Frommelt C., Brunner R., Rauscher F. G., Francke M. & Körber N. Various cross-linking methods inhibit the collagenase I degradation of rabbit scleral tissue. BMC ophthalmology 20, 1–10 (2020). 33317477 10.1186/s12886-020-01751-z PMC7734860

[93] Zhang Y., Mao X., Schwend T., Littlechild S. & Conrad G. W. Resistance of corneal RFUVA–cross-linked collagens and small leucine-rich proteoglycans to degradation by matrix metalloproteinases. Investigative ophthalmology & visual science 54, 1014–1025 (2013). 23322569 10.1167/iovs.12-11277 PMC4604715

[94] Potekaev N. N. Genetic and epigenetic aspects of skin collagen fiber turnover and functioning. Cosmetics 8, 92 (2021).

[95] Gotenstein J. R. The C. elegans peroxidasin PXN-2 is essential for embryonic morphogenesis and inhibits adult axon regeneration. Development 137, 3603–3613 (2010). 20876652 10.1242/dev.049189 PMC2964093

[96] Khoshnoodi J., Pedchenko V. & Hudson B. G. Mammalian collagen IV. Microscopy research and technique 71, 357–370 (2008). 18219669 10.1002/jemt.20564 PMC4788096

[97] Elosegui-Artola A. The extracellular matrix viscoelasticity as a regulator of cell and tissue dynamics. Current opinion in cell biology 72, 10–18 (2021). 33993058 10.1016/j.ceb.2021.04.002

[98] Urbanczyk M., Layland S. L. & Schenke-Layland K. The role of extracellular matrix in biomechanics and its impact on bioengineering of cells and 3D tissues. Matrix Biology 85, 1–14 (2020). 31805360 10.1016/j.matbio.2019.11.005

[99] Chaudhuri O., Cooper-White J., Janmey P. A., Mooney D. J. & Shenoy V. B. Effects of extracellular matrix viscoelasticity on cellular behaviour. Nature 584, 535–546 (2020). 32848221 10.1038/s41586-020-2612-2 PMC7676152

[100] Yang F. Pulsed stimulated Brillouin microscopy enables high-sensitivity mechanical imaging of live and fragile biological specimens. Nature Methods 20, 1971–1979 (2023). 37884795 10.1038/s41592-023-02054-z PMC10703689

[101] Shu W. & Kaplan C. in APS March Meeting Abstracts. W07. 006.

[102] Murrell M., Kamm R. & Matsudaira P. Substrate viscosity enhances correlation in epithelial sheet movement. Biophysical journal 101, 297–306 (2011). 21767481 10.1016/j.bpj.2011.05.048 PMC3136779

[103] Nikolić M., Scarcelli G. & Tanner K. Multimodal microscale mechanical mapping of cancer cells in complex microenvironments. Biophysical Journal 121, 3586–3599 (2022). 36059196 10.1016/j.bpj.2022.09.002 PMC9617162

[104] Conrad C., Gray K. M., Stroka K. M., Rizvi I. & Scarcelli G. Mechanical characterization of 3D ovarian cancer nodules using Brillouin confocal microscopy. Cellular and molecular bioengineering 12, 215–226 (2019). 31719911 10.1007/s12195-019-00570-7 PMC6816613

[105] Mierke C. T. Viscoelasticity, like forces, plays a role in mechanotransduction. Frontiers in Cell and Developmental Biology 10, 789841 (2022). 35223831 10.3389/fcell.2022.789841 PMC8864183

[106] Kong D., Megone W., Nguyen K. D., Di Cio S., Ramstedt M. & Gautrot J. E. Protein nanosheet mechanics controls cell adhesion and expansion on low-viscosity liquids. Nano Letters 18, 1946–1951 (2018). 29411615 10.1021/acs.nanolett.7b05339

[107] Cantini M., Donnelly H., Dalby M. J. & Salmeron-Sanchez M. The plot thickens: the emerging role of matrix viscosity in cell mechanotransduction. Advanced healthcare materials 9, 1901259 (2020). 10.1002/adhm.201901259 31815372

[108] Hui E., Moretti L., Barker T. H. & Caliari S. R. The combined influence of viscoelastic and adhesive cues on fibroblast spreading and focal adhesion organization. Cellular and molecular bioengineering 14, 427–440 (2021). 34777602 10.1007/s12195-021-00672-1 PMC8548477

[109] Clément R., Dehapiot B., Collinet C., Lecuit T. & Lenne P.-F. Viscoelastic dissipation stabilizes cell shape changes during tissue morphogenesis. Current biology 27, 3132–3142. e3134 (2017). 28988857 10.1016/j.cub.2017.09.005

[110] Huerta-López C. Cell response to extracellular matrix viscous energy dissipation outweighs high-rigidity sensing. Science advances 10, eadf9758 (2024). 39546608 10.1126/sciadv.adf9758 PMC11567001

[111] Nabavizadeh A. Viscoelastic biomarker for differentiation of benign and malignant breast lesion in ultra-low frequency range. Scientific reports 9, 5737 (2019). 30952880 10.1038/s41598-019-41885-9 PMC6450913

[112] Sorg B. A. Casting a wide net: role of perineuronal nets in neural plasticity. Journal of Neuroscience 36, 11459–11468 (2016). 27911749 10.1523/JNEUROSCI.2351-16.2016 PMC5125213

[113] Page-McCaw A. in Seminars in cell & developmental biology. 14–23 (Elsevier).

[114] Miller C. M., Page-McCaw A. & Broihier H. T. Matrix metalloproteinases promote motor axon fasciculation in the Drosophila embryo. (2008). 10.1242/dev.011072 18045838

[115] Ackley B. D., Kang S. H., Crew J. R., Suh C., Jin Y. & Kramer J. M. The Basement Membrane Components Nidogen and Type XVIII Collagen Regulate Organization of Neuromuscular Junctions inCaenorhabditis elegans. Journal of Neuroscience 23, 3577–3587 (2003). 12736328 10.1523/JNEUROSCI.23-09-03577.2003 PMC6742194

[116] Kurshan P. T., Phan A. Q., Wang G. J., Crane M. M., Lu H. & Shen K. Regulation of synaptic extracellular matrix composition is critical for proper synapse morphology. Journal of Neuroscience 34, 12678–12689 (2014). 25232106 10.1523/JNEUROSCI.1183-14.2014 PMC4166155

[117] Qin J., Liang J. & Ding M. Perlecan antagonizes collagen IV and ADAMTS9/GON-1 in restricting the growth of presynaptic boutons. Journal of Neuroscience 34, 10311–10324 (2014). 25080592 10.1523/JNEUROSCI.5128-13.2014 PMC6608278

[118] Wlodarczyk J., Mukhina I., Kaczmarek L. & Dityatev A. Extracellular matrix molecules, their receptors, and secreted proteases in synaptic plasticity. Developmental neurobiology 71, 1040–1053 (2011). 21793226 10.1002/dneu.20958

[119] Dansie L. E. & Ethell I. M. Casting a net on dendritic spines: the extracellular matrix and its receptors. Developmental neurobiology 71, 956–981 (2011). 21834084 10.1002/dneu.20963 PMC3192312

[120] Shao Z., Watanabe S., Christensen R., Jorgensen E. M. & Colón-Ramos D. A. Synapse location during growth depends on glia location. Cell 154, 337–350 (2013). 23870123 10.1016/j.cell.2013.06.028 PMC3808971

[121] Cherra S. J. & Jin Y. A two-immunoglobulin-domain transmembrane protein mediates an epidermal-neuronal interaction to maintain synapse density. Neuron 89, 325–336 (2016). 26777275 10.1016/j.neuron.2015.12.024 PMC4871750

[122] Martin C. G., Bent J. S., Hill T., Topalidou I. & Singhvi A. Epithelial UNC-23 limits mechanical stress to maintain glia-neuron architecture in C. elegans. Developmental Cell (2024). 10.1016/j.devcel.2024.04.005 PMC11233253 38670103

[123] Coakley S., Ritchie F. K., Galbraith K. M. & Hilliard M. A. Epidermal control of axonal attachment via β-spectrin and the GTPase-activating protein TBC-10 prevents axonal degeneration. Nature Communications 11, 133 (2020). 10.1038/s41467-019-13795-x PMC6952388 31919407

