# Supplementary material for: Remodeling of extracellular matrix collagen IV by MIG-6/papilin regulates neuronal architecture
Source: Res Sq. 2025 Feb 14:rs.3.rs-5962240. Preprint. [Version 1] doi: 10.21203/rs.3.rs-5962240/v1 (PMC11844652; doi:10.21203/rs.3.rs-5962240/v1)
Supplement: Supplement 1 [file NIHPPrs5962240v1-supplement-1.pdf]

Supplementary Materials

for

Remodeling of extracellular matrix collagen IV by MIG-6/papilin regulates neuronal architecture

Malika NADOUR[1,2], Robert I. VALETTE REVENO LEATIS[1,2], Marie BIARD[1,2], Noémie FRÉBAULT[1,2], Lise RIVOLLET[1,2], Philippe ST-LOUIS[1,2], Cassandra R. BLANCHETTE[3], Andrea THACKERAY[3], Paola PERRAT[3], Carlo BEVILACQUA[4], Robert PREVEDEL[4-7], Laurent CAPPADOCIA[2,8,9], Georgia RAPTI[5-7,10], Maria DOITSIDOU[11], Claire Y. BÉNARD[1,2,3,*]

This PDF file includes:

- **Materials and Methods**
- **Supplementary Figures and Legends**
- **Supplementary Tables**

  Table S1. List of strain used
  Table S2. Information on transgenic strains used
  Table S3. List of primers used

- **Supplementary Movies Description**

  Supplementary Movie 1. *mig-6(qv33)* mutant animals display increased collagen IV and fibrotic-like structures phenotype
  Supplementary Movie 2. *mig-6(qv33)* mutant animals display increased collagen IV and fibrotic-like structures phenotype
  Supplementary Movie 3. Normal pattern of collagen IV in the wild type

- **Source Data for Figures**
- **Source Data for Supplementary Figures**

MATERIALS AND METHODS

***C. elegans* strains and genetics**. Strains were cultured at 20°C on nematode growth medium (NGM) agar plates seeded with OP50 bacteria as described [1], unless otherwise specified. N2 is the reference wild-type strain. Mutant alleles and reporter strains were outcrossed at least three times prior to strain generation or analysis (listed in **Table S1**). Strains were constructed using standard genetic procedures and are listed in **Table S1** and **Table S2**. Genotypes were confirmed by visible phenotypes when possible, and by PCR or sequencing (primers listed in **Table S3**).

EMS forward genetic screen. We carried out a forward F2 clonal genetic screen for modifiers of neuronal maintenance defects using VQ90 *sax-7(qv24); zig-5(ok1065) zig-8(ok561); glp-4(bn2ts); oyIs14* as our screening strain, which was maintained at 15°C. *glp-4(bn2ts)* was used for efficient examination of adult worms, as it is a temperature-sensitive sterile mutation when shifted to 25°C [2]. Allele *qv24* of *sax*-7 will be described elsewhere (C.R.B and C.Y.B., unpub. results). For each mutagenesis round, VQ90 L4 worms were mutagenized with 25 µM ethyl methanesulfonate and allowed to recover at 15°C. Following recovery, five P_0 mutagenized worms were plated onto five plates (25 P_0s in total) and put at 15°C to lay their broods. 50 F1s from each P_0 plate were picked singly as L4 or young adults. From each of the 250 F1s, seven F2s were picked clonally (1750 F2s per round of mutagenesis) and grown at 15°C for two days (plates containing singled F1s were kept at 15°C for future use). After two days, the plates where single F2 animals had been picked were shifted to 25°C. When the F3 animals were 5-day-old adults or older, they were examined under a Zeiss V8 Discovery fluorescence stereoscope to identify rare broods exhibiting a modified phenotype compared to VQ90 control. Upon identification of a candidate modifier mutation, single animals were picked clonally from the corresponding F1 plate (kept at 15°C) to re-isolate the modifier, and their progeny were mounted on slides to examine and quantify the phenotype using an upright Zeiss Axioskop 2 Plus fluorescence microscope. If the modifier mutation was re-isolated and exhibited a robust phenotype, we outcrossed it. For outcrosses, we used males from the non-mutagenized strain VQ47 *sax-7(qv24); zig-5(ok1065) zig-8(ok561); hdIs29*, which is similar to the one used in the screen, but carries a red fluorescent marker to readily identify cross progeny. We thus could determine if the new modifier mutation was heritable, and at the same time remove *glp-4(bn2ts)*, and outcross other irrelevant EMS-induced mutations from the genetic background. Subsequent outcrosses with wild-type N2 males ensured that all strains used are wild type for *zig-5, zig-8,* and *glp-4*.

Isolation and molecular identification of *qv18*. *qv18* was isolated as a suppressor of the *sax7(qv24)* neuronal maintenance defects in the genetic screen described above. Outcrosses of the new strain bearing the suppressor mutation *qv18* (as described above) showed that the suppressor was heritable, penetrant, and likely monogenic. We also noticed abnormal segregation ratios, consistent with a possible chromosomal rearrangement resulting from mutagenesis; we therefore later engineered the *qv18* causal suppressor mutation in a clean genetic background. To identify the causal mutation in *qv18*, we performed whole genome sequencing on the pooled DNA of 18 independent suppressor lines (each line derived from one of 18 independent F2s from the outcross, with careful re-homozygosing of the suppression of the neuronal maintenance defect confirmed over 3 generations [3]. The sequencing data were uploaded to the public server at usegalaxy.org and we used the Cloudmap pipeline to analyze the data [4], which pointed to a *qv18* linkage region on chromosome V. A candidate variant therein was a mutation in the gene *mig-6*, which we pursued further by (i) performing rescue experiments, (ii) introducing the candidate mutation by genome editing in *sax-7* mutants with an otherwise wild-type *mig-6* gene (which yielded *qv33*), (iii) analyzing the effect of loss of *mig-6* function by *mig-6*(RNAi) experiments, and (iv) examining other *mig-6* mutant alleles.

Generation of *qv33* by CRISPR-Cas9. To confirm the molecular identity of the causal mutation suppressing *sax-7* defects in the *qv18* suppressor strain, the candidate *mig-6* mutation was reintroduced in a clean *sax-7(qv30);*

oyIs14 genetic background using CRISPR-Cas9 genome editing. A target sequence against *mig-6* was selected (ggtaccgctgaatgtggtgg) and cloned to generate a gRNA plasmid (pPP30). A 100 nt oligo carrying the candidate *qv18* missense mutation, at nucleotide 3409 of cosmid C37C3, served as the repair template (oCB1599 acatggtacacatcttcatggtccgagtgtaccgctgaatgtggtggtgAatcccaagatcgtgtcgctgtttgcttgaactacgataagaagccagttc, IDT synthesized). A DNA mix containing the Cas9 plasmid (pDD162), the gRNA plasmid pJA58 (*dpy-10* target) and *dpy-10(cn64)* ssDNA repair template for CRISPR co-conversion, as well as the *qv18* gRNA plasmid pPP30 and repair template oCB1599 was injected as described [5,6]. Rol or Dpy F1 animals were picked clonally and their F2 progeny were screened by PCR (using primers listed in Table S1), followed by restriction enzyme digestion, as a BamHI site present in the wild-type sequence is disrupted by the *qv18/qv33* single nucleotide substitution.

The same CRISPR-Cas9 strategy was used to generate the double mutant *mig-6(qv33) mig-17(k174)*, by introducing *qv33* into the *mig-17(k174)* background, as the loci are 2 cM apart on chromosome V.

Prediction and analysis of the three-dimensional structure of MIG-6S. The three-dimensional structure of MIG-6S (Uniprot accession O76840-2) was predicted using ColabFold 1.5.2 locally installed on the servers of the Digital Research Alliance of Canada. This version of ColabFold uses AlphaFold 2.3.1 and MMSeqs2 14-7e284. The depicted structure corresponds to the model presenting the highest pLDDT values that was obtained using a recycle count of 12. The images of the structure were made using PyMOL 2.5.4. Sequence logos were generated using Weblogo 3.7.12 and an alignment of 250 protein sequences from Ecdysozoa species, including nematodes and arthropods obtained using MMSeqs2.

Visualization and quantification of neuroanatomy and EMB-9/collagen IV structures. 1st larval stage (L1), 2nd larval stage (L2), 4th larval stage animals (L4), or 2-day old adult (selected as late L4 and observed 48 hours later) nematodes were mounted on agarose pads, immobilized with 75 mM NaN_3. These animals were observed under Nomarski or fluorescence microscopy Axio Scope.A1 or Axio Imager.M2 (Zeiss), with a 40x objective or a 100x oil immersion objective (for ventral nerve cord examination). Images were acquired using an AxioCam camera (Zeiss) and processed using ZEN (Zeiss).

Analysis of ASH and ASI. The cell bodies of ASH/ASI and their axons located in the nerve ring were visualized in L2, L4, and 2-day old adults, using reporter P*sra-6::DsRed2* (*hdIs26*). In the wild type, the ASHL/R and the ASIL/R soma are located posterior to the nerve ring. An animal was counted as mutant when along the antero-posterior axis of the animal, at least one of the ASH/ASI soma was not posterior to the nerve ring (but either anterior to or flanking the nerve ring).

Analysis of PVQ. The axons of neurons PVQL/R in the ventral nerve cord were visualized using P*sra-6::DsRed2* (*hdIs26*), in freshly hatched L1 and in L4 larvae, as described [7]. Briefly, in the wildtype, the axon of PVQL is positioned along the left fascicle of the ventral nerve cord, whereas the axon of PVQR is in the right fascicle. Animals were counted as having an axon flip-over defect when an axon was flipped to the opposite fascicle at any point along the ventral nerve cord.

Analysis of collagen IV fibrotic-like structures. We consider as fibrotic collagen IV the elongated structures or enrichments of EMB-9::mCherry signal (also reporters EMB-9::Dendra or EMB-9::mNG) that are observed in the posterior head region of *mig-6* or *mig-17* mutants (see **Figs. 4 and 6**). These structures are very rarely observed in the wild type, and they are different from the wild-type pattern of collagen IV signal present along the body wall muscles basal lamina (collagen IV concentrates in the basal lamina underneath each dense body and M line of sarcomeric muscles, including in the head region). To unequivocally assess the presence of collagen IV fibrotic-like structures in each animal, z-stacks were captured, and all the z-planes images were examined (the experimenter was blinded for genotype). Collagen IV fibrotic structures vary in number, length, and position (see **Fig. 4B, D, F**); animals displaying at least one such structure were counted as having fibrotic collagen IV.

As a result of the *pxn-2*(RNAi) (**Fig. 7F-G**), a different collagen IV pattern occurred, where the fibrotic-like structures appeared 'fragmented'. We consider a fibrotic like-structure to be fragmented if accumulation of collagen IV appeared as a series of elongated puncta, which were connected to one another by a thin line of collagen IV.

Microscopy and fluorescence intensity quantification. Animals were observed at precise ages, namely "young adults" (just molted from the L4 stage, <3 hours post L4 molt) and "day 2 adults" (48 hours after the young adult stage), mounted on 5% agarose pads with 75 mM NaN_3. Images were acquired as a z-stack using a Plan Apo 40x/0.95 NA objective on a Zeiss Axio Imager.M2 (equipped with an AxioCam camera and ZEN software). AutoQuant X deconvolution software was used to remove blur and enhance contrast and resolution. A Nikon A1 laser scanning confocal microscope (equipped with an EMCCD camera and NIS elements software) was also used for image acquisition and analysis, using either a Plan Apo λ 40x/0.95 NA or Plan Apo λ 60x/1.4 NA oil immersion objective.

EMB-9::mCherry, MIG-17::GFP, and mNG::PXN-2 total fluorescence intensities in z-projections were quantified using ImageJ. All image acquisition parameters (including exposure time, excitation intensity, and gain), for each imaging channel, were fixed across genotypes for a given fluorescent reporter. The region of interest (ROI) containing mCherry, GFP or mNG signals on the images was either outlined manually (the whole head) or defined as a rectangle. To correct for background fluorescence, four ROIs located outside but adjacent to the animal's head were used; the mean fluorescence of background ROIs was subtracted from the signal intensity for the same image, using the following formula: Corrected IntDen = IntDen of experimental ROI - (mean of background ROI X area of experimental ROI). 3D isosurface renderings of EMB-9::mCherry in *mig-6* mutants were constructed using Imaris 7.4 software (Bitplane).

Brillouin microscopy and imaging of mechanical properties. Brillouin microscopy measures tissue mechanical properties (elasticity, viscosity) in the GHz frequency range through the interaction of light with the sample's acoustic phonons [8,9]. The shift and linewidth of the Brillouin scattered light spectrum gives information about the longitudinal modulus, which is directly related to the elastic and viscous modulus of the material, respectively. Brillouin imaging was performed using a Brillouin microscope previously described [10], and animal preparation and imaging protocols previously described [8]. Briefly, a commercial Zeiss body (Axiovert 200 M) coupled with a home-built spectrometer based on a 2-VIPA configuration, provides a precision of 22 and 56 MHz for Brillouin shift and linewidth measurements, respectively, for our measurement parameters (100 ms exposure time, 4 mW optical power on the sample) and a measured spectral resolution of 520 MHz. To account for the finite spectral resolution, the latter (520 MHz) was subtracted from the measured linewidth values (subtraction corresponds to deconvolution, as detailed in Chan et al 2021). The effective NA of the objective (0.85) leads to ~100–110 MHz downshift in the Brillouin shift. A 532 nm laser (Torus, Laser Quantum) was used for Brillouin imaging. Wild-type and *mig-6* mutant animals of the L2 and L4 stages were imaged. Animals were grown in the absence of food for 2 h before experiments, to minimize variability of Brillouin signal in the pharynx due to the presence of bacteria food. Animals were anesthetized using M9 buffer containing 10 mM NaN_3 and mounted on 2% agarose pads containing 10 mM NaN_3. Brillouin images were acquired with a 40x/1.0 NA Zeiss objective and an integration time for a single point of 100 ms. The optical power on the sample was kept below 4 mW and no apparent photodamage was observed after imaging.

For Brillouin image analysis, only images of healthy animals (with no vacuoles around the bulb) were used. Also, we only analyzed images of animals that had moved very little, specifically <1.5 μm for L2 and <2.2 μm for L4 animals (the brightfield imaging acquisition preceding and following Brillouin image acquisition enabled us to measure any animal movement during imaging). Regions of interest for analysis of Brillouin microscopy images were drawn flanking the 2nd pharyngeal bulb to the animal's body edge (cuticle). Spatial maps of elasticity and viscosity are plotted from the acquired data, using function "Measure" and Lookup table mpl-Inferno in Fiji and adjusting the "Brightfield and Contrast" at 7.5–8.2 or 1.02–2.05 respectively. From the raw Brillouin shift and linewidth values we compute the Brillouin elastic and viscous contrast, analogous to Antonacci et al. 2020. Mean Brillouin linewidth, shift, and loss tangent of the ROIs were measured for each animal, averaged for each genotype, and compared between genotypes. Graphs and statistical analyses were made on raw and relative values. Relative values consist of raw values divided by the mean value obtained for the wild-type genotype.

Refractive index of animals of each genotype was measured by performing label-free 3D Holo-Tomographic Live Cell Imaging, using the Nanolive 3D Cell Explorer Fluo, as previously described [8]. Briefly, L2 or L4 animals were mounted on slides using Pluronic F127 36% w/v + 1 mM tetramisole solution. Brightfield images were acquired using a 60x/0.8 NA objective using a FITC filter of the Nanolive Module LED DAPI-FITC-TRITC/Cy5 4X B 000.

Transgenes and rescue experiments

***mig-6S* minigene** (pCB411). The *mig-6S* minigene includes exons 1-11, introns 1-4 of isoform a, and the endogenous *mig-6S* 3'UTR, which were amplified out of pZH125 [11] using primers oCBQc17 (CAAGCTCCCGGGATGAGGTTGCTGCTCTTCTCGG) and oCBQc18 (CATGATACTAGTGCGCAACAATGGGTGAAGAAAGC), adding XmaI and SpeI sites. This insert was cloned into vector PCR2.1 TOPO.

P*rgef-1::mig-6S* (pCB408). Vector backbone and promoter were obtained from plasmid pCB199 (P*rgef-1::rib-1*cDNA) by digesting with XmaI [3480] and SpeI [5422] to remove *rib-1*; the *mig-6S* insert [5625 nt] released from pCB411 by XmaI and SpeI digestion was ligated.

P*dpy-7::mig-6S* (pCB409). Vector backbone and promoter were obtained from plasmid pCB249 (P*dpy-7::lon-1a* cDNA) by digesting with XmaI [271] and SpeI [2003] to remove *lon-1*; the *mig-6S* insert [5625 nt] released from pCB411 by XmaI and SpeI digestion was ligated.

P*myo-3::mig-6S* (pCB416). Plasmid pPD95.86 (P*myo-3::unc-54* 3'UTR) was digested with XbaI [2414] and ApaI [3319] to remove the *unc-54* 3'UTR. The released vector was ligated with the *mig-6S* insert [5643 nt], which was amplified from pZH125 using primers oCBQc44 (TCGGAGTCTAGAATGAGGTTGCTGCTCTTCTCGG) and oCBQc20 (CAAGATGGGCCCGCGCAACAATGGGTGAAGAAAGC) to add on XbaI and ApaI sites, and digested.

P*myo-3::mig-6S∆papilin cassette/lagrin repeats* (pCB483). Plasmid pCB416 (P*myo-3::mig-6S*) was digested using NcoI [2715] and ClaI [7527] to remove the sequences corresponding to the end of exon 2 (from the NcoI site) until exon 11 (up to ClaI site) of *mig-6S*. This fragment was ligated with a second one to generate a plasmid that lacks the sequences coding for the papilin cassette and the lagrin repeats, but included the rest of coding sequence of *mig-6* until the Stop codon and the 3'UTR as in pCB416. The second fragment consists of most of exon 8 and exons 9, 10 and 11, which was PCR amplified from plasmid pCB416 using primers oCB2266 (CATGATCCATGGccaacttgcgttgactctg, to add an NcoI site) and oCBQc25 (GAACTTACTCGGGCATCTCG, taking advantage of an internal ClaI site) and digested, to reconstitute an in-frame *mig-6S∆papilin cassette* version by ligation.

P*myo-3::mig-6S∆Kunitz domains* (pCB492). A fragment of the *mig-6S* gene containing sequence from the initiator ATG in exon 1 all the way to exon 8, thus lacking sequence encoding the Kunitz inhibitor domains, was PCR amplified using primers oCBQc44 (tcggagtctagaatgaggttgctgctcttctcgg, containing an XbaI site) and oCB1810 (catgatAGCGCTTTAattacaggcggcgattttatg, to add an AfeI site) from plasmid pCB416 (P*myo-3::mig-6S*) and digested. This fragment was ligated with another one containing the vector backbone and promoter obtained from plasmid pCB423 (P*myo-3::sdn-1cDNA*) by digesting with XbaI and AfeI.

All inserts of finalized clones were verified by sequencing.

Transgenic animals were generated by standard microinjection techniques [12]. Plasmid pZH125, which contains a *mig-6*S minigene expressed under the *mig-6* endogenous promoter [11], was injected at a concentration of 5 ng/µL. For tissue-specific rescue assays, pCB416 [*Pmyo-3::mig-6S*] was injected at 1 ng/µL with *ceh-22::gfp* or *lgc-11::gfp* as a marker of transgenesis; pCB408 [*Prgef-1::mig-6S*] was injected at 7 ng/µL with *lgc-11::gfp* as co-injection marker; and pCB409 [*Pdpy-7::mig-6S*] was injected at 0.5 ng/µL and 0.1 ng/µL with *lgc-11::gfp* as co-injection marker. Co-injection markers were injected at 50 ng/µL and pBSK(+) was added to each mix to reach a final DNA concentration of 200 ng/µL. DNA mixes were injected into a strain where the *mig-6* gene is balanced: *sax-7(qv30); mig-6(qv33)/dpy-11(e224) oyIs14*. Transgenic strains are listed in Table S4.

Liquid cultures. After hatching into L1 larvae, animals were grown in liquid culture for up to 5 days, kept under constant agitation on a nutator for them to swim continuously. Synchronized L1 larvae were obtained by treating gravid adults with a bleach solution [13], resuspending the collected embryos in 2 mL S medium in 15 mL conical tubes, and incubating them overnight at 20°C on a nutator to allow their hatching into L1 larvae that are arrested in the absence of food. The L1 larvae culture was then supplemented with 200 µL of concentrated *E. coli* OP50 and kept under constant agitation on a nutator at 20°C for 5 days, with additional concentrated *E. coli* OP50 added at days 1 and 3 post hatching. Animals were examined by microscopy at 3 and/or 5 days after hatching (post-L1 days 3 or 5 is equivalent to the age of young or 2-day-old adults of animals grown on solid plates, respectively).

RNA interference assays. RNAi experiments were performed by the feeding method using RNAi bacterial clones from the Ahringer RNAi library [14,15]. Single colonies for the L4440 empty vector negative control, and for clones containing *emb-9* and *pxn*-2 sequences, were obtained on LB plates with ampicillin (75 µg/mL) and tetracycline (12.5 µg/mL). They were then grown in LB medium with ampicillin (75 µg/mL) for 16h at 37°C, to which 1 mM IPTG was then added and incubated for an additional hour to induce dsRNA expression. Concentrated cultures of these RNAi bacteria were seeded onto NGM plates (75 µg/mL Amp, 1 mM IPTG) and left to dry at room temperature for overnight induction. For *mig-6*(RNAi) experiments, *sax-7(qv30)* animals were placed on RNAi plates at the L2 stage and examined as day 2 adults (48 hours post L4). For *emb-9*(RNAi) and *pxn-2*(RNAi), synchronized L1 larvae [13] were distributed onto RNAi plates, incubated at 20°C, and examined as day 2 adults (48 hours post L4).

For RNAi assays done with animals swimming in liquid (**Fig. 7L**), worms were synchronized as L1 larvae and then grown in liquid media as described above, except that they were fed with HT115 RNAi bacteria (carrying the control vector or the *emb-9*(RNAi) plasmid) in S medium supplemented with ampicillin (75 µg/mL) and IPTG (1 mM). 250 µL of concentrated bacteria was added to the liquid cultures initially (after hatching), and then at day 3 post L1. Optimal induction of dsRNA expression by the RNAi bacteria was achieved by growing bacteria for 14 hours at 37°C, and then adding 5 mM IPTG for growth for another 4 hours. Animals were recovered from the liquid culture and examined at day 5 post L1.

Statistical analyses. All statistical analyses were performed using R (version 4.1.2) using the R Stats package ('stats' version 4.4.2). Data are presented as mean ± standard error of proportion, or as mean ± standard error of the mean. As indicated in each Figure Legend, statistical tests were performed using z-test, unpaired two-tailed Student's t-test, or one-way ANOVA when applicable (for parametric datasets). For non-parametric datasets, the Wilcoxon-Mann-Whitney test was used. Appropriate *post-hoc* tests were performed for multiple comparisons: Bonferroni correction was applied after z-tests and Wilcoxon-Mann-Whitney tests, while Tukey HSD correction was applied following ANOVA. All sample sizes and raw data are available in **Supplementary Information**.

REFERENCES

1. Brenner, S. The genetics of Caenorhabditis elegans. *Genetics* **77**, 71-94 (1974).
2. Beanan, M. J. & Strome, S. Characterization of a germ-line proliferation mutation in C. elegans. *Development* **116**, 755-766 (1992).
3. Doitsidou, M., Jarriault, S. & Poole, R. J. Next-generation sequencing-based approaches for mutation mapping and identification in Caenorhabditis elegans. *Genetics* **204**, 451-474 (2016).
4. Minevich, G., Park, D. S., Blankenberg, D., Poole, R. J. & Hobert, O. CloudMap: a cloud-based pipeline for analysis of mutant genome sequences. *Genetics* **192**, 1249-1269 (2012).
5. Arribere, J. A., Bell, R. T., Fu, B. X., Artiles, K. L., Hartman, P. S. & Fire, A. Z. Efficient marker-free recovery of custom genetic modifications with CRISPR/Cas9 in Caenorhabditis elegans. *Genetics* **198**, 837-846 (2014).
6. Dickinson, D. J., Ward, J. D., Reiner, D. J. & Goldstein, B. Engineering the Caenorhabditis elegans genome using Cas9-triggered homologous recombination. *Nature methods* **10**, 1028-1034 (2013).
7. Bénard, C. Y., Boyanov, A., Hall, D. H. & Hobert, O. DIG-1, a novel giant protein, non-autonomously mediates maintenance of nervous system architecture. (2006).
8. Coraggio, F. *et al.* Age-progressive interplay of HSP-proteostasis, ECM-cell junctions and biomechanics ensures C. elegans astroglial architecture. *Nature Communications* **15**, 2861 (2024).
9. Prevedel, R., Diz-Muñoz, A., Ruocco, G. & Antonacci, G. Brillouin microscopy: an emerging tool for mechanobiology. *Nature methods* **16**, 969-977 (2019).
10. Bevilacqua, C., Sánchez-Iranzo, H., Richter, D., Diz-Muñoz, A. & Prevedel, R. Imaging mechanical properties of sub-micron ECM in live zebrafish using Brillouin microscopy. *Biomedical Optics Express* **10**, 1420-1431 (2019).
11. Kawano, T. *et al.* C. elegans mig-6 encodes papilin isoforms that affect distinct aspects of DTC migration, and interacts genetically with mig-17 and collagen IV. (2009).
12. Mello, C. & Fire, A. DNA transformation. *Methods in cell biology* **48**, 451-482 (1995).
13. Stiernagle, T. Maintenance of C. elegans. *WormBook: The online review of C. elegans biology [Internet]* (2006).
14. Kamath, R. S. & Ahringer, J. Genome-wide RNAi screening in Caenorhabditis elegans. *Methods* **30**, 313-321 (2003).
15. Timmons, L. & Fire, A. Specific interference by ingested dsRNA. *Nature* **395**, 854-854 (1998).

Figure S1

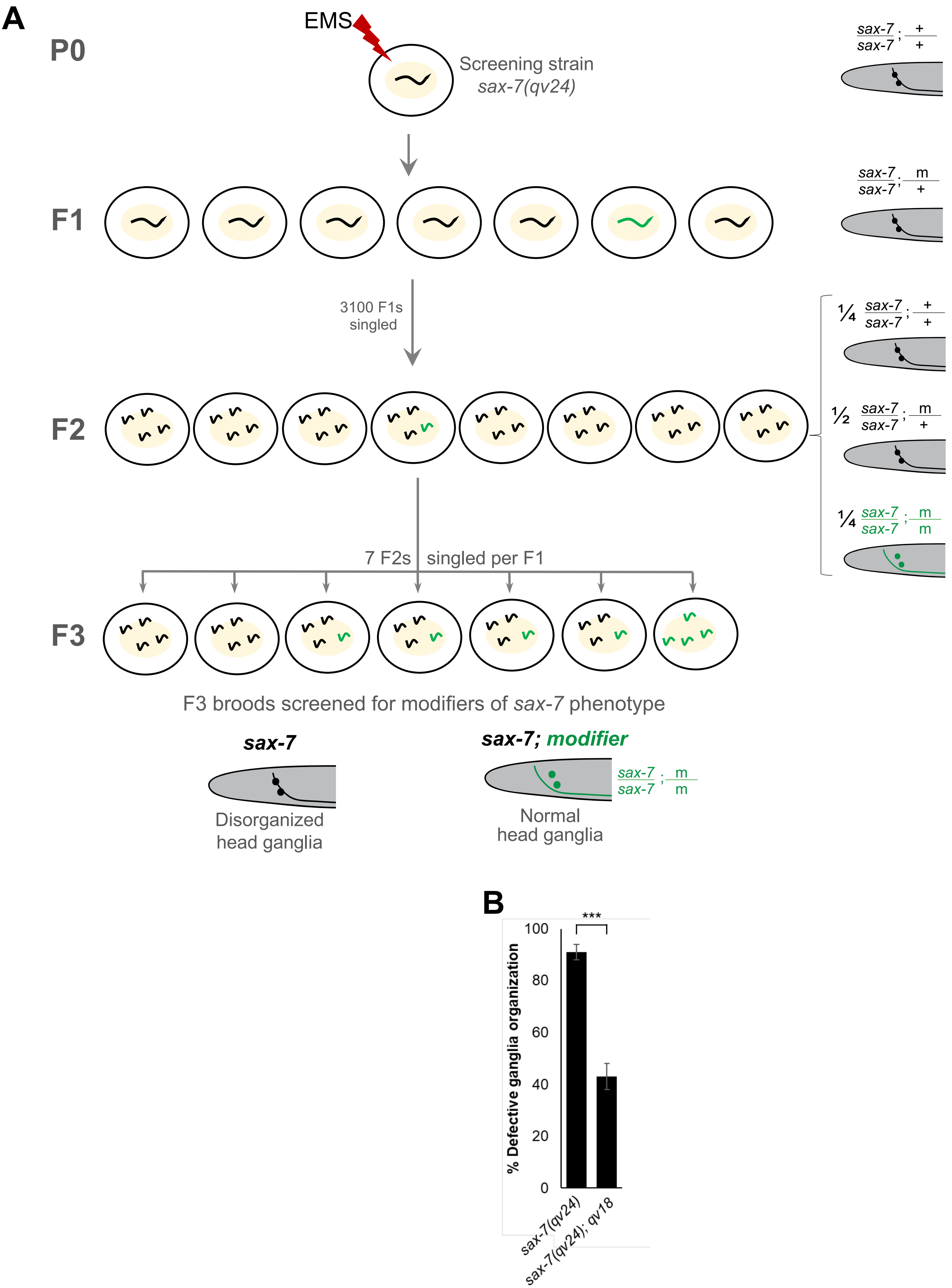
A
P0
EMS
Screening strain
sax-7(qv24)
F1
3100 F1s
singled
F2
7 F2s singled per F1
F3
F3 broods screened for modifiers of sax-7 phenotype
sax-7
Disorganized
head ganglia
sax-7; modifier
Normal
head ganglia
¼
½
¼
B
***
% Defective ganglia organization
100
80
60
40
20
0
sax-7(qv24)
sax-7(qv24); qv18

Figure S1. Forward F2 clonal genetic screen to identify novel neuronal maintenance factors by searching for modifiers of the defective head neuronal organization of *sax-7* mutants.

(**A**) Schematic of the forward genetic screen carried out to identify novel neuronal maintenance factors. P0 *sax-7(qv24)* L4 worms were mutagenized with ethyl methanesulfonate (EMS). 3 100 F1 progeny of the mutagenized P0s were picked singly onto new plates; rare animals containing a potential modifier mutation of the *sax-7* head neuronal disorganization phenotype would be heterozygous at this generation and visible only if the mutation were dominant and highly penetrant. Thus, for each F1, 7 F2s were picked singly onto new plates. If an F2 animal was heterozygous for a modifier mutation, then one quarter of its brood (F3) would be homozygous for the modifier mutation (represented in green). If an F2 animal was homozygous for a modifier mutation, its F3 brood would be homozygous for the modifier mutation. Broods of F3 adult animals were screened by fluorescence microscopy on a stereoscope to find those where a large proportion of animals displayed a modified head ganglia organization phenotype compared to non-mutagenized *sax-7(qv24)* adults. (**B**) Quantification of chemosensory neurons ASH and ASI disorganization in 2-day-old adults of *sax-7(qv24)* and double mutant animals *sax-7(qv24); qv18*, which were less defective compared to *sax-7* mutants, indicating that the newly isolated mutation *qv18* supresses the neuronal defects of *sax-7* mutants. As a note, the molecular lesion present in allele *mig-6*(*qv33)* used in this report is identical to that of *qv18* as *qv33* was CRISPR-Cas9 generated to introduce the mutation *qv18* in a clean background. Error bars are the standard error of the proportion. Asterisks denote significant difference: ****P* ≤ 0.001 (z-test). Sample sizes and data in **Supplementary Information**.

Figure S2

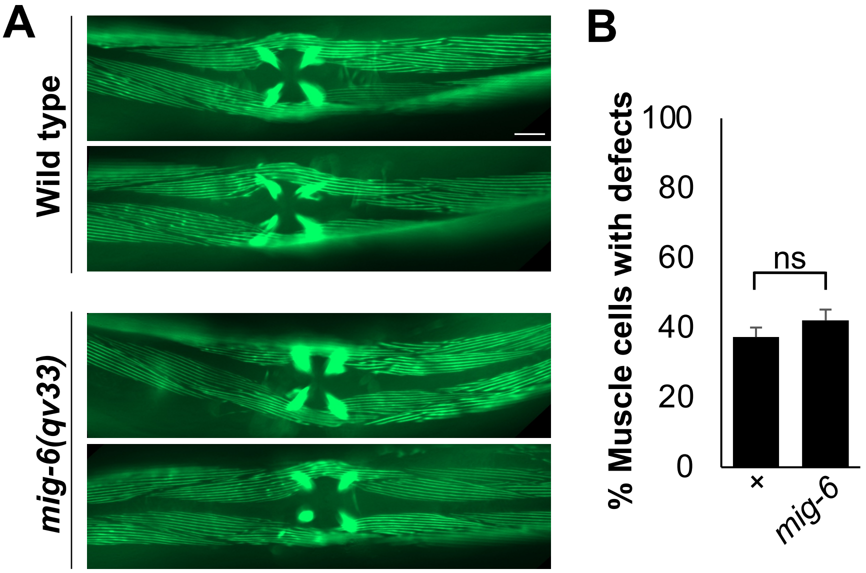

Figure S2. *mig-6* mutants have normal body wall muscle structure.
(**A**) Images of body wall muscles in the central region of the animal's body in wild-type and *mig-6(qv33)* mutant 1-day-old adults visualized with the *stEx30* P*myo-3*::GFP::MYO-3 reporter. Muscles of the vulva are also visible (center of images). (**B**) Quantification of body wall muscle phenotype (sarcomere organization) in wild type and *mig-6* mutants. Error bars are the standard error of the proportion. Sample sizes and data in Supplementary Information. “+” indicates wild-type strain; n.s., not significant (z-test). Scale bar, 20 μm.

Figure S3

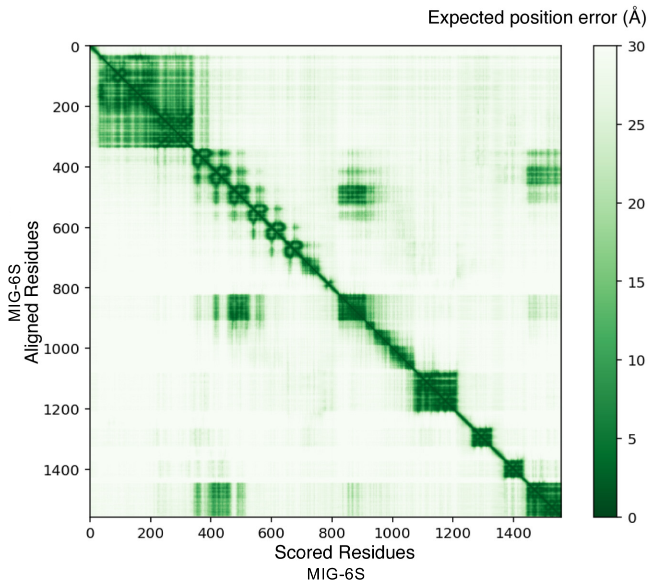

Figure S3. ColabFold 1.5.2-related information on MIG-6S.
Predicted aligned errors (PAE) corresponding to the predicted structure of MIG-6S shown on Fig. 1D". This PAE analysis shows regions of high confidence (dark green) and low confidence (pale green) in relative domain positioning.

Figure S4

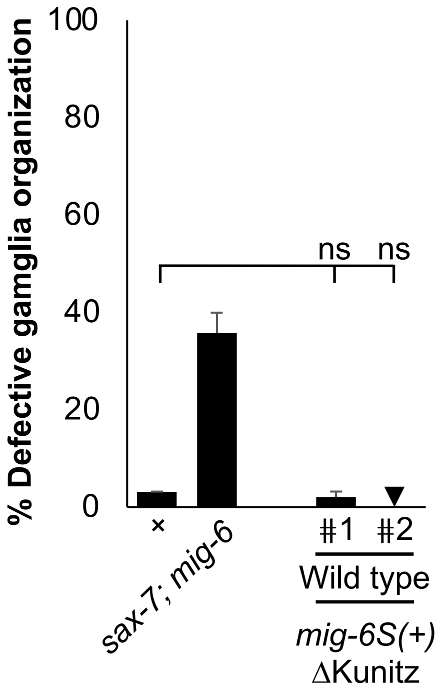

Figure S4. Control assays using a recombinant transgene of *mig-6S* lacking the Kunitz domains in wild-type animals.

Two independent transgenic lines overexpressing *mig-6S* lacking the Kunitz domains display normal neuronal organization, indicating that overexpression of this transgene does not lead to neuronal defects. Thus, the restoration of neuronal defects in *sax-7; mig-6* double mutants expressing this transgene (**Fig. 1E**) is the result of this transgene's rescuing activity. Error bars are the standard error of the proportion. Sample sizes and data in Supplementary Information. "+" indicates wild-type strain; n.s., not significant (z-test).

Figure S5

A

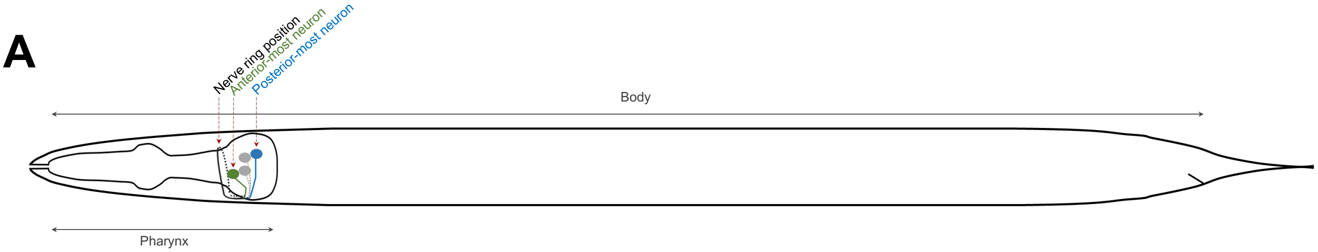

B

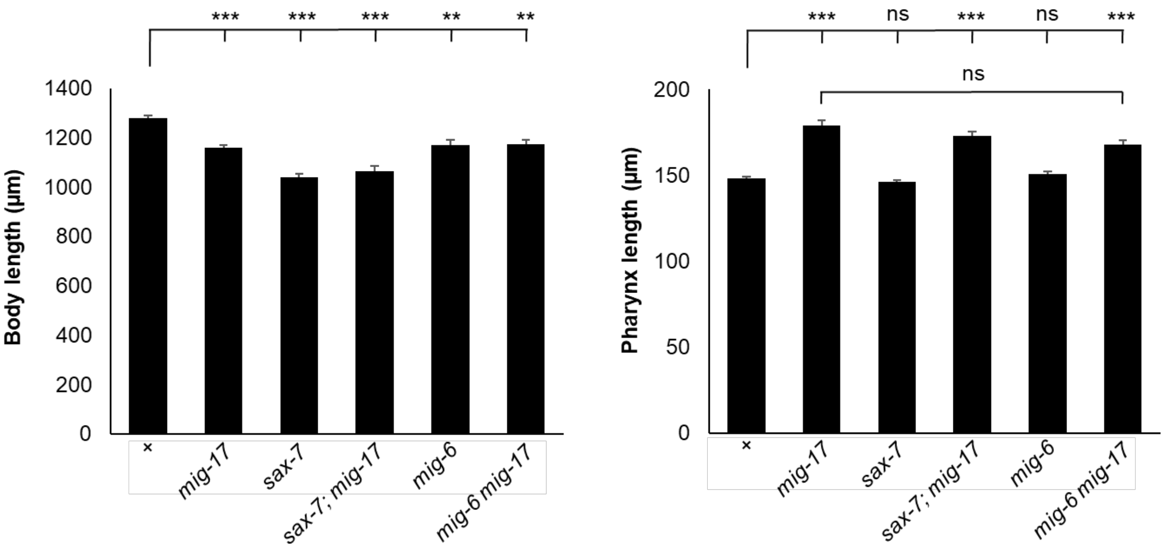

C

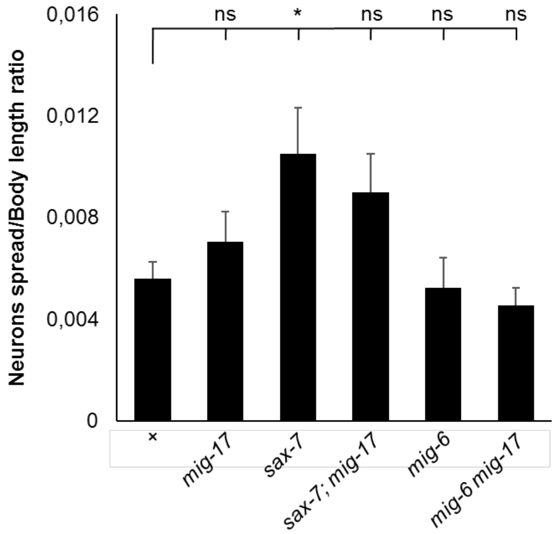

D

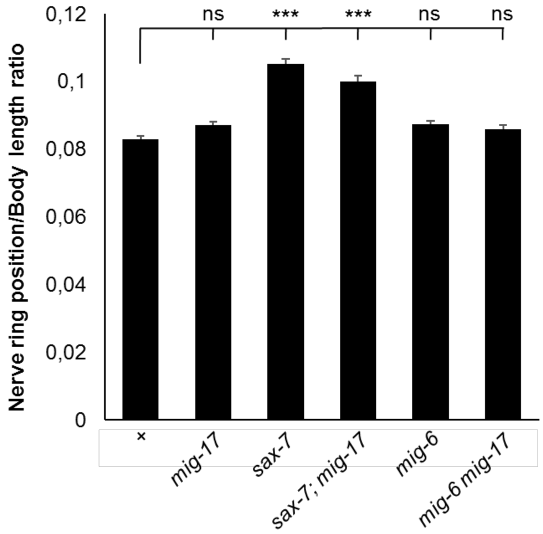

Figure S5. The longer pharynx of *mig-17* mutants does not affect the position of ASH and ASI soma and of the nerve ring relative to body length.

We measured the distribution of neuronal soma positions and the position of the nerve ring, and calculated ratios of neuronal position to body length. (**A**) Schematic diagram of *C. elegans* hermaphrodite with four chemosensory neurons ASI and ASH drawn, left and right for each pair (as would be visualized using reporter *hdIs26* P*sra-6::DsRed2*). The most anterior soma of the four neurons, the most posterior soma of the four neurons, and the nerve ring are indicated. Body length was measured as the distance from the mouth opening to the anus, and pharynx length was measured as the distance between the mouth opening and the posterior edge of the terminal bulb. (**B**) Analysis of body and pharynx lengths of 2-day-old wild-type animals and mutants *mig-17*, *mig-6* and *sax-7*, as well as single or combined mutant animals of *mig-17* with *sax-7* or *mig-6* mutations. (**C**) Quantification of the ratio between the spread of neuronal positions (distance between the position of the anterior most neuron to the posterior most neuron in each animal) and the body length of 2-day-old animals. The higher the ratio, the more posterior the neuronal structures are. For example, because of head ganglia defects in *sax-7* mutants, the ratios appear higher in these mutants. (**D**) Quantification of the ratio between the nerve ring position and the body length in 2-day-old animals. The position of head neuronal structures, including soma and nerve ring, does not depend on pharynx length, being in a conserved position relative to body length. Thus, in *mig-17* mutants, despite their longer pharynx, there is no difference in neuronal position of their somas and nerve ring compared to the wild type. Error bars are the standard error of the mean. Asterisks denote significant difference: $^{*}P \leq 0.05$, $^{***}P \leq 0.001$ (Wilcoxon Mann-Whitney test for body length and ANOVA for pharynx length in B; ANOVA in C and D); *P*-values were corrected by multiplying by the number of comparisons, Bonferroni correction; sample sizes and data in **Supplementary Information**). “+” indicates wild-type strain; n.s., not significant.

Figure S6

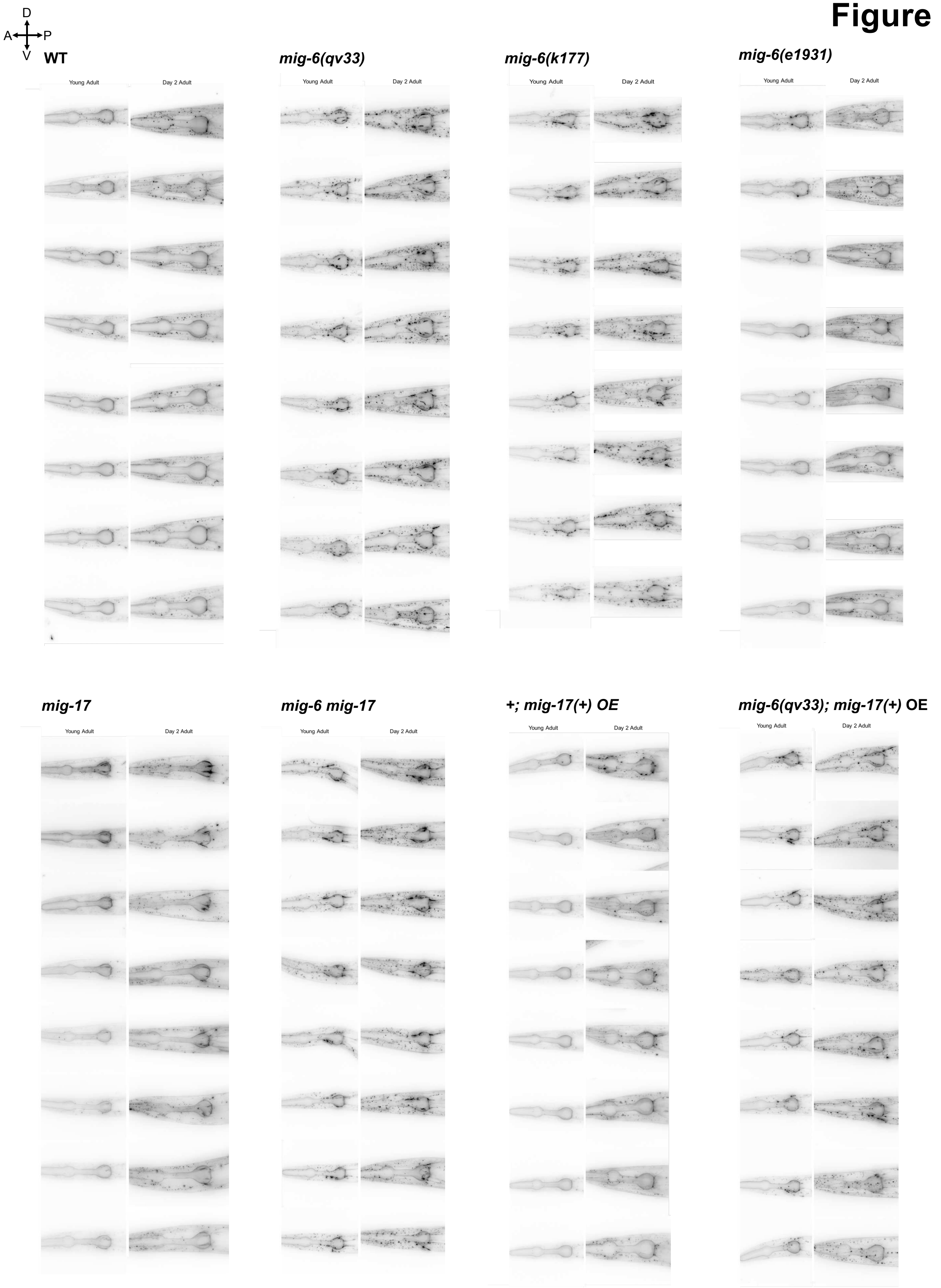

D
A
P
V
WT
Young Adult
Day 2 Adult
mig-6(qv33)
Young Adult
Day 2 Adult
mig-6(k177)
Young Adult
Day 2 Adult
mig-6(e1931)
Young Adult
Day 2 Adult
mig-17
Young Adult
Day 2 Adult
mig-6 mig-17
Young Adult
Day 2 Adult
+; mig-17(+) OE
Young Adult
Day 2 Adult
mig-6(qv33); mig-17(+) OE
Young Adult
Day 2 Adult

Figure S6

(continued)

mig-6(qv33)/+

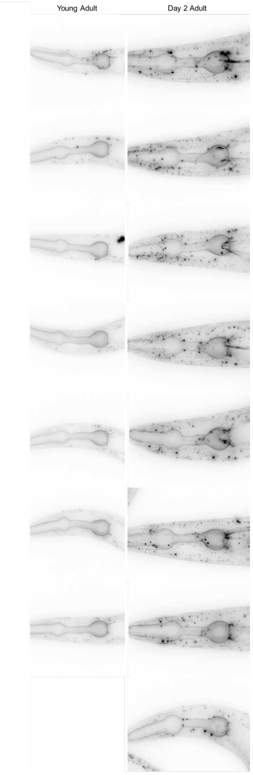

mig-6 mig-17/++

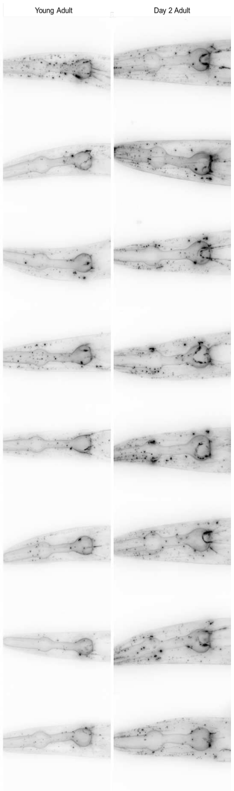

sax-7

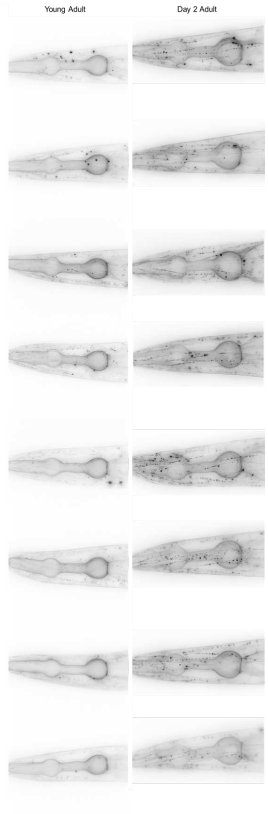

sax-7; mig-6(qv33)

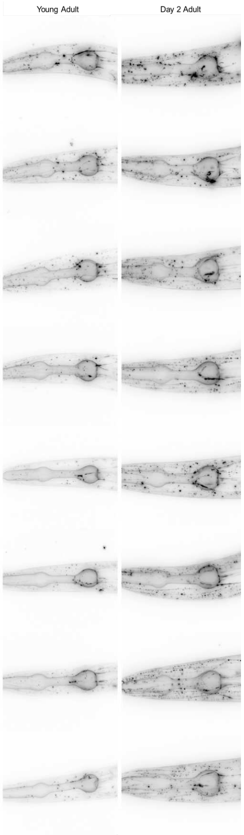

sax-7; mig-6(k177)

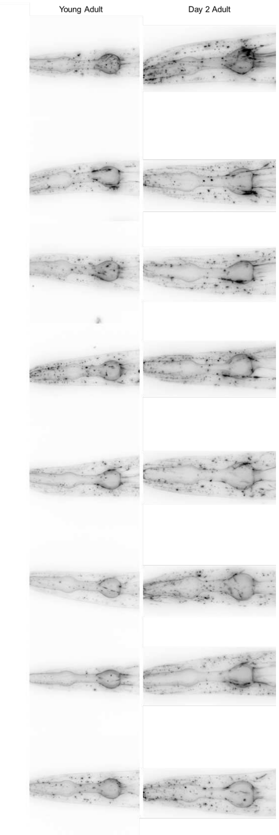

sax-7; mig-17

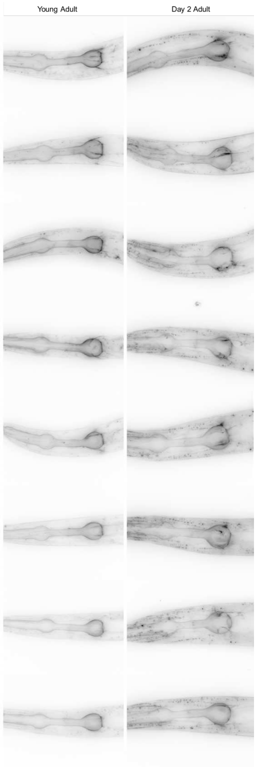

Figure S6. Distribution of collagen IV in different mutants.

Panels of fluorescence images (sum projection) of EMB-9::mCherry (*qyIs46* P*emb-9::emb-9::mCherry*) for 2-day-old adults of genotypes analyzed in this study. The robustness of the fibrotic-like structures accumulation phenotype of *mig-6* and *mig-17* single and combined mutants can be appreciated, as well as the range of their positions in the head (coinciding mostly with the region around terminal bulb of the pharynx).

Figure S7

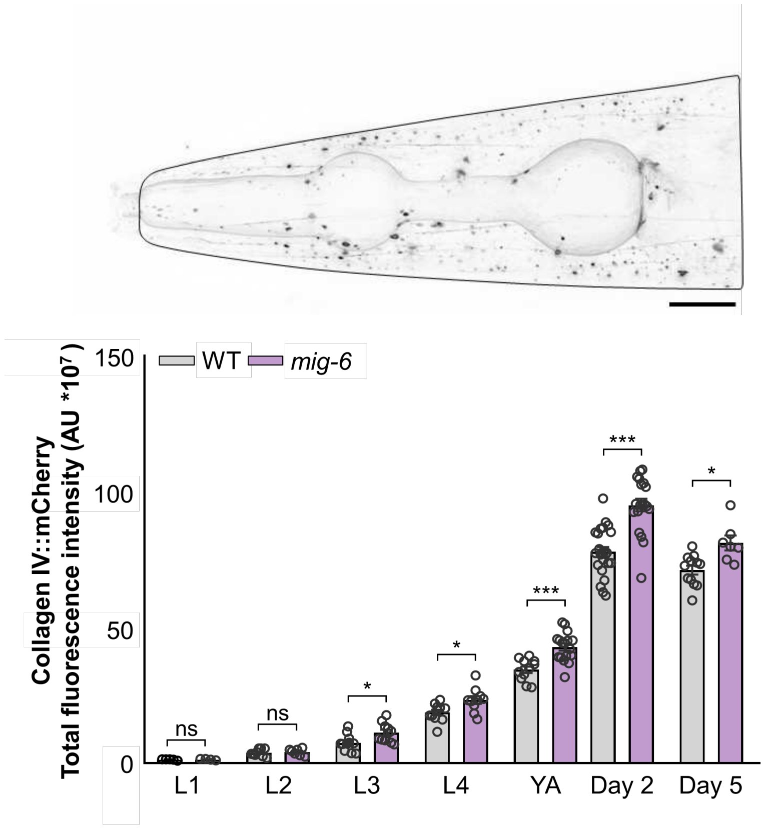

Figure S7. Collagen IV levels increase with age in *mig-6* mutant animals.
Quantification of fluorescence intensity of EMB-9::mCherry (*qyIs46* P*emb-9::emb-9::mCherry*) in the head region at larval stages (L1, L2, L3, and L4), and adult ages (young adult, 2- and 5-day-old adult animals) in control animals and *mig-6(qv33)* mutants. ROI was drawn from the mouth opening to a position posterior to terminal bulb by 20% of the pharynx length. A.U., arbitrary units. Scale bar, 20 µm. Error bars are the standard error of the mean. Asterisks denote significant difference: *$P \leq 0.05$, ***$P \leq 0.001$ (Wilcoxon test to compare data of the first larval stage L1 between WT and *mig-6* mutants and t-test for the other stages); *P*-values were corrected by multiplying by the number of comparisons, Bonferroni correction; sample sizes and data in **Supplementary Information**). n.s., not significant.

Figure S8

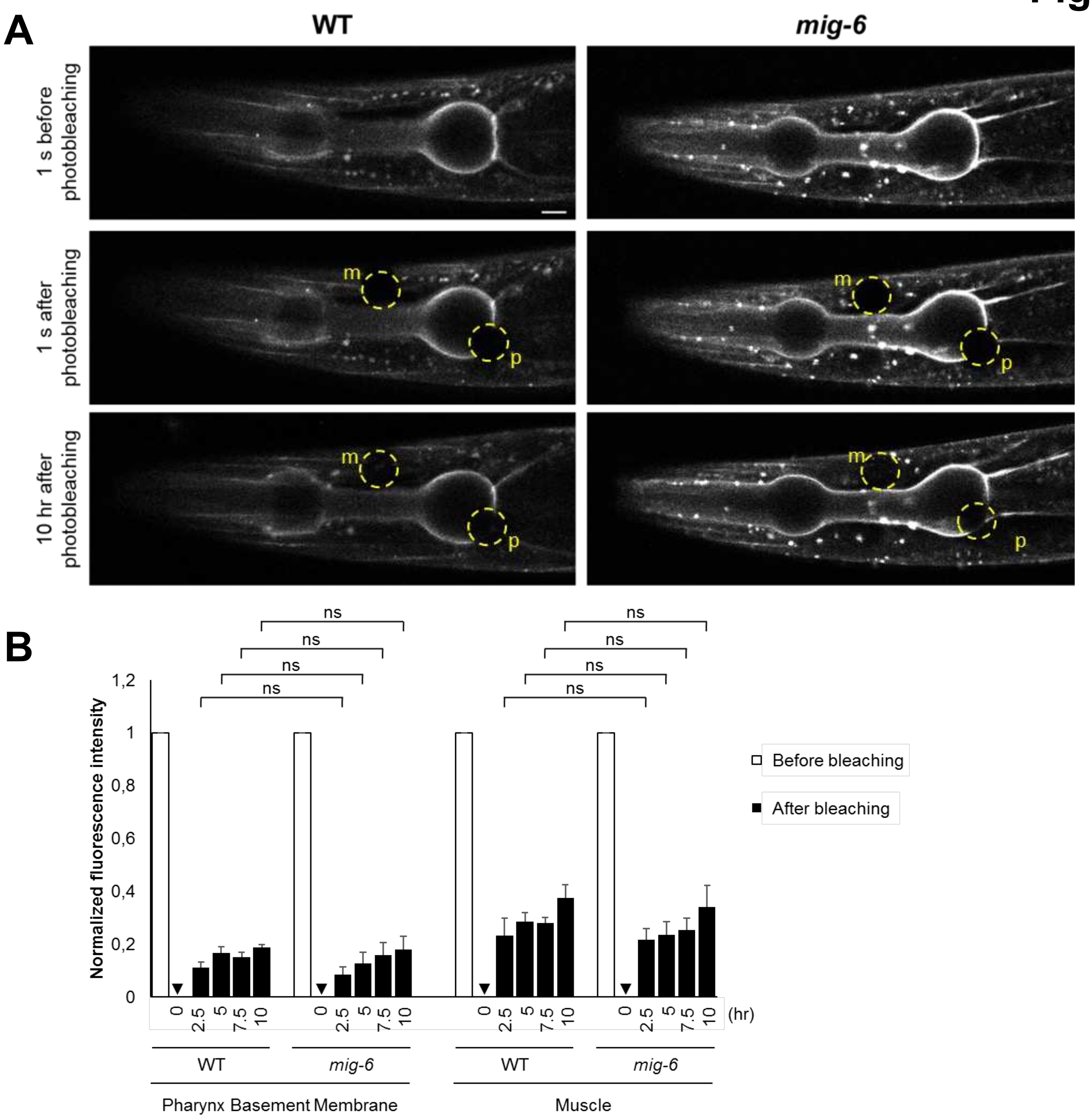

Figure S8. Collagen IV stability in the basement membrane is not affected by *mig-6* loss of function.

We used fluorescence recovery after photobleaching (FRAP) analysis to assess EMB-9::mCherry fluorescence recovery in wild-type and *mig-6* mutants. (**A**) Images show a single confocal z slice of the head region. ROI of photobleached regions is indicated by circles (m for muscle and p for pharynx). A control region located outside of the animal is also used (not shown). (**B**) Quantification of normalized fluorescence recovery in wild-type and mutant *mig-6* young adults, in pharynx basement membrane and in muscle after 0, 2.5, 5, 7.5, and 10 hours. After photobleaching a specific region of EMB-9::mCherry in muscle and pharyngeal basement membrane, we measured FRAP and normalized the data against an unbleached control region. n.s., not significant (t-test). sample sizes and data in **Supplementary Information**).

Figure S9

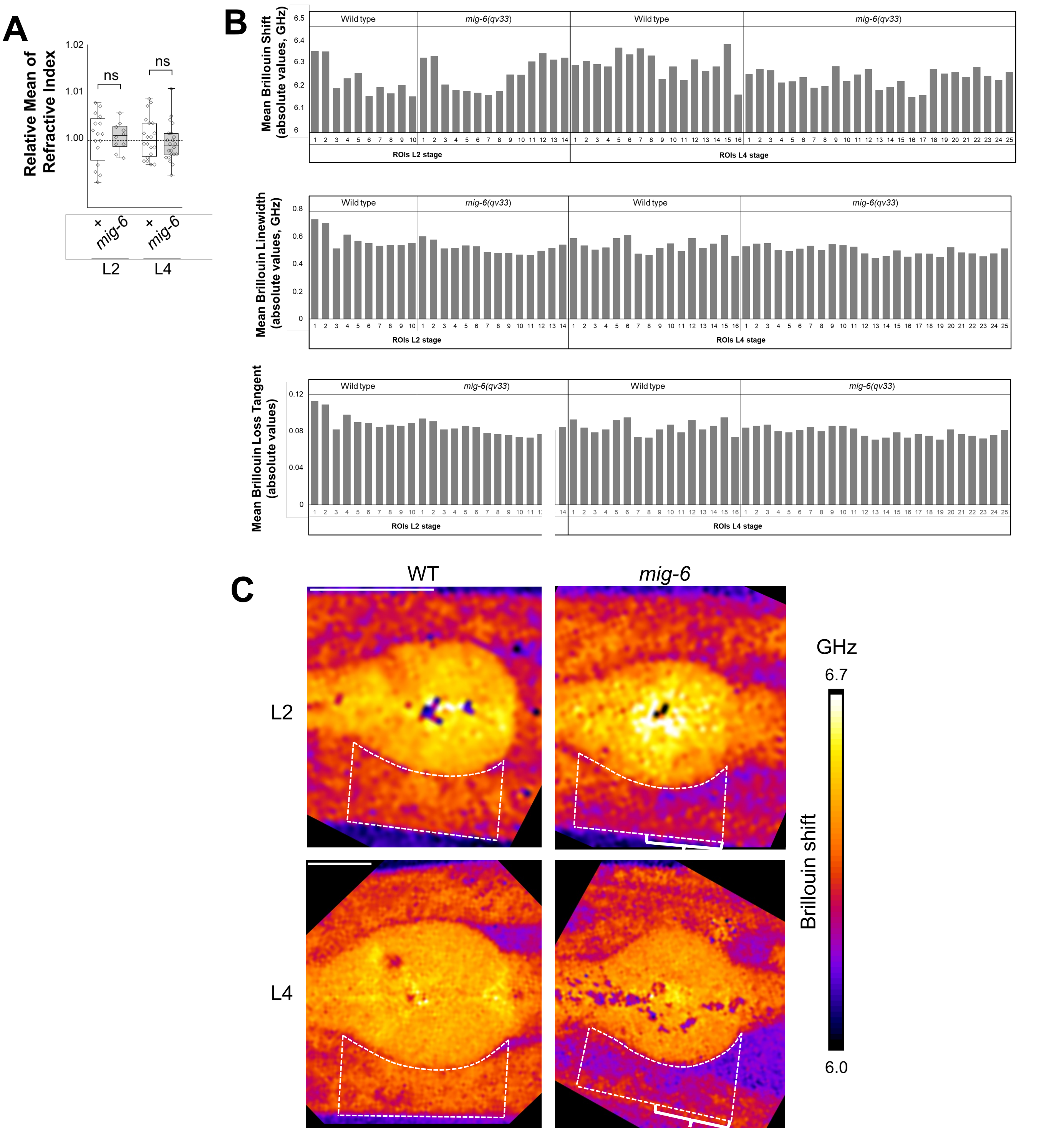

Stage	Genotype	Total number of ROIs	% ROIs with lower shift in posterior zone
L2	Wild type	10	0
	mig-6(qv33)	14	28.6
L4	Wild type	16	18.8
	mig-6(qv33)	25	92.0

Figure S9. Refractive index measurements, as well as Brillouin shift, linewidth, and loss tangent quantifications, in wild-type and *mig-6* mutant animals.

(**A**) Absolute values of the refractive index of ROIs measured in wild-type and *mig-6* mutant animals are not significantly different. Error bars are the standard error of the mean; n.s., not significant (t-test); sample sizes and data in Supplementary Information). (**B**) Quantification of Brillouin shift, linewidth, and loss tangent in each ROI in wild-type and *mig-6* mutant animals at L2 and L4 stages. (**C**) The posterior zone of the head region around the pharynx (indicated by brackets) shows less elasticity in *mig-6* mutants. Brillouin shift images of wild-type and *mig-6* mutant animals at L2 and L4 stages; scale bar, 20 µm. Table provides the quantification of the percentage of ROIs with a lower Brillouin shift in this posterior zone. Source data in **Supplementary Information**).

Figure S10

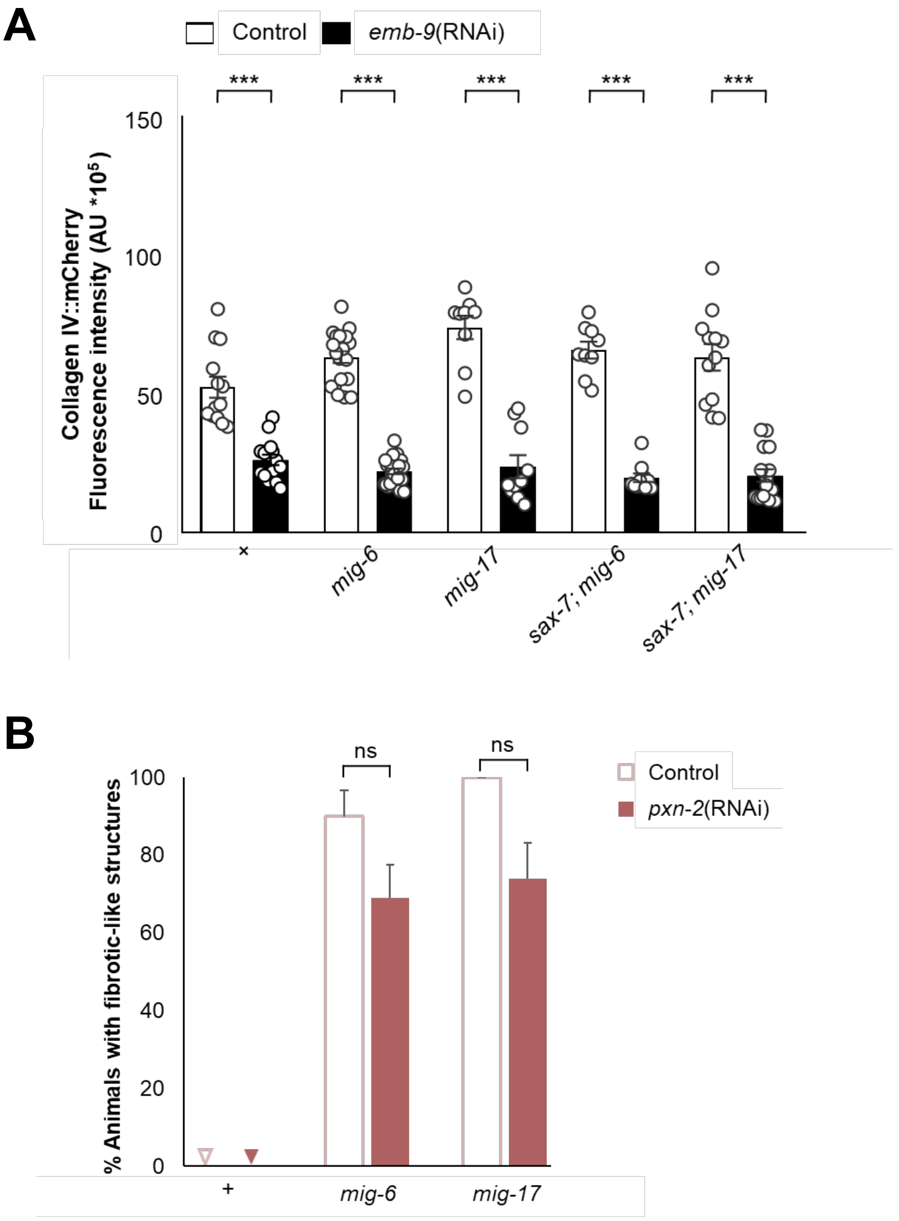

Figure S10. *emb-9*(RNAi) treatment decreases collagen IV levels as expected, and *pxn-2*(RNAi) treatment does not significantly affect the penetrance of the fibrotic phenotype.
(**A**) Quantification of fluorescence intensity of EMB-9::mCherry in the head region in 2-day-old adult animals, which were subjected to control (empty vector) or *emb-9*(RNAi), indicating efficient collagen IV depletion. Same ROI as for **Fig. S6**. A.U., arbitrary units. Error bars are the standard error of the mean. Asterisks denote significant difference: $*P \leq 0.05$, $***P \leq 0.001$ (Wilcoxon Mann-Whitney test); *P*-values were corrected by multiplying by the number of comparisons, Bonferroni correction; sample sizes and data in Supplementary Information). n.s., not significant. (**B**) Quantification of percentage of 2-day-old animals displaying fibrotic-like structures in adult animals, which were subjected to control (empty vector) or *pxn-2*(RNAi). *pxn-2* depletion does not significantly decrease the percentage of animals displaying fibrotic-like structures (however, the number and continuity of these fibrotic-like structures are profoundly affected by *pxn-2*(RNAi), see **Fig. 6 E, F, G**). n.s., not significant (z-test). Sample sizes and data in **Supplementary Information**).

Figure S11

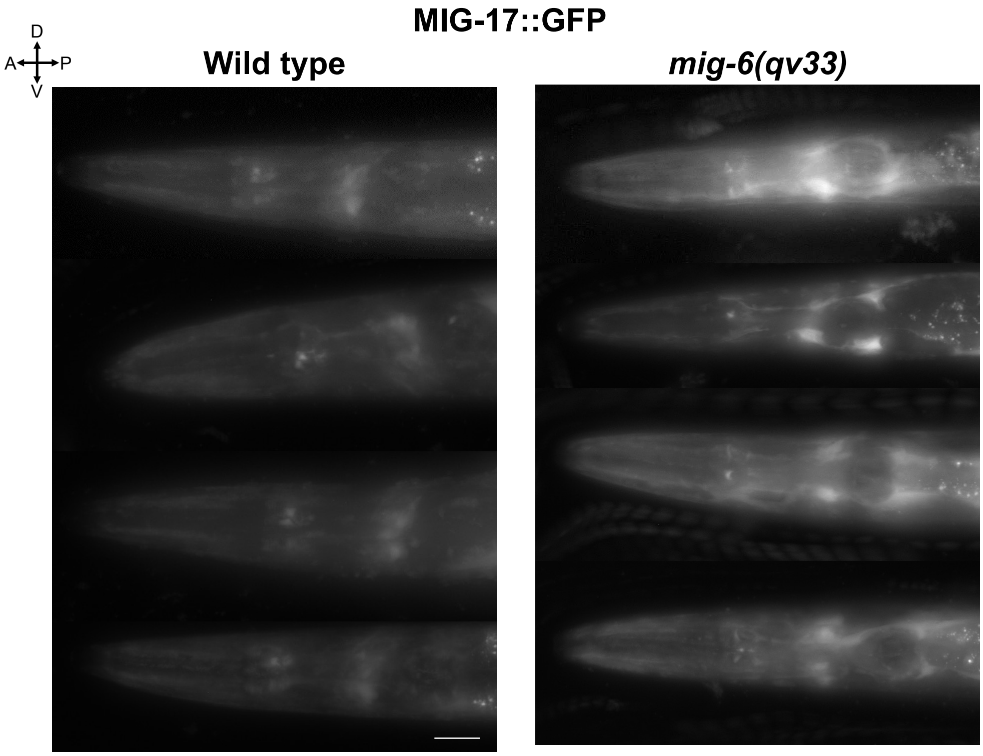

Figure S11. MIG-17/ADAMTS is upregulated in *mig-6* mutants.
Representative fluorescence images of wild-type and mutant *mig-6(qv33)* young adults showing the level and the distribution of MIG-17::GFP using reporter *evIs213* P*mig-17::gfp*. ROI was as in **Fig. 5D**. Scale bar, 20 µm.

Figure S12

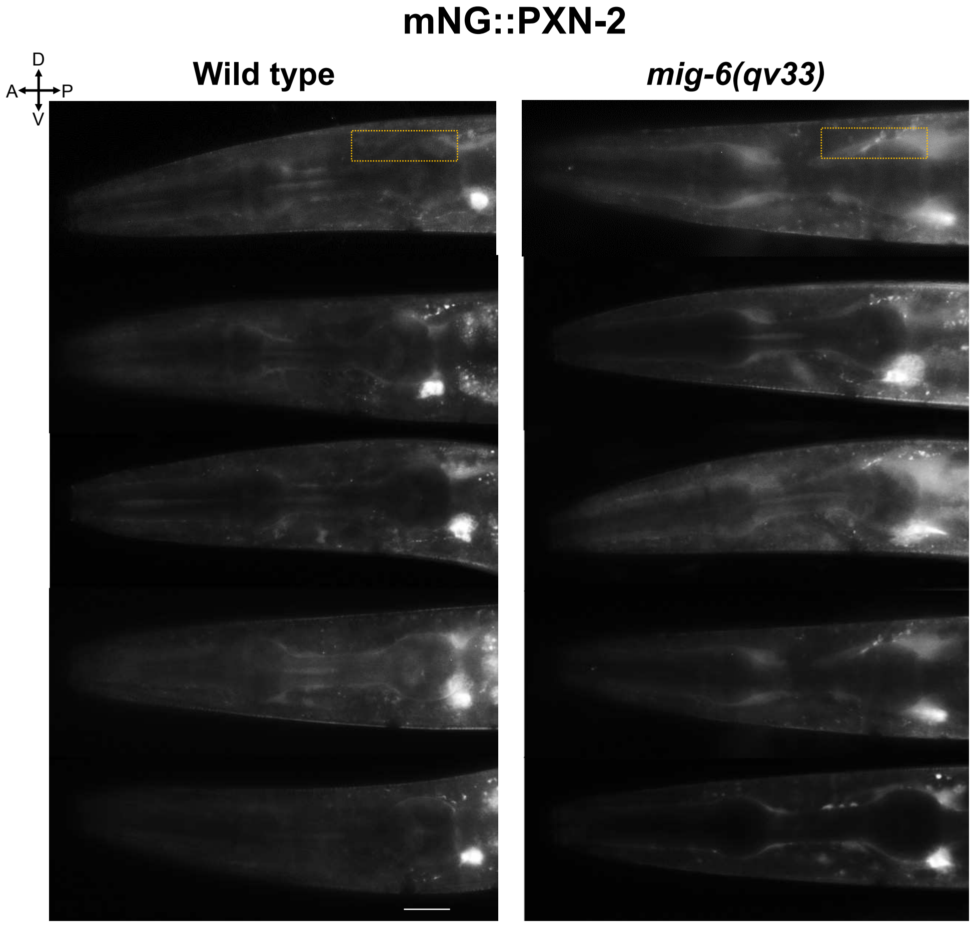

Figure S12. PXN-2 is upregulated in *mig-6* mutants.
Representative fluorescence images of wild-type and *mig-6(qv33)* mutant young adults showing the level and distribution of mNG::PXN-2 using reporter *qy76 mNeonGreen::pxn-2 N-term*. The orange rectangle indicates the ROI used to quantify fluorescence intensity in Fig. 6D. Scale bar, 20 µm.

S1 Table. List of strains used.

Strain	Genotype	Transgene	Reference
N2			[1]
VH648	*hdIs26 III*	P*odr-2::cfp;* P*sra-6::DsRed2*	[2]
VQ51	*oyIs14 V*	P*sra-6::gfp; lin-15(+)*	[3], further outcrossed for this study
VQ1253	*evIs213*	P*mig-17::mig-17::gfp; unc119(+)*	[4]
NK364	*unc-119(ed3) III; qyIs46 X*	P*emb-9::emb-9::mCherry; unc-119(+)*	[5]
VQ1485	*qyIs161*	P*emb-9::emb-9::Dendra + unc-119(+)*	[5], further outcrossed for this study
NK2326	*emb-9(qy24[emb-9::mNG+loxP]) III*	*emb-9::mNG+loxP* knock in	[6]
DE60	*dnIs13; him-5(e1490) V*	P*gly-18::gfp; unc-119(+)*	[7]
NK2565	*qy76 X*	*mNeonGreen::pxn-2*	[8]
VQ2147	*stEx30*	P*myo-3::GFP::myo-3 + rol-6(su1006)]*	[9]
**Neuronal phenotype characterization**			
VQ307	*sax-7(qv24) IV; oyIs14 V*		This study
VQ373	*sax-7(qv24) IV; qv18 oyIs14 V*		This study
VQ1061	*sax-7(qv30) IV; hdIs26 III*		This study
VQ1076	*mig-6(qv33) V; hdIs26 III*		This study
VQ1077	*sax-7(qv30) IV; mig-6(qv33) V; hdIs26 III*		This study
VQ1138	*sax-7(qv30) IV; mig-6(qv33) V; hdIs26 III; qvEx336*	P*mig-6::mig-6S*	This study
VQ1139	*sax-7(qv30) IV; mig-6(qv33) V; hdIs26 III; qvEx337*	P*mig-6::mig-6S*	This study
VQ1152	*mig-6(k177) V; hdIs26 III*		This study
VQ1154	*mig-6(ev700) V; hdIs26 III*		This study
VQ1074	*mig-6(ev701) V; hdIs26 III*		This study
VQ1070	*mig-6(sa580) V; hdIs26 III*		This study
VQ1130	*dpy-11(e224) oyIs14 V*		This study
VQ1178	*mig-6(e1931)/dpy-11(e224) oyIs14 V; hdIs26 III*		This study
VQ1153	*sax-7(qv30) IV; mig-6(k177) V; hdIs26 III*		This study
VQ1155	*sax-7(qv30) IV; mig-6(ev700) V; hdIs26 III*		This study
VQ1075	*sax-7(qv30) IV; mig-6(ev701) V; hdIs26 III*		This study
VQ1069	*sax-7(qv30) IV; mig-6(sa580) V; hdIs26 III*		This study
VQ1213	*sax-7(qv30) IV; mig-6(e1931)/dpy-11(e224) oyIs14 V; hdIs26 III*		This study
OH8608	*zig-3(tm924) zig-4(gk34) X; hdIs26 III*		[8]
VQ1195	*mig-6(qv33) V; zig-3(tm924) zig-4(gk34) X; hdIs26 III*		This study
VQ1169	*dig-1(n1321) hdIs26 III*		This study
VQ1231	*mig-6(qv33) V; dig-1(n1321) hdIs26 III*		This study
VQ2379	*dig-1(ky188) hdIs26 III*		This study

VQ2443	*dig-1(ky188) hdIs26 III; mig-6(qv33)/ dpy-11(e224) oyIs14 V*		This study
VQ1251	*mig-17(k174) V; hdIs26 III*		This study
VQ1256	*sax-7(qv30) IV; mig-17(k174); hdIs26 III*		This study
VQ1309	*mig-6(qv33) mig-17(k174) V; hdIs26 III*		This study
VQ1328	*sax-7(qv30); mig-6(qv33) mig-17(k174) V; hdIs26 III*		This study
VQ1252	*mig-17(k174)/dpy-11(e224) oyIs14 V; hdIs26 III*		This study
VQ1717	*sax-7(qv30) IV; dpy-11(e224) oyIs14/+ V; hdIs26 III*		This study
VQ1190	*sax-7(qv30) IV; mig-6(qv33)/dpy-11(e224) oyIs14 V; hdIs26 III*		This study
VQ1257	*sax-7(qv30) IV; mig-17(k174)/dpy-11(e224) oyIs14 V; hdIs26 III*		This study
VQ1687	*sax-7(qv30) IV; mig-6(qv33) mig-17(k174)/dpy-11(e224) oyIs14 V; hdIs26 III*		This study
VQ1235	*adt-2(wk156) X; hdIs26 III*		This study
VQ1243	*sax-7(qv30) IV; adt-2(wk156) X; hdIs26 III*		This study
VQ1177	*mig-6(qv33) V; adt-2(wk156) X; hdIs26 III*		This study
VQ1244	*sax-7(qv30) IV; mig-6(qv33) V; adt-2(wk156) X; hdIs26 III*		This study
VQ1436	*oyIs14 V; qyIs46 X*		This study
VQ2067	*sax-7(qv30); oyIs14; qyIs46*		This study
**Rescue assays with *mig-6S* minigene**			
VQ1138	*sax-7(qv30) IV; mig-6(qv33) V; hdIs26 III; qvEx336*	P*mig-6::mig-6S*	This study
VQ1139	*sax-7(qv30) IV; mig-6(qv33) V; hdIs26 III; qvEx337*	P*mig-6::mig-6S*	This study
**Rescue assays with recombinant versions of MIG-6S**			
VQ1927	*sax-7(qv30) IV; mig-6(qv33) V; hdIs26 III; qvEx608*	P*myo-3::mig-6SΔPapilin cassette/Lagrin repeats*	This study
VQ1928	*sax-7(qv30) IV; mig-6(qv33) V; hdIs26 III; qvEx609*	P*myo-3::mig-6SΔPapilin cassette/Lagrin repeats*	This study
VQ1936	*sax-7(qv30) IV; mig-6(qv33) V; hdIs26 III; qvEx610*	Pmyo-3::mig-6SΔ*Kunitz protease inhibitor domains*	This study
VQ1960	*sax-7(qv30) IV; mig-6(qv33) V; hdIs26 III; qvEx615*	P*myo-3::mig-6SΔKunitz protease inhibitor domains*	This study
**Control strains for rescue assays with recombinant versions of MIG-6S**			
VQ1961	*hdIs26 III; qvEx616*	P*myo-3::mig-6SΔKunitz protease inhibitor domains*	This study
VQ1986	*hdIs26 III; qvEx607*	P*myo-3::mig-6SΔKunitz protease inhibitor domains*	This study
**Tissue-specific rescue assays**			
VQ1656	*sax-7(qv30) IV; mig-6(qv33) V; hdIs26 III; qvEx520*	P*rgef-1::mig-6S*	This study
VQ1657	*sax-7(qv30) IV; mig-6(qv33) V; hdIs26 III; qvEx521*	P*rgef-1::mig-6S*	This study
VQ1347	*sax-7(qv30) IV; mig-6(qv33) V; hdIs26 III; qvEx522*	P*rgef-1::mig-6S*	This study
VQ1659	*sax-7(qv30) IV; mig-6(qv33) V; hdIs26 III; qvEx523*	P*rgef-1::mig-6S*	This study
VQ1661	*sax-7(qv30) IV; mig-6(qv33) V; hdIs26 III; qvEx525*	P*rgef-1::mig-6S*	This study
VQ1662	*sax-7(qv30) IV; mig-6(qv33) V; hdIs26 III; qvEx526*	P*rgef-1::mig-6S*	This study

VQ1348	*sax-7(qv30) IV; mig-6(qv33) V; hdIs26 III; qvEx375*	P*dpy-7::mig-6S*	This study
VQ1663	*sax-7(qv30) IV; mig-6(qv33) V; hdIs26 III; qvEx527*	P*dpy-7::mig-6S*	This study
VQ1664	*sax-7(qv30) IV; mig-6(qv33) V; hdIs26 III; qvEx528*	P*dpy-7::mig-6S*	This study
VQ1378	*sax-7(qv30) IV; mig-6(qv33) V; hdIs26 III; qvEx394*	P*dpy-7::mig-6S*	This study
VQ1674	*sax-7(qv30) IV; mig-6(qv33) V; hdIs26 III; qvEx538*	P*dpy-7::mig-6S*	This study
VQ1675	*sax-7(qv30) IV; mig-6(qv33) V; hdIs26 III; qvEx539*	P*dpy-7::mig-6S*	This study
VQ1346	*sax-7(qv30) IV; mig-6(qv33) V; hdIs26 III; qvEx373*	P*myo-3::mig-6S*	This study
VQ1940	*sax-7(qv30) IV; mig-6(qv33) V; hdIs26 III; qvEx611*	P*myo-3::mig-6S*	This study
VQ1669	*sax-7(qv30) IV; mig-6(qv33) V; hdIs26 III; qvEx533*	P*myo-3::mig-6S*	This study
VQ1344	*sax-7(qv30) IV; mig-6(qv33) V; hdIs26 III; qvEx372*	P*myo-3::mig-6S*	This study
VQ1380	*sax-7(qv30) IV; mig-6(qv33) V; hdIs26 III; qvEx396*	P*myo-3::mig-6S*	This study
VQ1396	*sax-7(qv30) IV; mig-6(qv33) V; hdIs26 III; qvEx405*	P*myo-3::mig-6S*	This study
VQ1536	*sax-7(qv30) IV; mig-6(qv33) mig-17(k174) V; hdIs26 III; qvEx396*	P*myo-3::mig-6S*	This study
VQ1537	*sax-7(qv30) IV; mig-6(qv33) mig-17(k174) V; hdIs26 III; qvEx405*	P*myo-3::mig-6S*	This study
**Control strains for tissue-specific rescue assays**			
VQ1389	*hdIs26 III; qvEx402*	P*rgef-1::mig-6S*	This study
VQ1670	*hdIs26 III; qvEx534*	P*rgef-1::mig-6S*	This study
VQ1383	*hdIs26 III; qvEx399*	P*rgef-1::mig-6S*	This study
VQ1671	*hdIs26 III; qvEx535*	P*rgef-1::mig-6S*	This study
VQ1672	*hdIs26 III; qvEx536*	P*rgef-1::mig-6S*	This study
VQ1673	*hdIs26 III; qvEx537*	P*rgef-1::mig-6S*	This study
VQ1676	*hdIs26 III; qvEx540*	P*dpy-7::mig-6S*	This study
VQ1390	*hdIs26 III; qvEx403*	P*dpy-7::mig-6S*	This study
VQ1391	*hdIs26 III; qvEx404*	P*dpy-7::mig-6S*	This study
VQ1677	*hdIs26 III; qvEx541*	P*dpy-7::mig-6S*	This study
VQ1678	*hdIs26 III; qvEx542*	P*dpy-7::mig-6S*	This study
VQ1665	*hdIs26 III; qvEx529*	P*myo-3::mig-6S*	This study
VQ1666	*hdIs26 III; qvEx530*	P*myo-3::mig-6S*	This study
VQ1381	*hdIs26 III; qvEx397*	P*myo-3::mig-6S*	This study
VQ1667	*hdIs26 III; qvEx531*	P*myo-3::mig-6S*	This study
VQ1382	*hdIs26 III; qvEx398*	P*myo-3::mig-6S*	This study
VQ1668	*hdIs26 III; qvEx532*	P*myo-3::mig-6S*	This study
**Collagen IV phenotype characterization**			
VQ1176	*mig-6(qv33) V; qyIs46 X*		This study
VQ1570	*mig-6(k177) V; qyIs46 X*		This study
VQ1544	*mig-6(e1931)/dpy-11(e224) oyIs14 V; qyIs46 X*		This study
VQ1443	*mig-17(k174) V; qyIs46 X*		This study
VQ1498	*mig-6(qv33) mig17(k174) V; qyIs46 X*		This study
VQ1477	*mig-6(qv33)/dpy-11(e224) oyIs14 V; qyIs46 X*		This study

VQ1563	*mig-17(k174)/dpy-11(e224) oyIs14 V ; qyIs46 X*		This study
VQ1481	*mig-6(qv33) mig-17(k174)/dpy-11 oyIs14 V; qyIs46 X*		This study
VQ1470	*evIs213; qyIs46 X*		This study
VQ1562	*mig-6(qv33) V; evIs213; qyIs46 X*		This study
VQ1202	*sax-7(qv30) IV; qyIs46 X*		This study
VQ1215	*sax-7(qv30) IV; mig-6(qv33) V; qyIs46 X*		This study
VQ1573	*sax-7(qv30) IV; mig-6(k177) V; qyIs46 X*		This study
VQ1516	*sax-7(qv30) IV; mig-17(k174) V; qyIs46 X*		This study
**Rescue assays for collagen IV fibrotic phenotype**			
VQ1428	*mig-6(qv33) V; qyIs46 X; qvEx434*	P*mig-6::mig-6S*	This study
**PXN-2 expression pattern**			
VQ1547	*mig-6(qv33) V; qy76 X*		This study
**Body wall muscles and other mesodermal cells marker**			
VQ1448	*qyIs46 X; dnIs13; him-5(e1490) V*		This study
VQ1463	*mig-6(qv33) V; qyIs46; dnIs13*		This study
**Body wall muscles structure characterization**			
VQ2301	*mig-6(qv33)V; stEx30*		This study

1 Brenner, S. The genetics of Caenorhabditis elegans. *Genetics* **77**, 71-94 (1974).
2 Hutter, H. Extracellular cues and pioneers act together to guide axons in the ventral cord of C. elegans. (2003).
3 Sarafi-Reinach, T. R., Melkman, T., Hobert, O. & Sengupta, P. The lin-11 LIM homeobox gene specifies olfactory and chemosensory neuron fates in C. elegans. (2001).
4 Kawano, T. *et al.* C. elegans mig-6 encodes papilin isoforms that affect distinct aspects of DTC migration, and interacts genetically with mig-17 and collagen IV. (2009).
5 Ihara, S. *et al.* Basement membrane sliding and targeted adhesion remodels tissue boundaries during uterine–vulval attachment in Caenorhabditis elegans. *Nature cell biology* **13**, 641-651 (2011).
6 Keeley, D. P. *et al.* Comprehensive endogenous tagging of basement membrane components reveals dynamic movement within the matrix scaffolding. *Developmental cell* **54**, 60-74. e67 (2020).
7 Levy-Strumpf, N. & Culotti, J. G. Netrins and Wnts function redundantly to regulate antero-posterior and dorso-ventral guidance in C. elegans. *PLoS genetics* **10**, e1004381 (2014).
8 Bénard, C., Tjoe, N., Boulin, T., Recio, J. & Hobert, O. The small, secreted immunoglobulin protein ZIG-3 maintains axon position in Caenorhabditis elegans. *Genetics* **183**, 917-927 (2009).
9 Campagnola, P. J., Millard, A. C., Terasaki, M., Hoppe, P. E., Malone, C. J. & Mohler, W. A. Three-dimensional high-resolution second-harmonic generation imaging of endogenous structural proteins in biological tissues. *Biophysical journal* **82**, 493-508 (2002).

S2 Table. Detailed information on transgenic strains used.

Strain	Genotype	Transgene	Reference
**Rescue assay with *mig-6S* minigene**			
VQ1138	*sax-7(qv30) IV; mig-6(qv33) V; hdIs26 III; qvEx336*	pCB202 [P*mig-6::mig-6S*] at 5 ng/µL, *ttx-3::mcherrry*, pBSK+. Line 1	This study
VQ1139	*sax-7(qv30) IV; mig-6(qv33) V; hdIs26 III; qvEx337*	pCB202 [P*mig-6::mig-6S*] at 5 ng/µL, *ttx-3::mcherrry*, pBSK+. Line 2	This study
**Rescue assays with recombinant versions of MIG-6S**			
VQ1927	*sax-7(qv30) IV; mig-6(qv33) V; hdIs26 III; qvEx608*	pCB483 [P*myo-3::mig-6S∆papilin cassette/lagrin repeats*] 1 ng/µL, pHP6 [P*lgc-11::GFP*], pBSK+. Line #1	This study
VQ1928	*sax-7(qv30) IV; mig-6(qv33) V; hdIs26 III; qvEx609*	pCB483 [P*myo-3::mig-6S∆papilin cassette/lagrin repeats*] 1 ng/µL, pHP6 [P*lgc-11::GFP*], pBSK+. Line #2	This study
VQ1936	*sax-7(qv30) IV; mig-6(qv33) V; hdIs26 III; qvEx610*	pCB492 [P*myo-3::mig-6S∆Kunitz domains]* 1 ng/µL, pHP6 [P*lgc-11::GFP],* pBSK+. Line #1	This study
VQ1960	*sax-7(qv30) IV; mig-6(qv33) V; hdIs26 III; qvEx615*	pCB492 [P*myo-3::mig-6S∆Kunitz domains*] 1 ng/µL, pHP6 [P*lgc-11::GFP*], pBSK+. Line #2	This study
**Control strains for rescue assays with recombinant versions of MIG-6S**			
VQ1961	*hdIs26 III; qvEx616*	pCB492 [P*myo-3::mig-6S∆Kunitz domains*] 1 ng/µL, pHP6 [P*lgc-11::GFP*], pBSK+. Line 1	This study
VQ1986	*hdIs26 III; qvEx617*	pCB492 [P*myo-3::mig-6S∆Kunitz domains*] 1 ng/µL, pHP6 [P*lgc-11::GFP*], pBSK+. Line 2	This study
**Tissue-specific rescue assays**			
VQ1656	*sax-7(qv30) IV; mig-6(qv33) V; hdIs26 III; qvEx520*	pCB408 [P*rgef-1::mig-6S*] at 7 ng/µL, P*lgc-11::GFP,* pBSK+. Line #1	This study
VQ1657	*sax-7(qv30) IV; mig-6(qv33) V; hdIs26 III; qvEx521*	pCB408 [P*rgef-1::mig-6S*] at 7 ng/µL, P*lgc-11::GFP*, pBSK+. Line #2	This study
VQ1347	*sax-7(qv30) IV; mig-6(qv33) V; hdIs26 III; qvEx522*	pCB408 [P*rgef-1::mig-6S*] at 7 ng/µL, P*lgc-11::GFP*, pBSK+. Line #3	This study
VQ1659	*sax-7(qv30) IV; mig-6(qv33) V; hdIs26 III; qvEx523*	pCB408 [P*rgef-1::mig-6S*] at 7 ng/µL, P*lgc-11::GFP*, pBSK+. Line #4	This study
VQ1661	*sax-7(qv30) IV; mig-6(qv33) V; hdIs26 III; qvEx525*	pCB408 [P*rgef-1::mig-6S*] at 7 ng/µL, P*lgc-11::GFP*, pBSK+. Line #5	This study
VQ1662	*sax-7(qv30) IV; mig-6(qv33) V; hdIs26 III; qvEx526*	pCB408 [P*rgef-1::mig-6S*] at 7 ng/µL, P*lgc-11::GFP*, pBSK+. Line #6	This study
VQ1348	*sax-7(qv30) IV; mig-6(qv33) V; hdIs26 III; qvEx375*	pCB409 [P*dpy-7::mig-6S*] at 0.5 ng/µL, P*lgc-11::GFP*, pBSK+. *Line* #7	This study
VQ1663	*sax-7(qv30) IV; mig-6(qv33) V; hdIs26 III; qvEx527*	pCB409 [P*dpy-7::mig-6S*] at 0.5 ng/µL, P*lgc-11::GFP*, pBSK+. Line #8	This study
VQ1664	*sax-7(qv30) IV; mig-6(qv33) V; hdIs26 III; qvEx528*	pCB409 [P*dpy-7::mig-6S*] at 0.5 ng/µL, P*lgc-11::GFP*, pBSK+. Line #9	This study
VQ1378	*sax-7(qv30) IV; mig-6(qv33) V; hdIs26 III; qvEx394*	pCB409 [P*dpy-7::mig-6S*] at 0.5 ng/µL, P*lgc-11::GFP*, pBSK+. *Line* #10	This study
VQ1674	*sax-7(qv30) IV; mig-6(qv33) V; hdIs26 III; qvEx538*	pCB409 [P*dpy-7::mig-6S*] at 0.1 ng/µL, P*lgc-11::GFP*, pBSK+. Line #11	This study
VQ1675	*sax-7(qv30) IV; mig-6(qv33) V; hdIs26 III; qvEx539*	pCB409 [P*dpy-7::mig-6S*] at 0.1 ng/µL, P*lgc-11::GFP*, pBSK+. Line #12	This study
VQ1346	*sax-7(qv30) IV ; mig-6(qv33) V; hdIs26 III; qvEx373*	pCB416 [P*myo-3::mig-6S*] at 1 ng/µL, P*ceh-22::GFP*, pBSK+. Line #13	This study
VQ1940	*sax-7(qv30) IV ; mig-6(qv33) V; hdIs26 III; qvEx611*	pCB416 [P*myo-3::mig-6S*] at 1 ng/µL, P*ceh-22::GFP*, pBSK+. Line #14	This study
VQ1669	*sax-7(qv30) IV; mig-6(qv33) V; hdIs26 III; qvEx533*	pCB416 [P*myo-3::mig-6S*] at 1 ng/µL, P*lgc-11::GFP*, pBSK+. Line #15	This study
VQ1344	*sax-7(qv30) IV ; mig-6(qv33) V; hdIs26 III; qvEx372*	pCB416 [P*myo-3::mig-6S*] at 1 ng/µL, P*ceh-22::GFP*, pBSK+. Line #16	This study

VQ1380	*sax-7(qv30) IV; mig-6(qv33) V; hdIs26 III; qvEx396*	pCB416 [P*myo-3::mig-6S*] at 1 ng/µL, P*lgc-11::GFP*, pBSK+. Line #17	This study
VQ1396	*sax-7(qv30) IV; mig-6(qv33) V; hdIs26 III; qvEx405*	CB416 [P*myo-3::mig-6S*] at 1 ng/µL, P*lgc-11::GFP*, pBSK+. Line #18	This study
**Dependency of *mig-6S* rescue on *mig-17* function**			
VQ1536	*sax-7(qv30) IV; mig-6(qv33) mig-17(k174) V; hdIs26 III; qvEx396*	pCB416 [P*myo-3::mig-6S*] at 1 ng/µL , P*lgc-11::GFP*, pBSK+. Line #17	This study
VQ1537	*sax-7(qv30) IV; mig-6(qv33) mig-17(k174) V; hdIs26 III*; *qvEx405*	pCB416 [P*myo-3::mig-6S*] at 1 ng/µL, P*lgc-11::GFP*, pBSK+. Line #18	This study
**Control strains for tissue-specific rescue assays**			
VQ1389	*hdIs26 III; qvEx402*	pCB408 [P*rgef-1::mig-6S*] at 7 ng/µL, P*lgc-11::GFP*, pBSK+. Line #1	This study
VQ1670	*hdIs26 III; qvEx534*	pCB408 [P*rgef-1::mig-6S*] at 7 ng/µL , P*lgc-11::GFP* pBSK+. Line #2	This study
VQ1383	*hdIs26 III; qvEx399*	pCB408 [P*rgef-1::mig-6S*] at 7 ng/µL, P*lgc-11::GFP*, pBSK+. Line #3	This study
VQ1671	*hdIs26 III; qvEx535*	pCB408 [P*rgef-1::mig-6S*] at 7 ng/µL, P*lgc-11::GFP*, pBSK+. Line #4	This study
VQ1672	*hdIs26 III; qvEx536*	pCB408 [P*rgef-1::mig-6S*] at 7 ng/µL, P*lgc-11::GFP*, pBSK+. Line #5	This study
VQ1673	*hdIs26 III; qvEx537*	pCB408 [P*rgef-1::mig-6S*] at 7 ng/µL, P*lgc-11::GFP*, pBSK+. Line #6	This study
VQ1676	*hdIs26 III; qvEx540*	pCB409 [P*dpy-7::mig-6S*] at 0.1 ng/µL, P*lgc-11::GFP*, pBSK+. Line # 1	This study
VQ1390	*hdIs26 III; qvEx403*	pCB409 [P*dpy-7::mig-6S*] at 0.1 ng/µL, P*lgc-11::GFP*, pBSK+. Line #2	This study
VQ1391	*hdIs26 III; qvEx404*	pCB409 [P*dpy-7::mig-6S*] at 0.1 ng/µL, P*lgc-11::GFP*, pBSK+. Line #3	This study
VQ1677	*hdIs26 III; qvEx541*	pCB409 [P*dpy-7::mig-6S*] at 0.1 ng/ µL, P*lgc-11::GFP*, pBSK+. Line #4	This study
VQ1678	*hdIs26 III; qvEx542*	pCB409 [P*dpy-7::mig-6S*] at 0.1 ng/µL, P*lgc-11::GFP*, pBSK+. Line #5	This study
VQ1665	*hdIs26 III; qvEx529*	pCB416 [P*myo-3::mig-6S*] at 1 ng/µL, P*lgc-11::GFP*, pBSK+. Line #1	This study
VQ1666	*hdIs26 III; qvEx530*	pCB416 [P*myo-3::mig-6S*] at 1 ng/µL, P*lgc-11::GFP*, pBSK+. Line #2	This study
VQ1381	*hdIs26 III; qvEx397*	pCB416 [P*myo-3::mig-6S*] at 1 ng/µL, P*lgc-11::GFP*, pBSK+. Line #3	This study
VQ1667	*hdIs26 III; qvEx531*	pCB416 [P*myo-3::mig-6S*] at 1 ng/µL, P*lgc-11::GFP rfp*, pBSK+. Line #4	This study
VQ1382	*hdIs26 III; qvEx398*	pCB416 [P*myo-3::mig-6S*] at 1 ng/µL, P*lgc-11::GFP*, pBSK+. Line #5	This study
VQ1668	*hdIs26 III; qvEx532*	pCB416 [P*myo-3::mig-6S*] at 1 ng/µL, P*lgc-11::GFP*, pBSK+. Line #6	This study
**Rescue assays of collagen IV fibrotic phenotype**			
VQ1428	*mig-6(qv33) V; qyIs46 X; qvEx434*	pZH125.3 [P*mig-6::mig-6S*] at 5 ng/µL P*lgc-11::GFP*, pBSK+. Line 1	This study

S3 Table. List of primers used.

Gene	Primer	Sequence	PCR product (bp)
sax-7(qv30)			
Mutant specific	oCB747	tctctcaaaattcttcgcaagc	336
	oCB1025	cgggaagaaatgaaacagga	
Wild-type specific	oCB1022	tggtggtagcgatggtgtag	609
	oCB1023	agttcgatgttctcggctgt	
mig-6(qv33)			
	oCB2241	ctcccaaggaagagcctatcc	586
	oCB2242	cgagcagttagagcatccg	
BamHI digestion			
Mutant specific			586
Wild-type specific			203 and 383
***mig-6(k177)* sequencing**			
	oCB1602	acacggacacaagactcgtcg	501
	0CB1603	ttggcaacatcagcttcaagg	
***mig-6(ev700)* sequencing**			
	oCB1574	gttgagtgtgccaccattgc	357
	oCBQc21	gaaggatgatcgctcacatcc	
***mig-6(ev701)* sequencing**			
	oCB1574	gttgagtgtgccaccattgc	357
	oCBQc21	gaaggatgatcgctcacatcc	
***mig-6(sa580)* sequencing**			
	oCB1571	cgaagaccgaattcggatgc	547
	oCB1572	gcacgagtctttccgtctgg	
***adt-2(ok595)* sequencing**			
	OCB1388	ttaccagacaaccggtaggg	554
	OCB1389	tgatgacatgatatgcttgg	
***zig-3(tm924)* sequencing**			
Mutant specific	oCBQc39	aaaaatgctgctcatctgcatatctgtcc	
	oCBQc40	Ttaagcaatatgttttttggtggg	544
Wild-type specific	oCBQc40	gggtcctacagtcgagtcagg	413
	oCBQc38	ccactggattttccggatgagaa	
***zig-4(gk34)* sequencing**			
Mutant specific	oCBQc43	ggcccgttcaggagtacaacgacaacacag	
	oCBQc42	Cgcggttgggtaaaggaatgtttcttggcg	924
Wild-type specific	oCBQc41	atgtgatataatgtccactcctgctgctacc	
	oCBQc42	cgcggttgggtaaaggaatgtttcttggcg	596
zig-5(ok1065)			
Mutant specific	oTB34	aatgctagcggtaccatgtttcgtcctatccg	721
	oTB52	gcatgttcccgttatcgattttggcg	
Wild-type specific	oTB34	aatgctagcggtaccatgtttcgtcctatccg	469
	oCB120	ctcgaaataggtacatgtcaacc	
zig-8(ok561)			
Mutant specific	oCB210	aataagatcgctaaccgttataag	335
	oCB209	gcaagagcgataataggtagg	
Wild-type specific	oCB201	gtgtaggctaggaaatcggtggg	316
	oCB209	gcaagagcgataataggtagg	

Description of Additional Supplementary Files

File Name: Supplementary Movie 1
Description: *mig-6(qv33)* mutant animals display increased collagen IV and fibrotic-like structures phenotype. Projection of fluorescence images of the head region in a 2-day-old adult *mig-6(qv33)* mutant animal expressing collagen IV reporter EMB-9::mCherry (*qyIs46)*. Collagen IV can be seen as (i) a layer surrounding the spherical terminal bulb of the pharynx, (ii) a ring at the level of the pharyngo-intestinal valve, (iii) numerous and intense small circular accumulations are inside muscle cells, (iv) prominent elongated fibrotic-like structures around the general area of the terminal bulb of the pharynx, and (v) faint lines along muscle cells.

File Name: Supplementary Movie 2
Description: *mig-6(qv33)* mutant animals display increased collagen IV and fibrotic-like structures phenotype. Projection of fluorescence images of the region of the terminal bulb of the pharynx in the head of a 2-day-old adult *mig-6(qv33)* mutant animal expressing collagen IV reporter EMB-9::mCherry (*qyIs46)*. Collagen IV can be seen as (i) a layer surrounding the spherical terminal bulb of the pharynx, (ii) a ring at the level of the pharyngo-intestinal valve, (iii) numerous and intense small circular accumulations are inside muscle cells, and (iv) prominent elongated fibrotic-like structures around the general area of the terminal bulb of the pharynx.

File Name: Supplementary Movie 3
Description: Normal pattern of collagen IV in the wild type. Projection of fluorescence images of the region of the terminal bulb of the pharynx in the head of a 2-day-old adult wild-type animal expressing collagen IV reporter EMB-9::mCherry (*qyIs46)*. Collagen IV can be seen as (i) a layer surrounding the spherical terminal bulb of the pharynx, (ii) a ring at the level of the pharyngo-intestinal valve, and (iii) small circular accumulations are inside muscle cells.

Supplementary Files

This is a list of supplementary files associated with this preprint. Click to download.

- SourcedataFIGURESNadouretal.xlsx
- SourcedataSUPPLEMENTARYFIGURESNadouretal.xlsx
- mig6qv33qyIs46mutantanimalWHOLEHEAD.avi
- mig6qv33qyIs46mutantanimalTERMINALBULBregion.avi
- WildtypeqyIs46animalTERMINALBULBregion.avi
